# Supplementary material for: Discovery of Host-Directed Small Molecules with Broad Anti-Leishmanial Efficacy
Source: bioRxiv. 2025 Nov 4:2025.11.04.686469. Preprint. [Version 1] doi: 10.1101/2025.11.04.686469 (PMC12637588; doi:10.1101/2025.11.04.686469)
Supplement: 1 [file NIHPP2025.11.04.686469v1-supplement-1.pdf]

bioRxiv preprint doi: https://doi.org/10.1101/2025.11.04.686469; this version posted November 4, 2025. The copyright holder for this preprint (which was not certified by peer review) is the author/funder, who has granted bioRxiv a license to display the preprint in perpetuity. It is made available under aCC-BY 4.0 International license.

SUPPLEMENTAL INFORMATION CAPTIONS:

Supplemental Table 1. Chemical Structures of all compounds screened.

Supplemental Figure 1. Initial Screening Approach. Schematic illustrating the medium-throughput luminescence-based assay to determine effect of compounds on intracellular *Leishmania* infection and parallel screening to determine effect of compounds on host cell viability.

Supplemental Table 2. Results of primary screen in all compounds. Concentration at which intracellular *Leishmania donovani* burden is reduced by 50% in THP1 macrophages (Lum IC_{50}) as identified by luminescence assay. Concentration where THP1 macrophage cell viability is 50% (LC_{50}) after 24-hour incubation with compound as determined by MTT assay. Selectivity between host-directed effect and cytotoxicity, defined as 24h LC_{50} / Lum IC_{50}. Parental compound AR-12 provided for reference. Compounds highlighted in grey have higher selectivity than parental compound AR-12. Compounds highlighted in yellow were selected for secondary screening. ND = not determined.

Supplemental Figure 2. Venn diagram demonstrating compound potency against intracellular *L. donovani* (Lum IC_{50}) and cytotoxicity against THP-1 host cell (24 hr LC_{50}) relative to parental compound AR-12.

Supplemental Figure 3. Luminescent activity of intracellular *L. donovani* infected THP1 macrophage cell after 72-hour incubation with compounds.

Supplemental Figure 4. Graphs of THP1 macrophage cell viability after 24-hour incubation with compounds as determined by MTT assay.

Supplemental Figure 5. Graphs of THP1 macrophage cell viability after 72-hour incubation with compounds as determined by MTT assay.

bioRxiv preprint doi: https://doi.org/10.1101/2025.11.04.686469; this version posted November 4, 2025. The copyright holder for this preprint (which was not certified by peer review) is the author/funder, who has granted bioRxiv a license to display the preprint in perpetuity. It is made available under aCC-BY 4.0 International license.

Supplemental Figure 6. Dose response of extracellular *Leishmania* promastigote viability after 72-hour incubation with compound as measured by resazurin assay.

Supplemental Figure 7. Dose response of intracellular *Leishmania donovani* burden in bone marrow derived macrophages after 72-hour incubation with compound as identified image-based giemsa staining.

Supplemental Figure 8. A) Chemical structure of 197 chemically modified for conjugation to agarose bead for affinity capture proteomic analysis. **B)** Luminescent activity of intracellular *L. donovani* infected THP1 macrophage cell after 72-hour incubation with 197 compared to chemically modified 197.

Supplemental Figure 9. A) All human proteins (gray circle) identified by affinity capture using 197 functionalized bead plotted Log2(fold-change) and -Log10(p-value) over control bead. Significant proteins Log2FC > 1 and $p < 0.05$ are shown with black circles. **B)** All human proteins (gray circle) identified by thermal profile analysis with 197 plotted z-score and -Log10(p-value). Significant proteins with a |z-score| > 1 and $p < 0.05$ are shown with black circles. **C)** All *Leishmania* proteins (gray circle) identified by affinity capture using 197 functionalized bead plotted Log2(fold-change) and -Log10(p-value) over control bead. Significant proteins Log2FC > 1 and $p < 0.05$ are shown with black circles. Proteins with a (|z-score| > 1 and $p < 0.05$ overlapping with significant proteins identified by affinity capture (Log2FC > 2 and $p < 0.05$) are shown with open circles. **D)** All *Leishmania* proteins (gray circle) identified by thermal profile analysis with 197 plotted z-score and -Log10(p-value). Significant proteins with a |z-score| > 1 and $p < 0.05$ are shown with black circles. Significant proteins Log2FC > 1 and $p < 0.05$ are shown with black circles. Proteins with a (|z-score| > 1 and $p < 0.05$ overlapping with significant proteins identified

bioRxiv preprint doi: https://doi.org/10.1101/2025.11.04.686469; this version posted November 4, 2025. The copyright holder for this preprint (which was not certified by peer review) is the author/funder, who has granted bioRxiv a license to display the preprint in perpetuity. It is made available under aCC-BY 4.0 International license.

by affinity capture (Log2FC > 2 and $p < 0.05$) are shown with open circles. **E)** Values for three *Leishmania* proteins overlapping between two proteomic approaches.

Supplemental Figure 10. Dose response of amphotericin B on intracellular *Leishmania* burden in bone marrow derived macrophages derived from wildtype C57BL/6 (WT, black circle) or lysozyme knockout mice (Lys K/O, gray triangle) as identified image-based Giemsa staining. Data is presented as mean ± standard deviation of biological triplicates.

Synthesis and Characterization of Selected Analogs.

bioRxiv preprint doi: https://doi.org/10.1101/2025.11.04.686469; this version posted November 4, 2025. The copyright holder for this preprint (which was not certified by peer review) is the author/funder, who has granted bioRxiv a license to display the preprint in perpetuity. It is made available under aCC-BY 4.0 International license.

Supplemental Information:

Synthesis and preliminary evaluation of novel compounds that demonstrate broad host-directed anti-leishmanial activity

Elizabeth Gurysh[a], M. Shamim Hasan Zahid[a], Monica M. Johnson[a], Antonio Landavazo[b], Ojas A Namjoshi [b], Joseph W Wilson [b], Devika M. Varma[a], Ryan N. Woodring[a], Aaron T. Hendricksen[a], Joseph F. Vath[a], Baiyi Quan[c], Erica N. Pino[a], Michael C. Fitzgerald[c], Eric M. Bachelder[a], Bruce E. Blough[b], Kristy M. Ainslie[acd]*

[a] Division of Pharmacoengineering and Molecular Pharmaceutics, Eshelman School of Pharmacy, University of North Carolina, Chapel Hill, NC.

[b] Center for Drug Discovery, RTI International, Research Triangle Park, Durham, NC

[c] Department of Chemistry, Duke University, Durham, NC,

[d] Joint Department of Biomedical Engineering, University of North Carolina, Chapel Hill, NC

[e] Department of Microbiology and Immunology, UNC School of Medicine, University of North Carolina, Chapel Hill, NC.

*Corresponding Author

Kristy M. Ainslie
Fred Eshelman Distinguished Professor and Chair
Division of Pharmacoengineering and Molecular Pharmaceutics
UNC Eshelman School of Pharmacy
4211 Marsico Hall, 125 Mason Farm Road
Chapel Hill, NC 27599, United States
ainsliek@email.unc.edu

Supplemental Table 1. Chemical Structures of all compounds screened.

AR--12	1	2	3	4	5	6
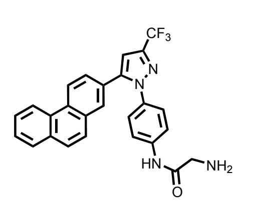	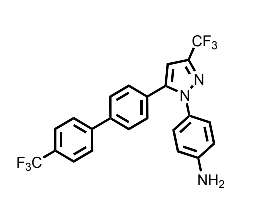	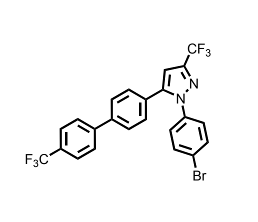	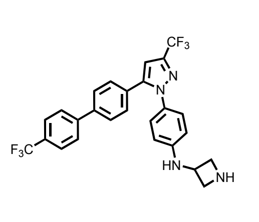	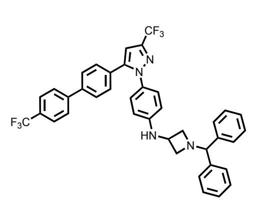	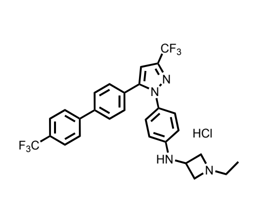	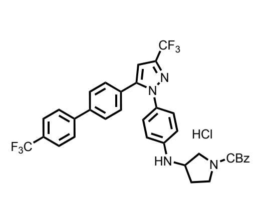
**7**	**8**	**9**	**10**	**11**	**12**	**13**
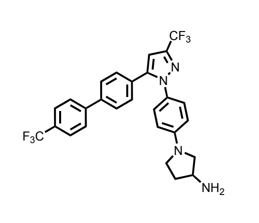	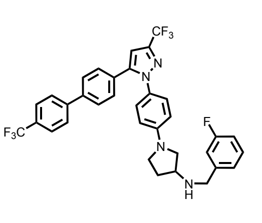	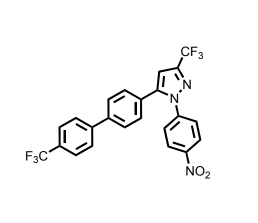	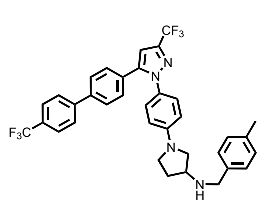	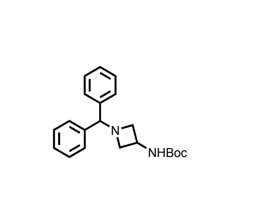	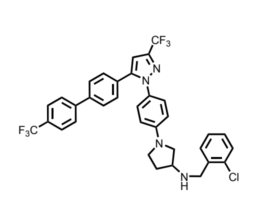	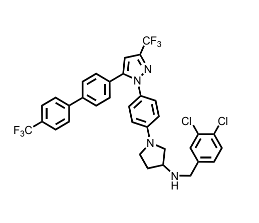
**14**	**15**	**16**	**17**	**18**	**19**	**20**
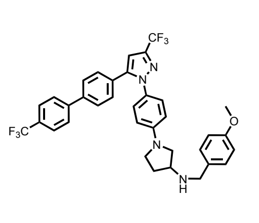	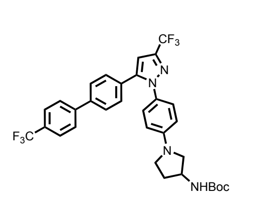	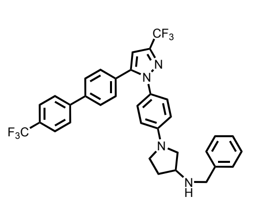	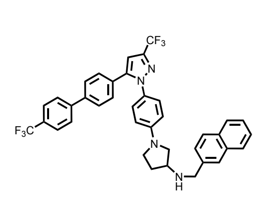	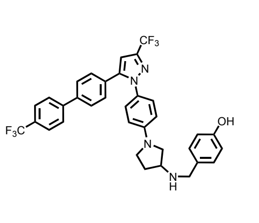	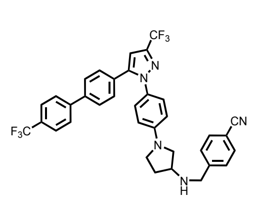	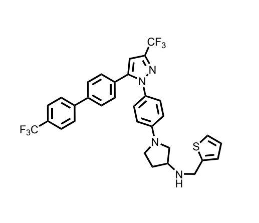
**21**	**22**	**23**	**24**	**25**	**26**	**27**
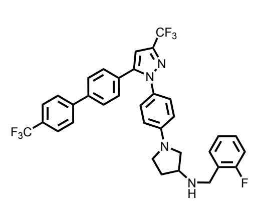	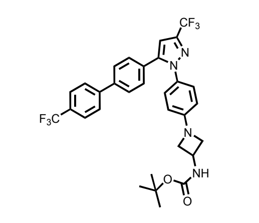	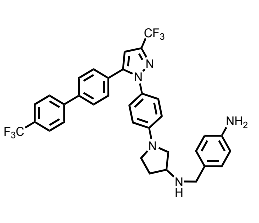	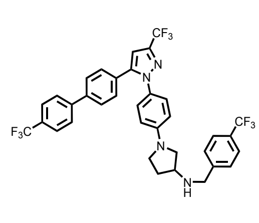	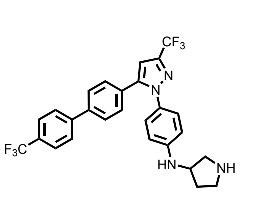	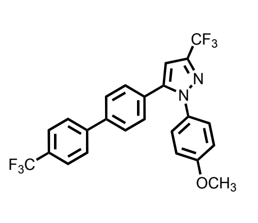	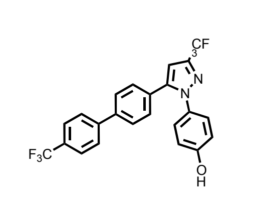
**28**	**29**	**30**	**31**	**32**	**33**	**34**
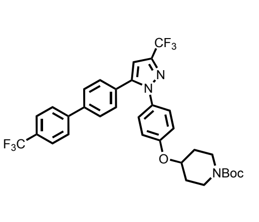	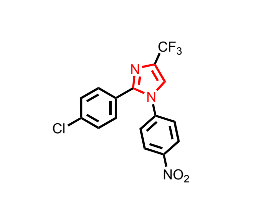	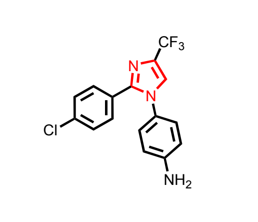	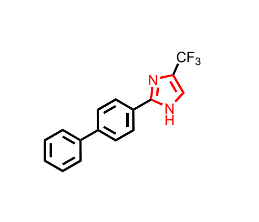	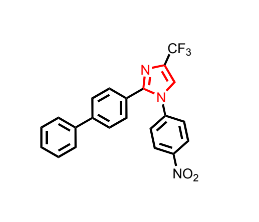	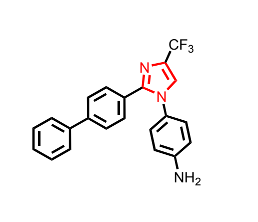	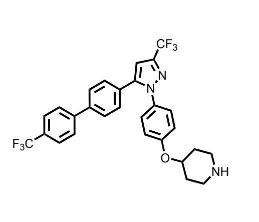

35	36	37	38	39	40	41
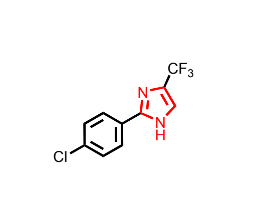	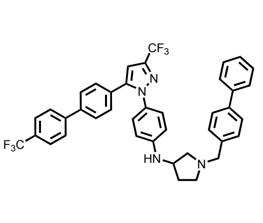	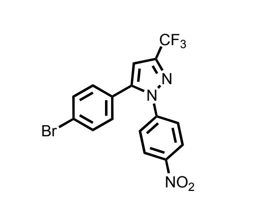	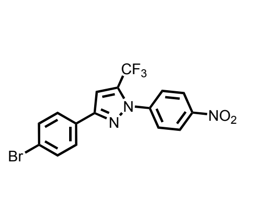	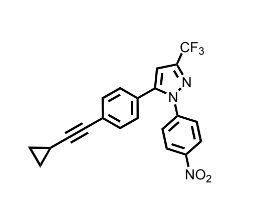	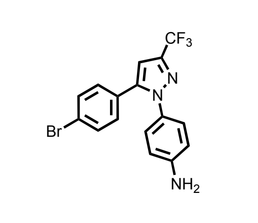	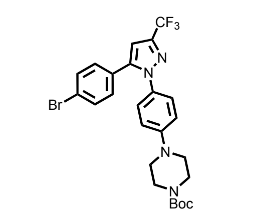
**42**	**43**	**44**	**45**	**46**	**47**	**48**
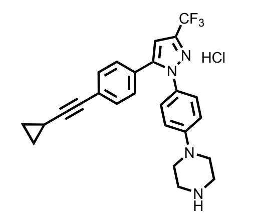	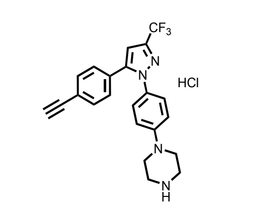	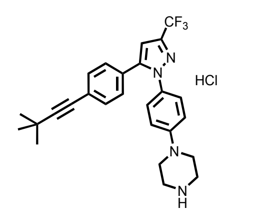	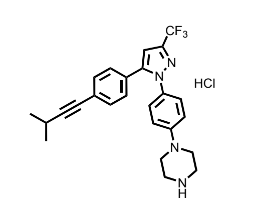	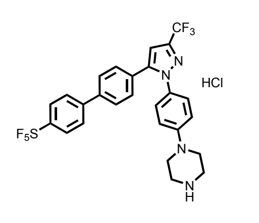	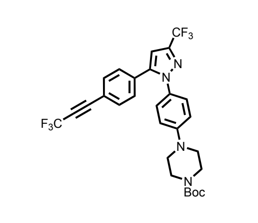	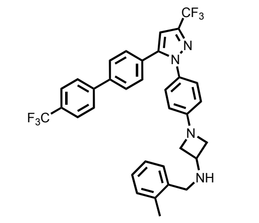
**49**	**50**	**51**	**52**	**53**	**54**	**55**
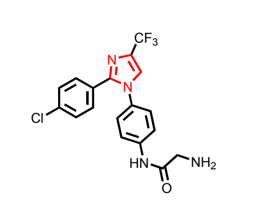	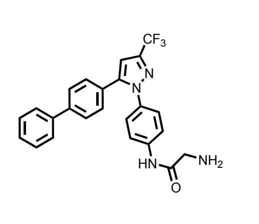	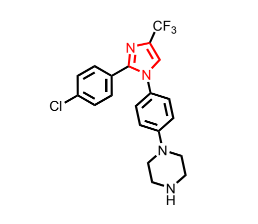	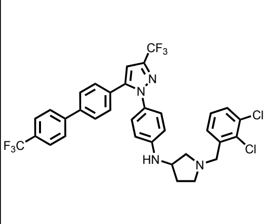	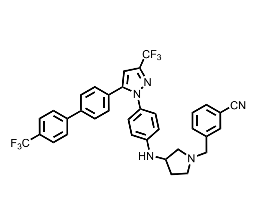	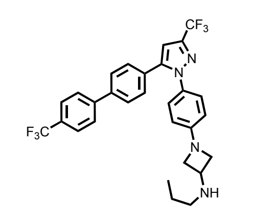	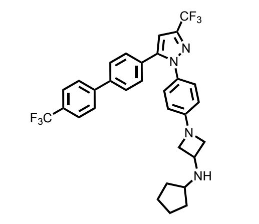
**56**	**57**	**58**	**59**	**60**	**61**	**62**
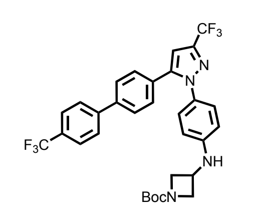	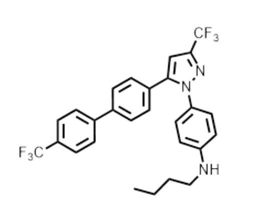	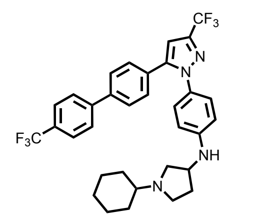	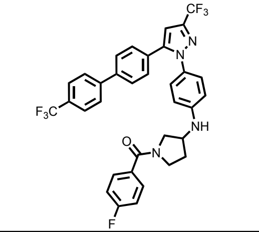	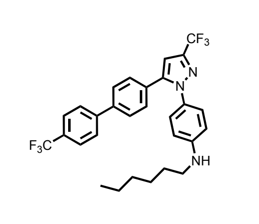	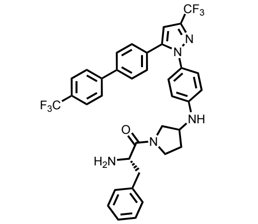	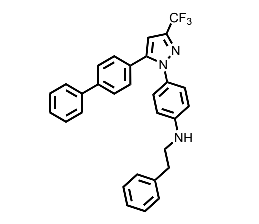
**63**	**64**	**65**	**66**	**67**	**68**	**69**
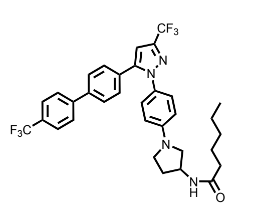	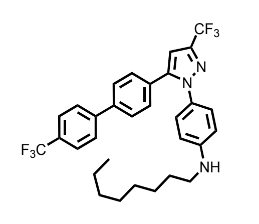	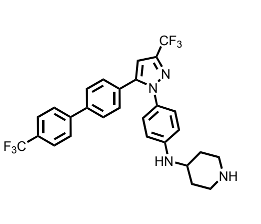	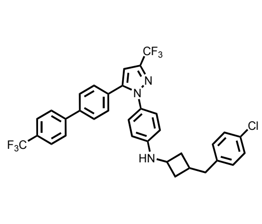	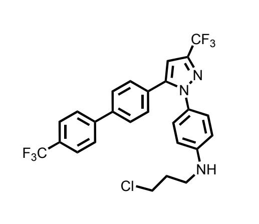	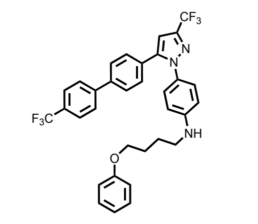	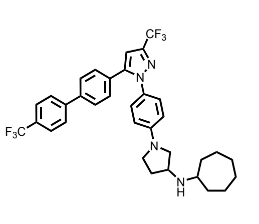

70	71	72	73	74	75	76
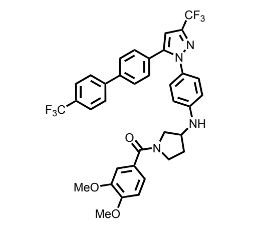	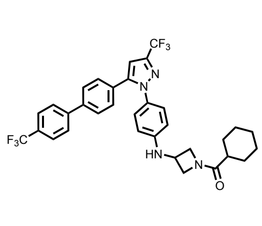	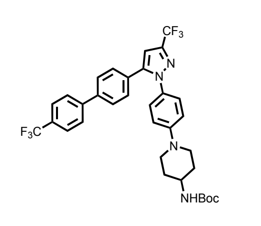	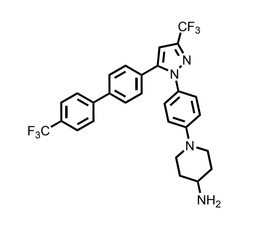	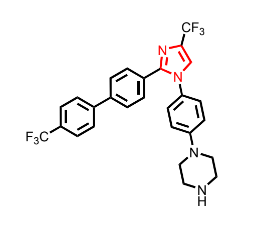	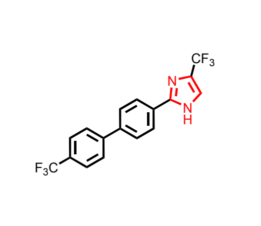	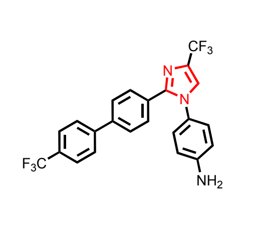

77	78	79	80	81	82	83
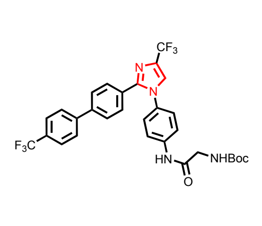	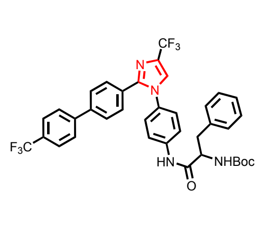	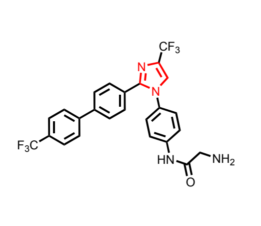	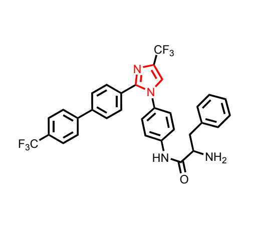	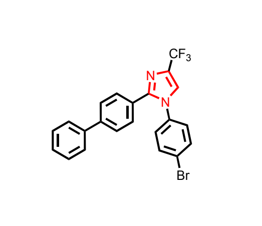	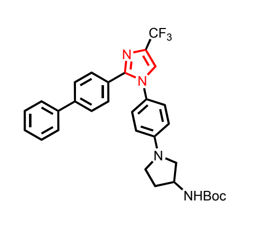	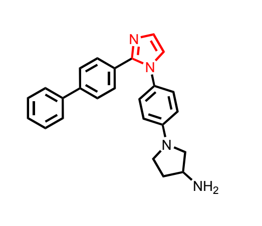

84	85	86	87	88	89	90
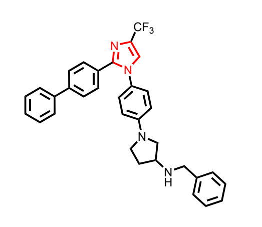	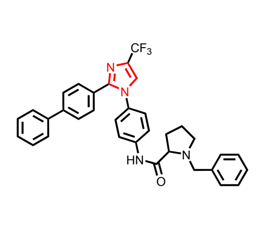	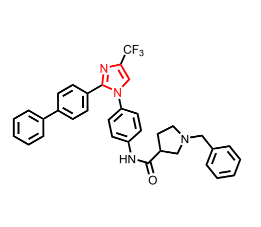	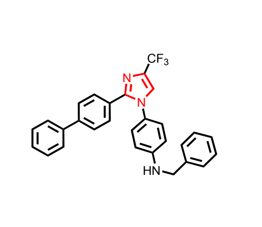	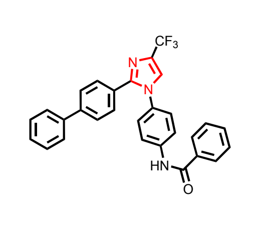	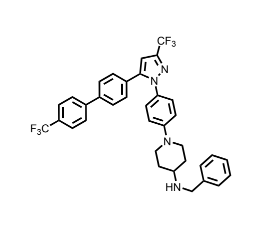	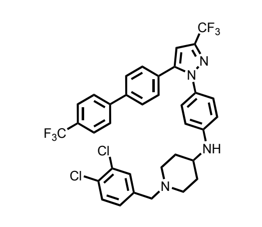

91	92	93	94	95	96	97
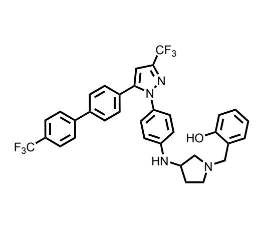	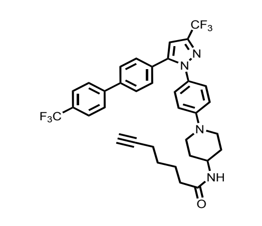	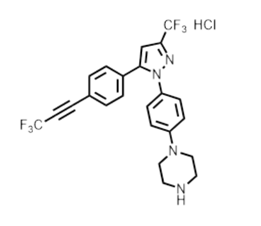	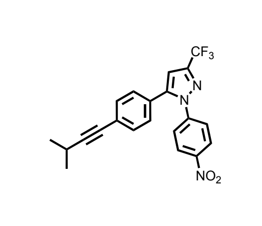	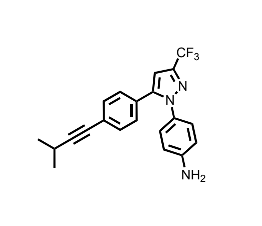	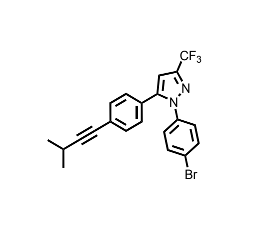	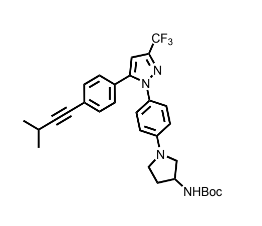

98	99	100	101	103	104	105
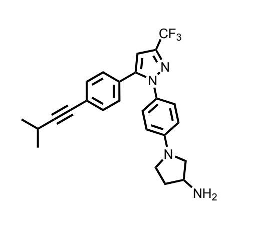	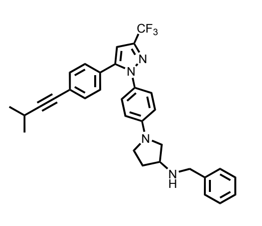	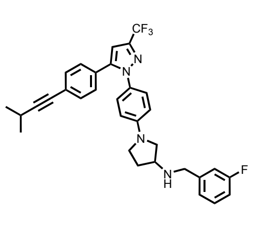	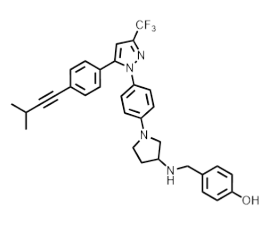	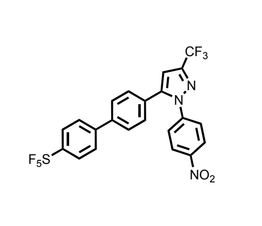	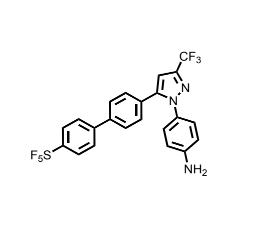	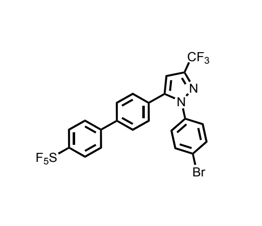

106	107	108	109	110	111	112
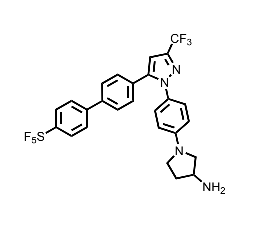	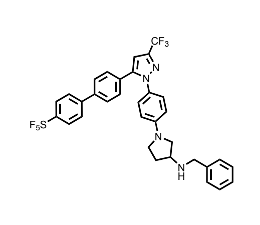	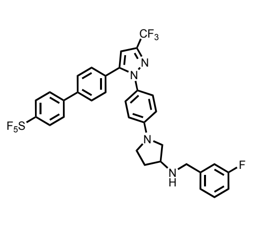	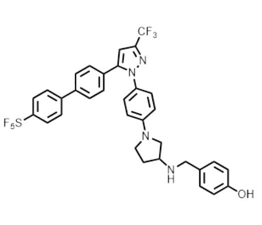	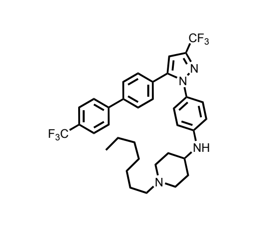	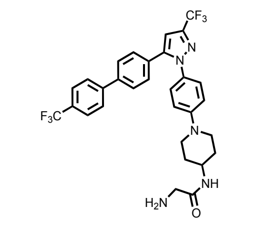	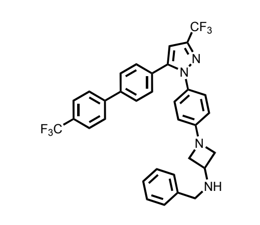
113	114	115	116	117	118	119
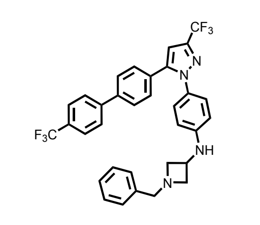	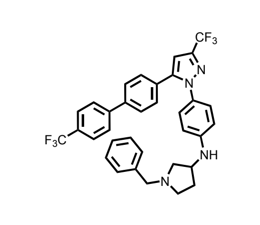	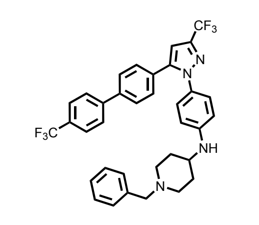	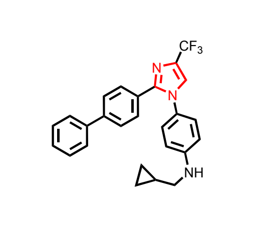	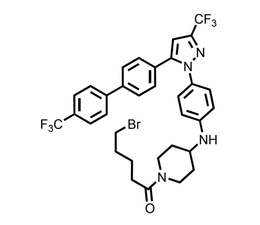	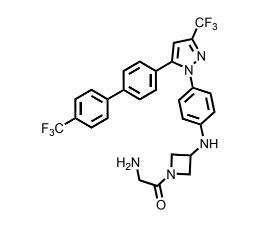	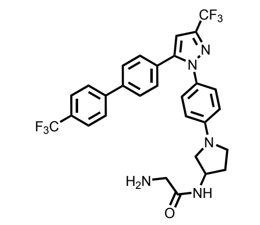
120	121	122	123	124	125	126
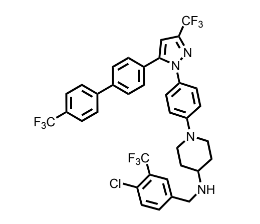	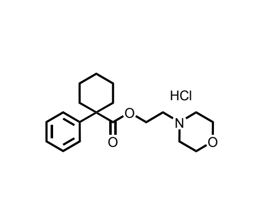	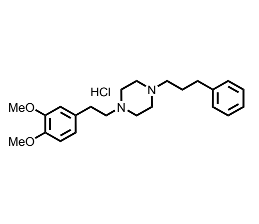	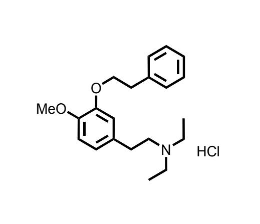	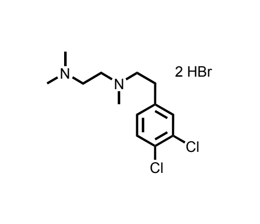	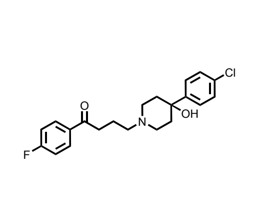	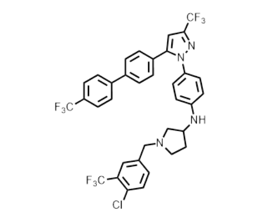
127	128	129	130	131	132	133
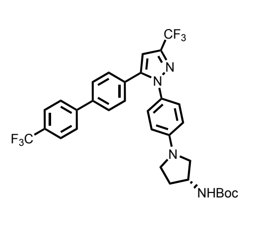	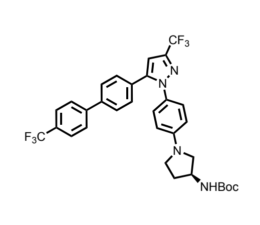	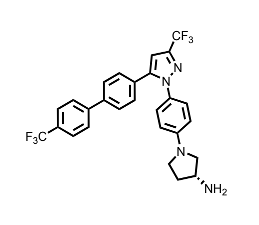	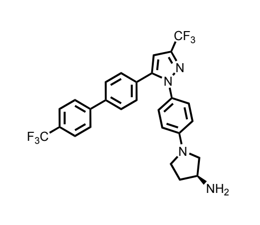	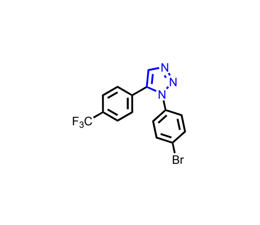	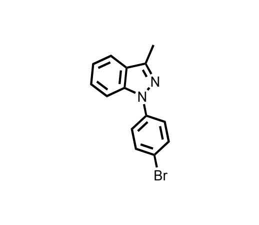	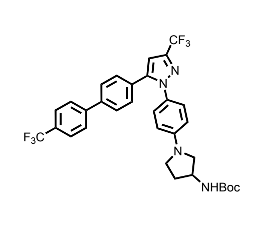
134	135	136	137	138	139	140
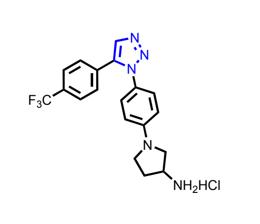	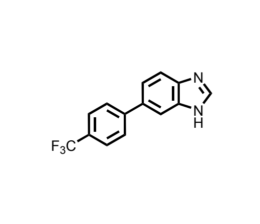	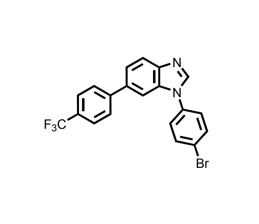	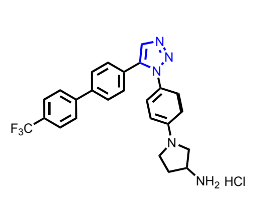	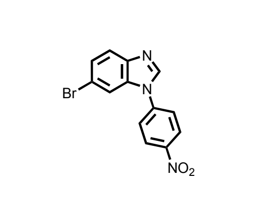	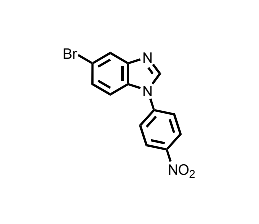	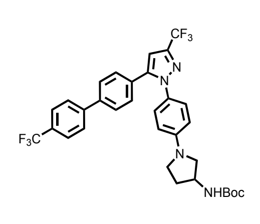

**141**	**142**	**143**	**144**	**145**	**146**	**147**
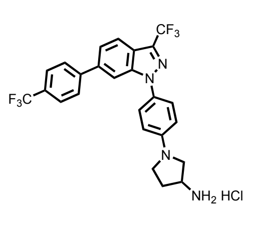	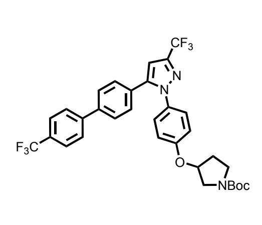	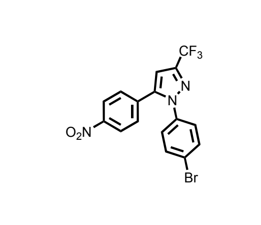	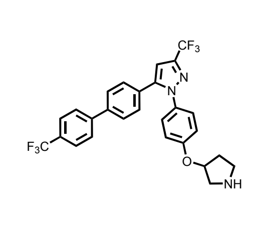	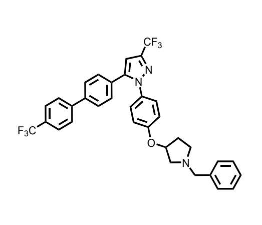	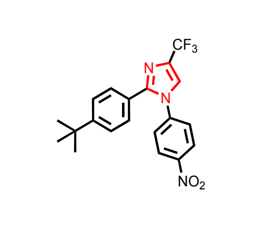	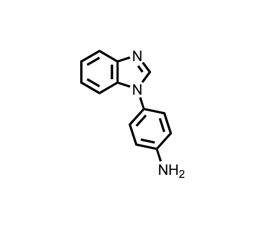
**148**	**149**	**150**	**151**	**152**	**153**	**154**
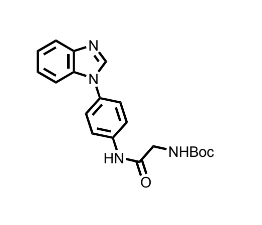	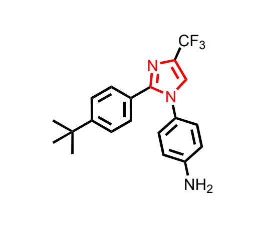	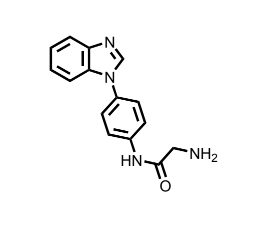	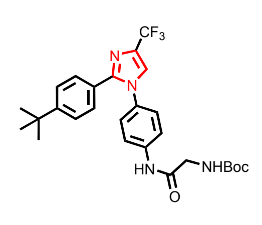	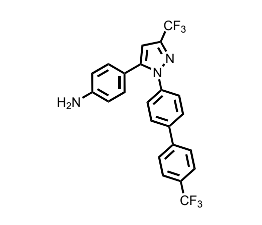	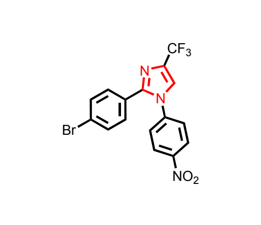	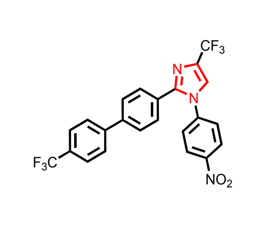
**155**	**156**	**157**	**158**	**168**	**172**	**174**
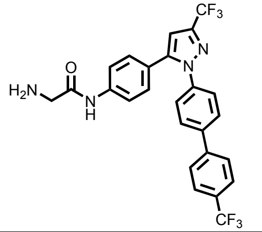	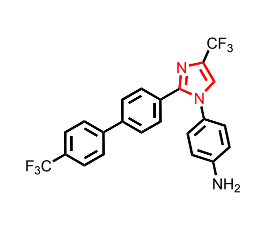	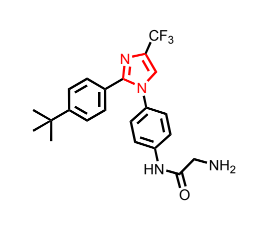	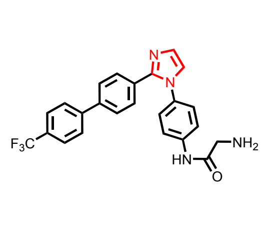	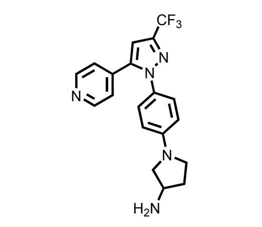	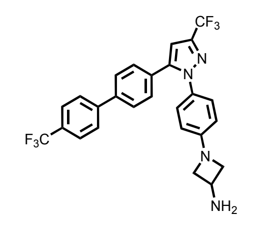	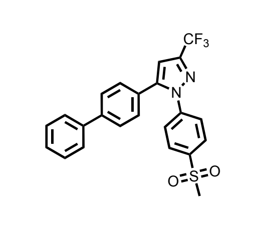
**175**	**176**	**177**	**178**	**179**	**180**	**181**
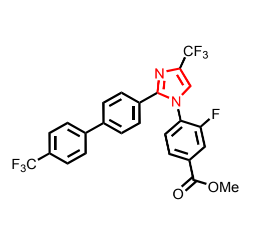	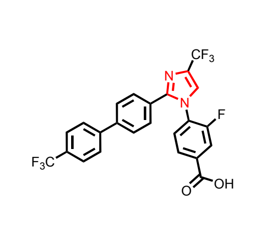	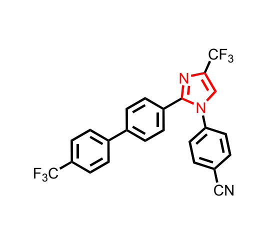	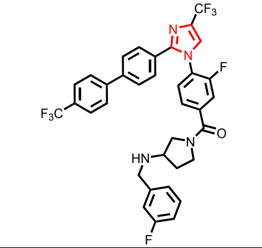	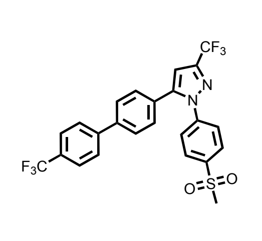	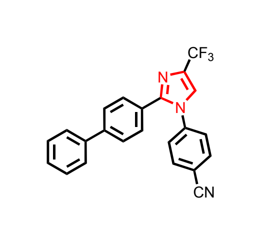	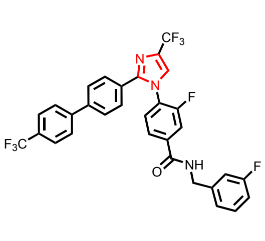
**182**	**183**	**184**	**185**	**186**	**187**	**188**
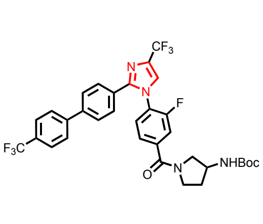	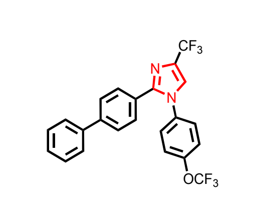	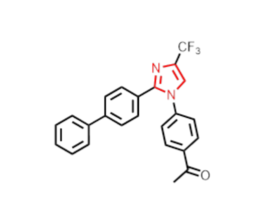	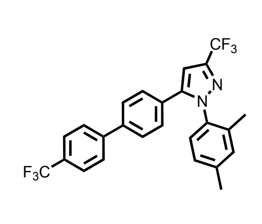	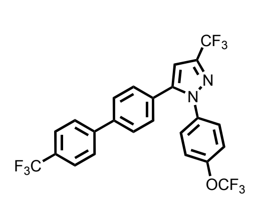	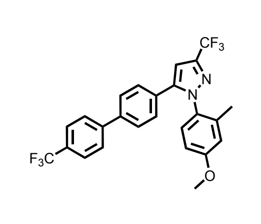	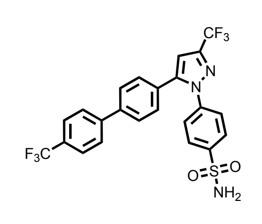

189	190	191	192	193	194	195
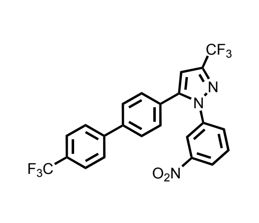	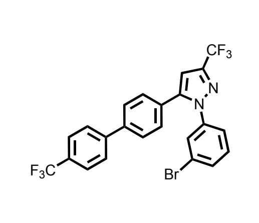	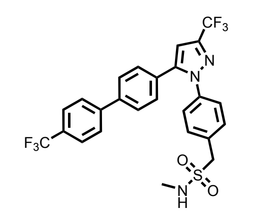	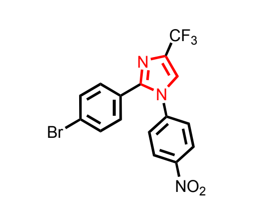	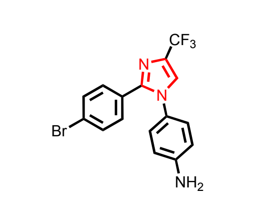	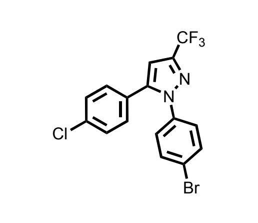	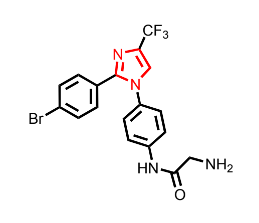
**196**	**197**	**198**	**199**	**201**	**202**	**203**
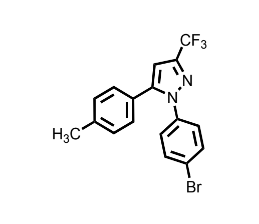	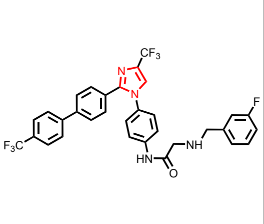	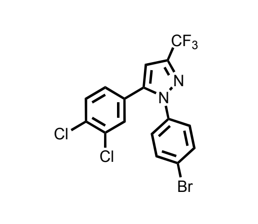	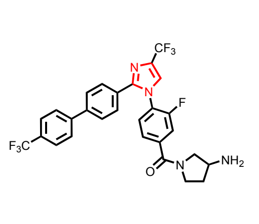	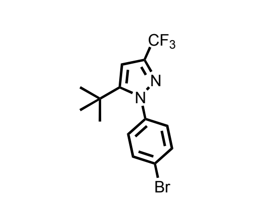	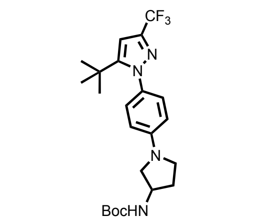	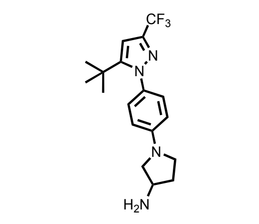
**204**	**205**	**206**	**207**	**208**	**209**	**210**
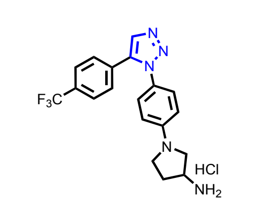	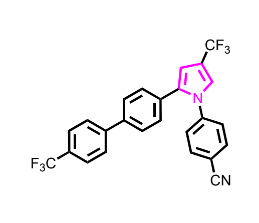	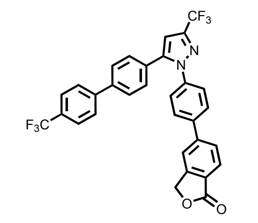	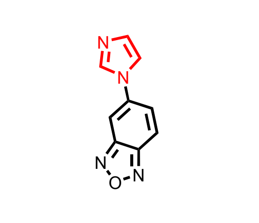	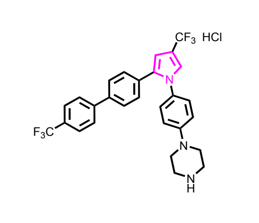	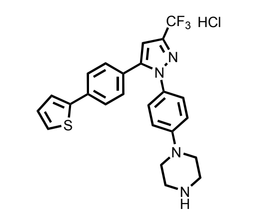	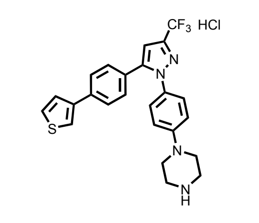
**211**	**212**	**213**	**214**	**215**	**216**	**217**
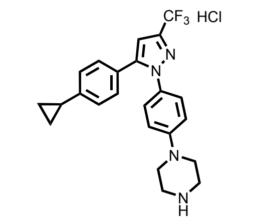	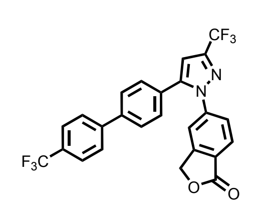	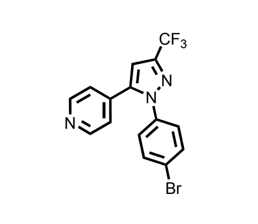	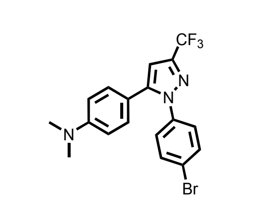	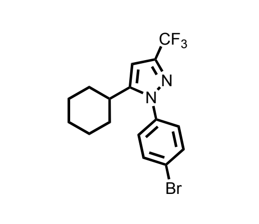	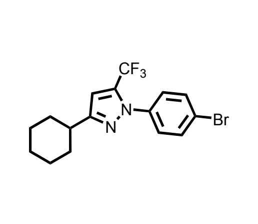	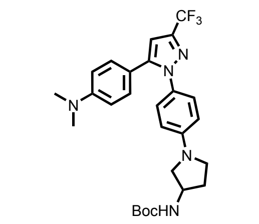
**218**	**219**	**220**	**221**	**222**	**229**	**230**
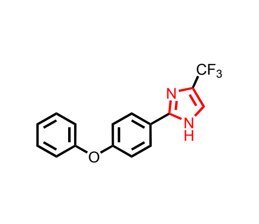	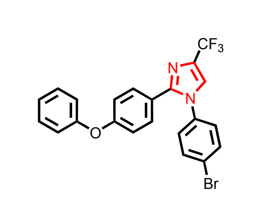	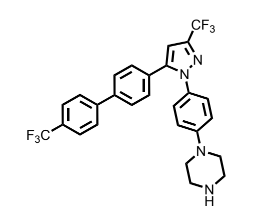	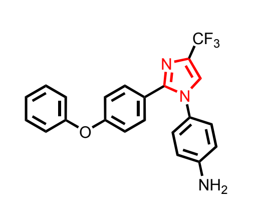	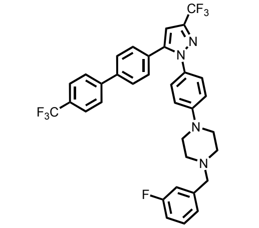	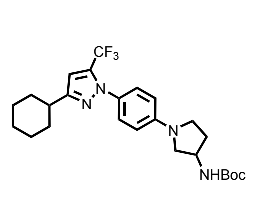	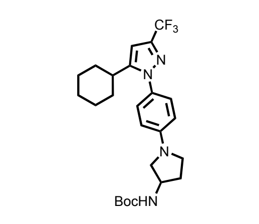

231	232	243	244	245	246	247
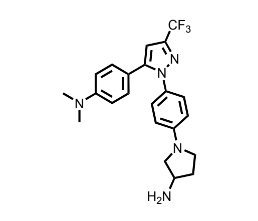	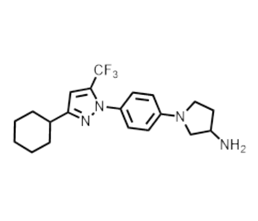	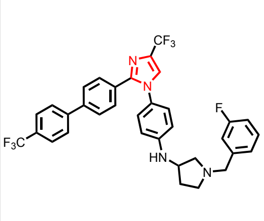	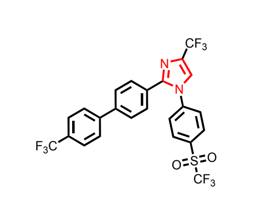	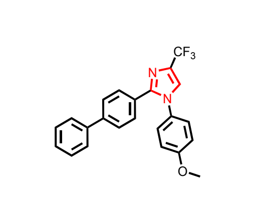	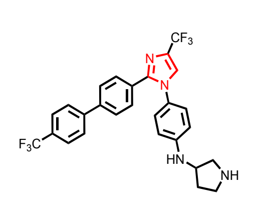	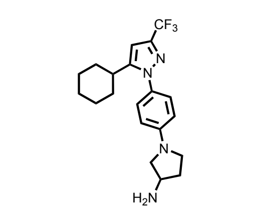
**248**	**249**	**250**	**251**	**252**	**253**	**254**
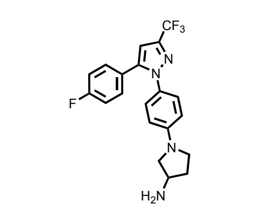	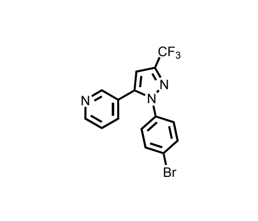	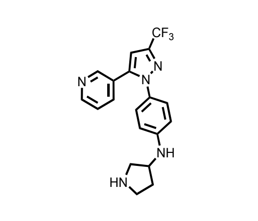	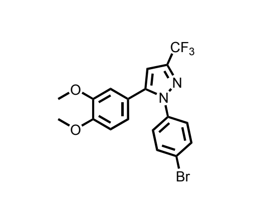	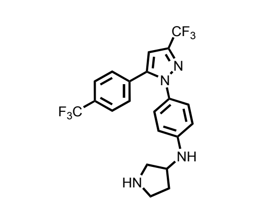	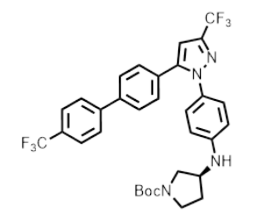	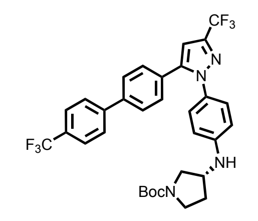
**255**	**256**	**257**	**259**	**260**	**261**	**266**
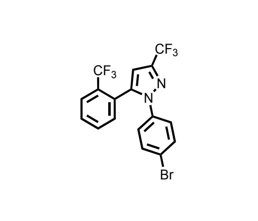	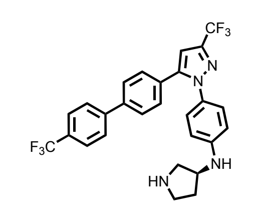	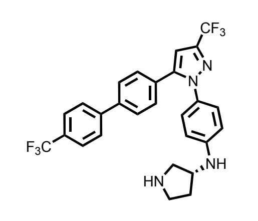	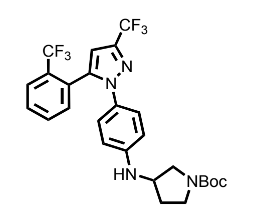	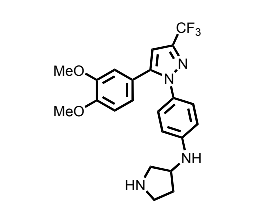	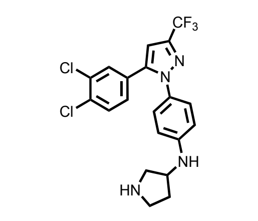	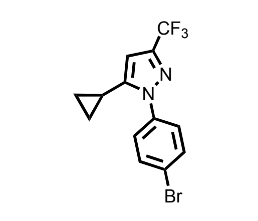
**267**	**268**	**269**	**272**	**273**	**274**	**275**
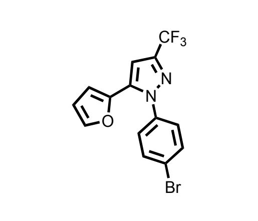	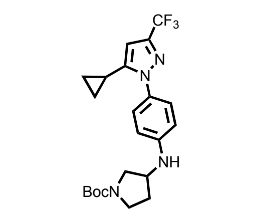	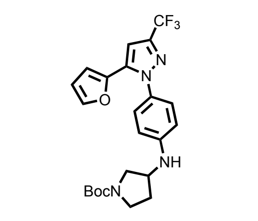	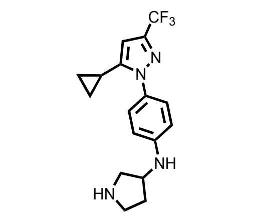	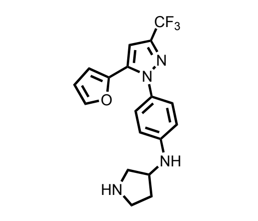	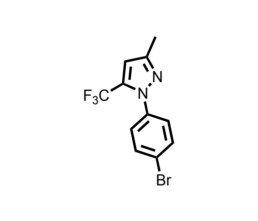	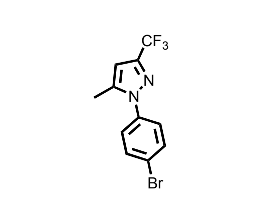
**276**	**277**	**278**	**279**	**280**	**281**	**282**
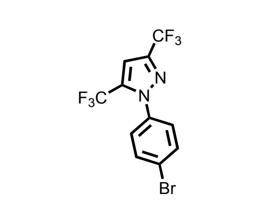	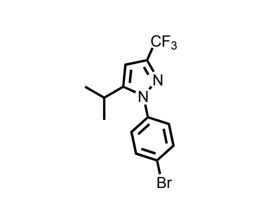	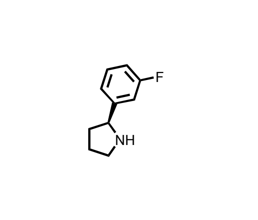	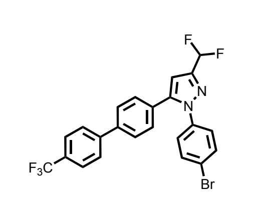	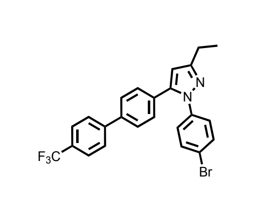	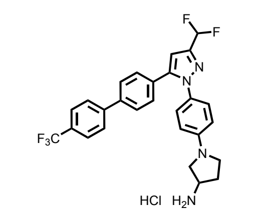	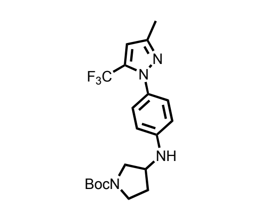

283	284	285	286	291	292	293
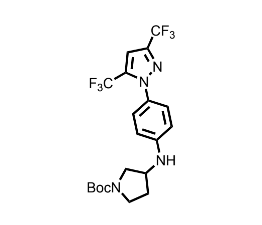	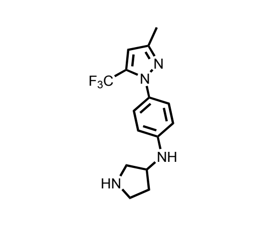	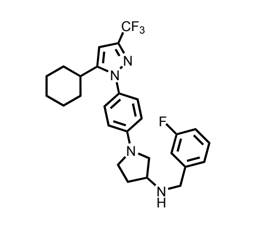	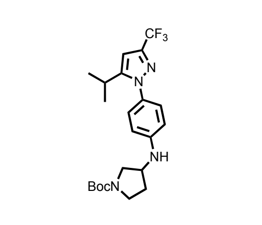	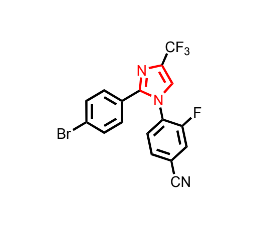	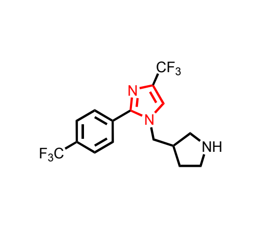	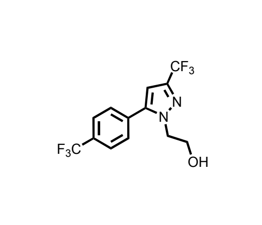
**294**	**295**	**296**	**297**	**298**	**299**	**312**
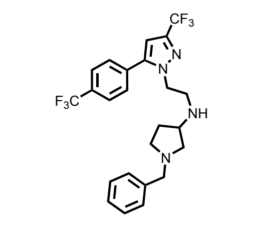	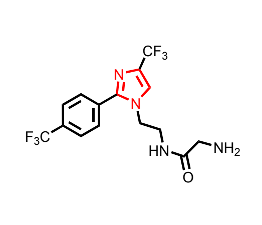	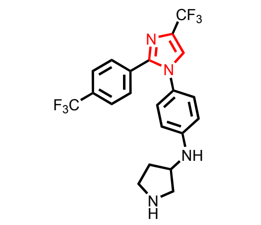	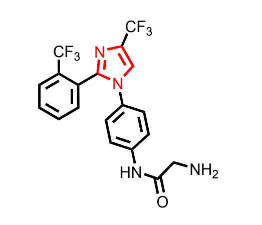	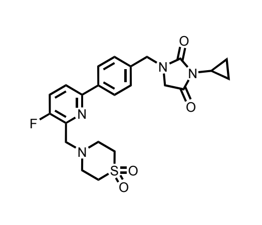	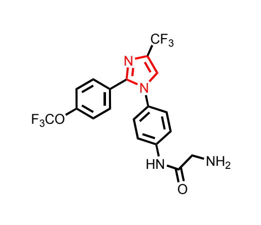	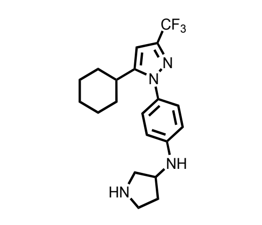
**313**	**314**	**315**	**316**	**317**	**318**	**319**
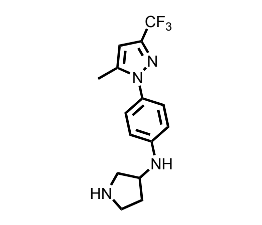	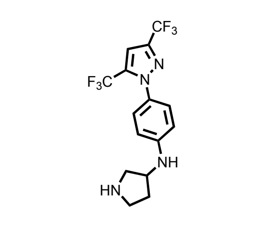	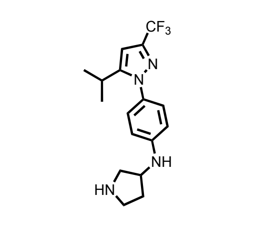	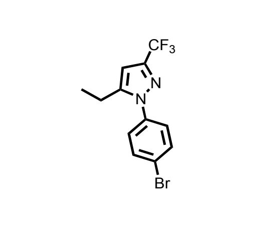	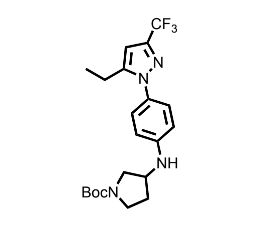	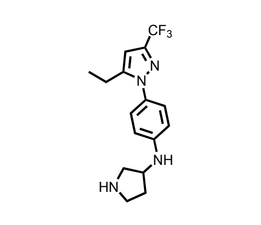	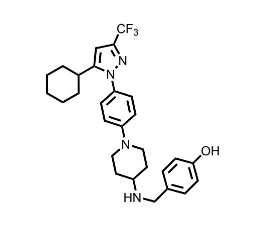
**320**	**321**	**322**	**323**	**324**	**327**	**328**
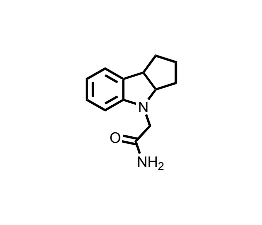	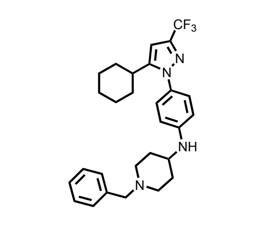	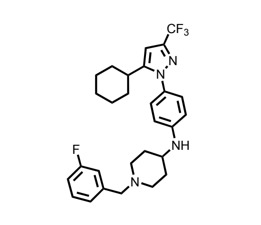	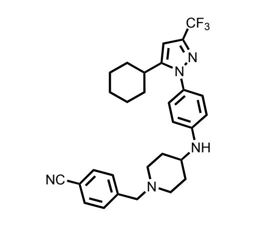	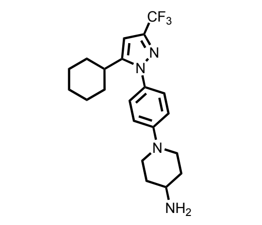	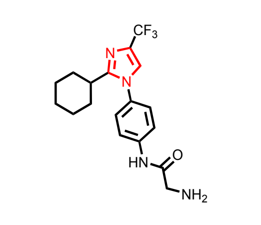	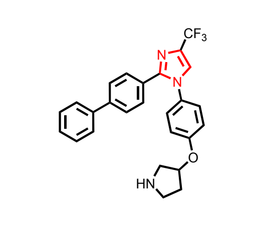
**329**	**330**	**331**	**332**	**333**	**334**	**335**
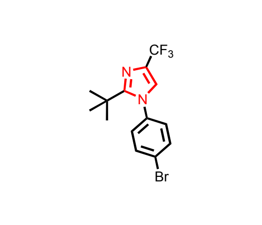	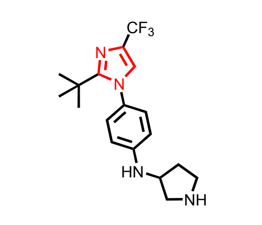	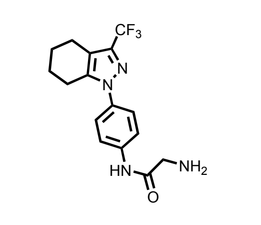	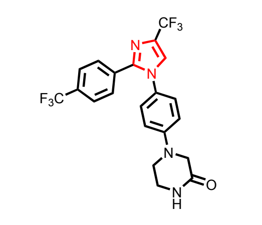	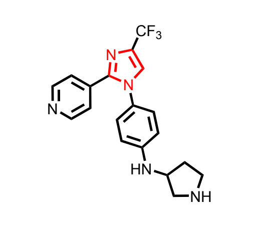	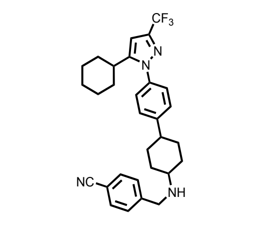	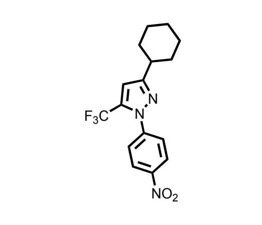

336	337	338	339	340	341	352
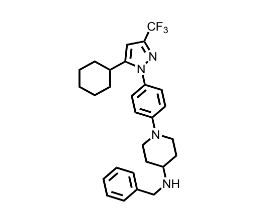	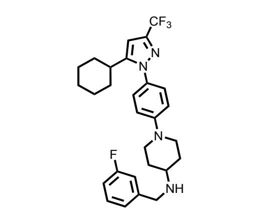	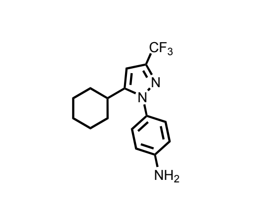	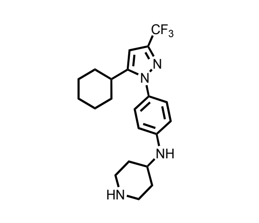	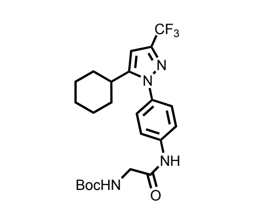	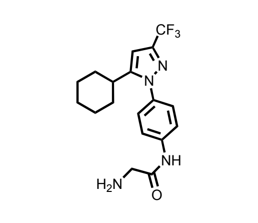	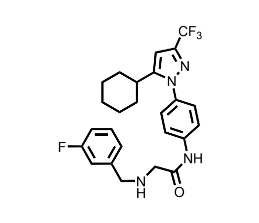
**353**	**354**	**355**	**356**	**357**	**358**	**362**
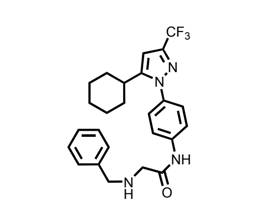	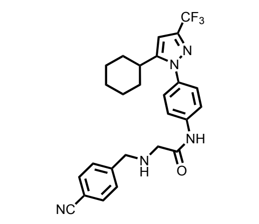	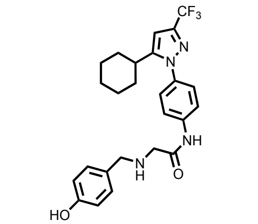	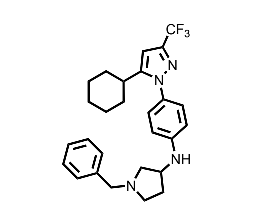	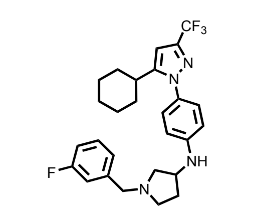	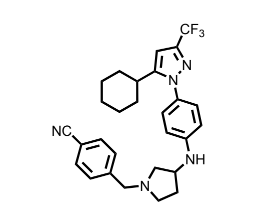	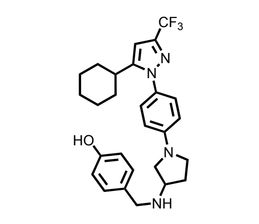
**363**	**364**	**365**	**366**	**367**	**368**	**370**
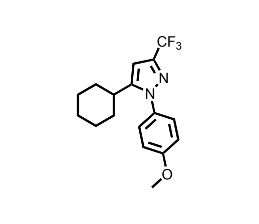	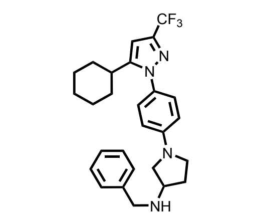	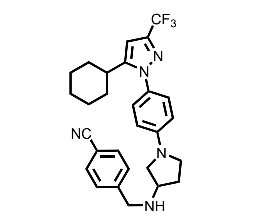	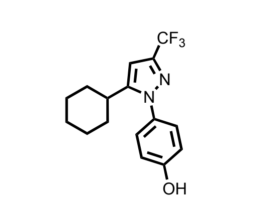	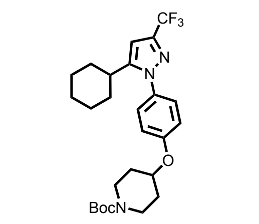	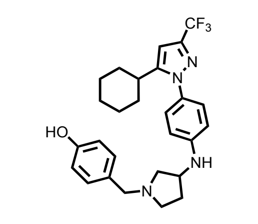	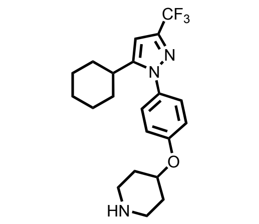
**371**	**372**	**373**	**374**	**375**	**376**	**377**
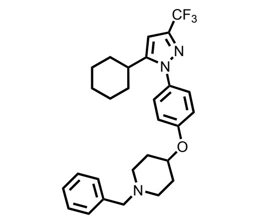	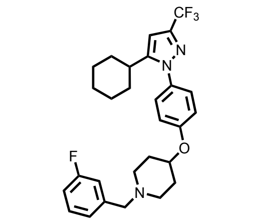	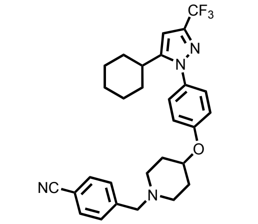	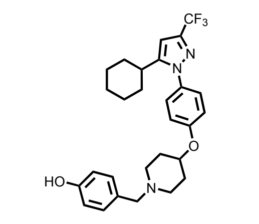	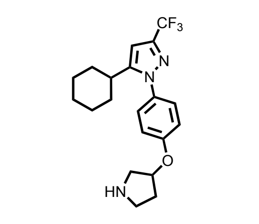	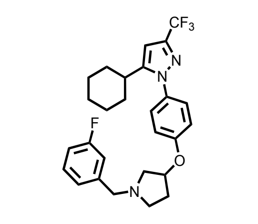	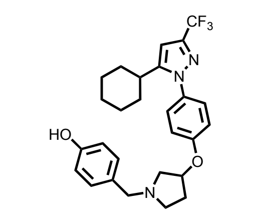
**378**	**381**	**389**	**392**	**394**	**395**	**396**
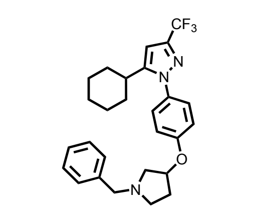	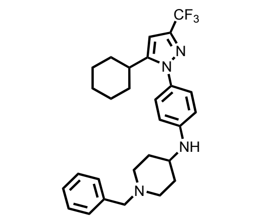	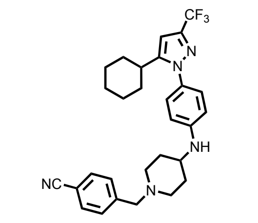	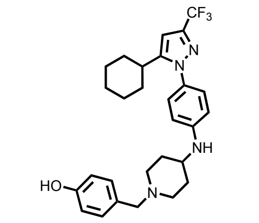	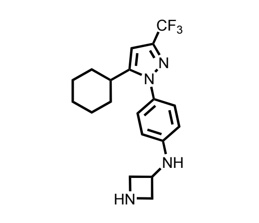	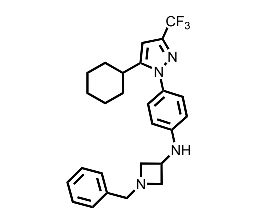	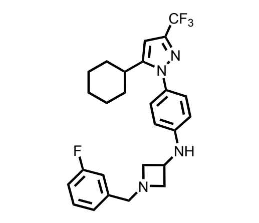

397	402	403	404	405	406	408
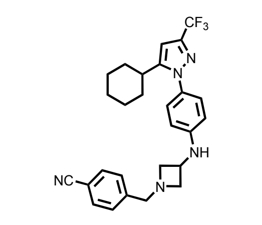	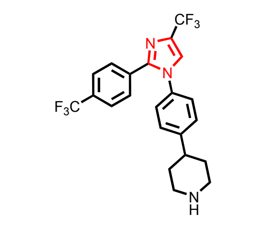	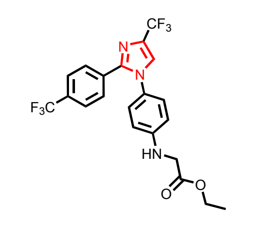	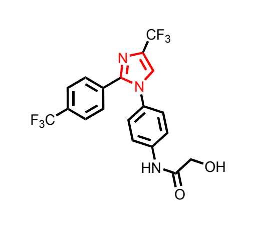	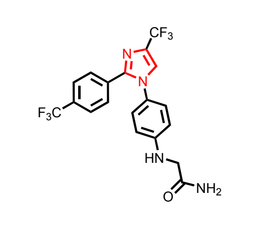	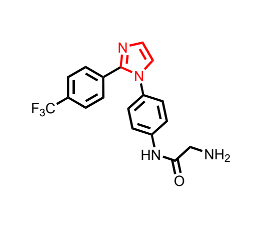	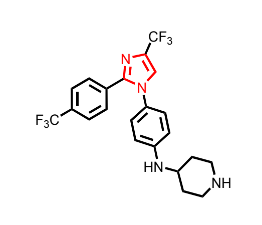
409	411	412	413	414	415	416
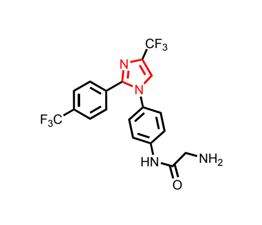	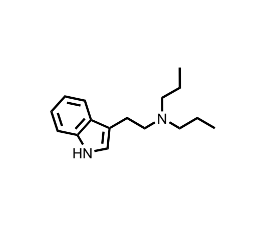	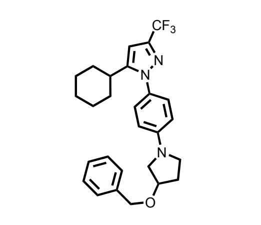	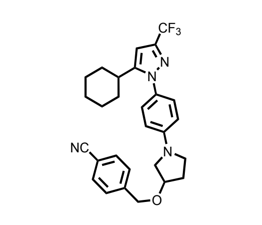	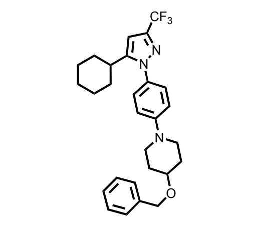	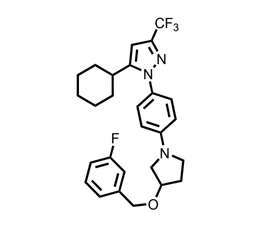	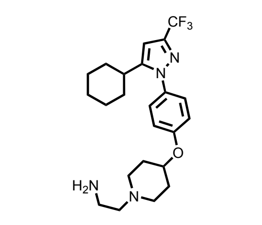
417	418	419	420	421	422	423
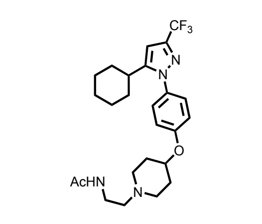	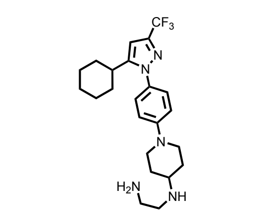	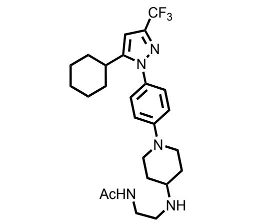	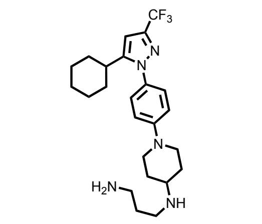	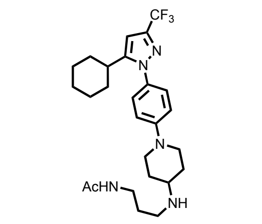	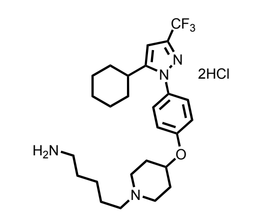	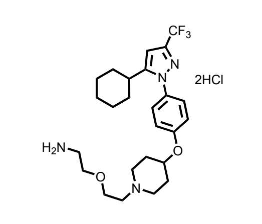
424	425	426	427	428	429	430
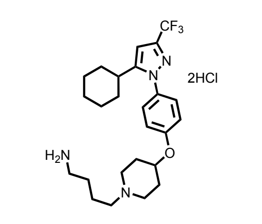	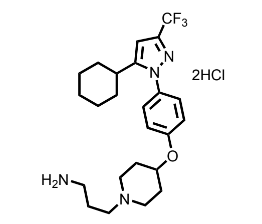	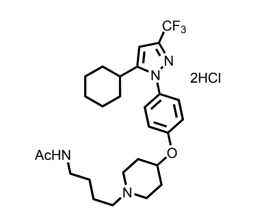	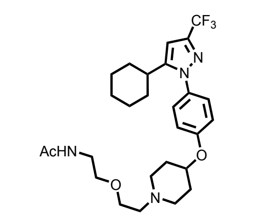	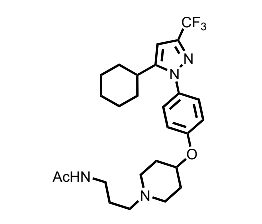	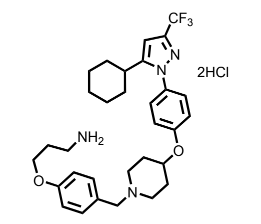	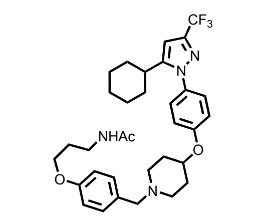
431	432	433	434	435		
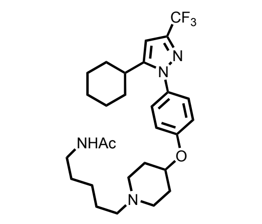	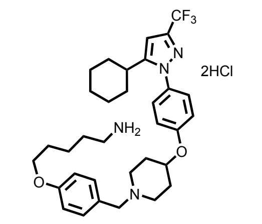	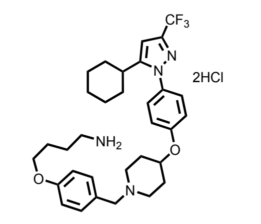	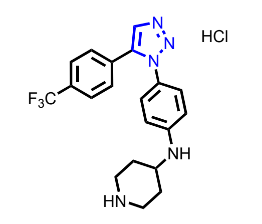	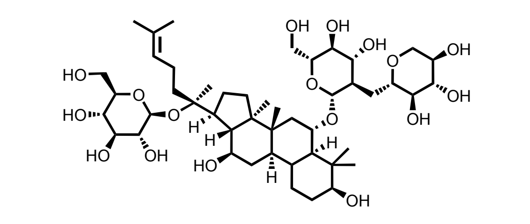		

bioRxiv preprint doi: https://doi.org/10.1101/2025.11.04.686469; this version posted November 4, 2025. The copyright holder for this preprint (which was not certified by peer review) is the author/funder, who has granted bioRxiv a license to display the preprint in perpetuity. It is made available under aCC-BY 4.0 International license.

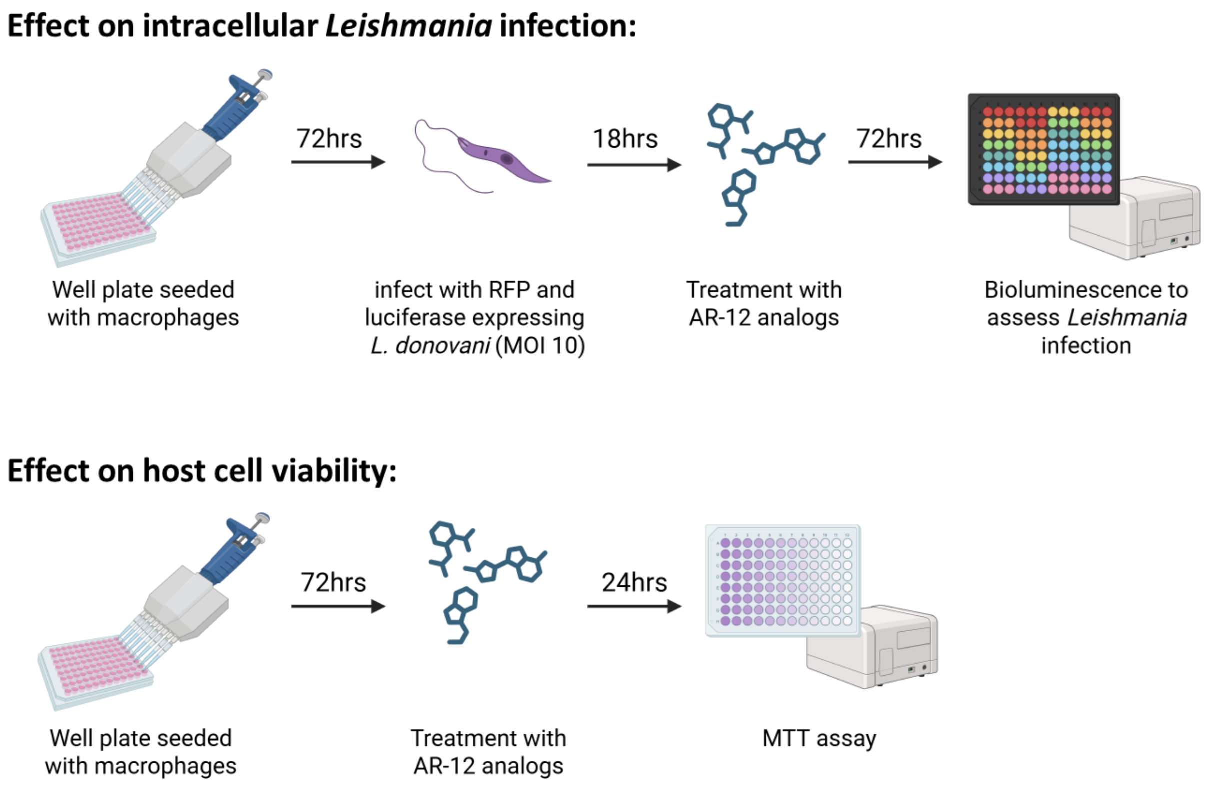

Supplemental Figure 1. Initial Screening Approach. Schematic illustrating the medium-throughput luminescence-based assay to determine effect of compounds on intracellular *Leishmania* infection and parallel screening to determine effect of compounds on host cell viability.

bioRxiv preprint doi: https://doi.org/10.1101/2025.11.04.686469; this version posted November 4, 2025. The copyright holder for this preprint (which was not certified by peer review) is the author/funder, who has granted bioRxiv a license to display the preprint in perpetuity. It is made available under aCC-BY 4.0 International license.

Supplemental Table 2. Results of primary screen in all compounds. Concentration at which intracellular *Leishmania donovani* burden is reduced by 50% in THP1 macrophages (Lum IC_{50}) as identified by luminescence assay. Concentration where THP1 macrophage cell viability is 50% (LC_{50}) after 24-hour incubation with compound as determined by MTT assay. Selectivity between host-directed effect and cytotoxicity, defined as 24h LC_{50} / Lum IC_{50}. Parental compound AR-12 provided for reference. Compounds highlighted in grey have higher selectivity than parental compound AR-12. Compounds highlighted in yellow were selected for secondary screening. ND = not determined.

CMPD	24h LC_{50} (µM)	Lum IC_{50} (µM)	Selectivity
AR-12	13.3	3.4	3.9
1	19.1	>10	<1.9
2	>50	>10	ND
3	11.0	1.8	6.1
4	>50	>10	ND
5	13.3	2.3	5.8
6	>50	>10	ND
7	5.2	2.4	2.1
8	>50	>10	ND
9	>50	>10	ND
10	>50	>10	ND
11	1.4	>10	<0.1
12	>50	>10	ND
13	>50	>10	ND
14	50.0	>10	<5.0
15	>50	>10	ND
16	>50	>10	ND
17	>50	>10	ND
18	24.5	4.0	6.1
19	>50	>10	ND
20	>50	>10	ND
21	>50	>10	ND
22	>50	-	-
23	5.8	0.4	14.8
24	>50	>10	ND
25	28.9	0.7	43.8
26	5.8	>10	<0.6
27	>50	>10	ND
28	>50	>10	ND
29	>50	>10	ND
30	>50	>10	ND
31	>50	>10	ND
32	6.3	>10	<0.6
33	1.5	>10	<0.2
34	>50	6.0	>8.3
35	>50	>10	ND
36	>50	>10	ND
37	>50	>10	ND
38	>50	>10	ND
39	>50	>10	ND
40	>50	>10	ND
41	>50	>10	ND
42	0.7	4.2	0.2
43	0.5	4.8	0.1
44	7.4	0.9	8.1
45	3.2	3.7	0.9
46	10.0	>10	<1.0
47	>50	>10	ND
48	>50	>10	ND
49	48.8	>10	<4.8
50	23.5	1.5	16.0
51	25.7	8.2	3.1
52	>50	>10	ND
53	>50	0.4	>112.6
54	9.9	2.4	4.1
55	29.7	8.5	3.5
56	>50	>10	ND
57	>50	>10	ND
58	2.6	2.2	1.2
59	>50	>10	ND
60	>50	>10	ND
61	6.1	2.3	2.7

bioRxiv preprint doi: https://doi.org/10.1101/2025.11.04.686469; this version posted November 4, 2025. The copyright holder for this preprint (which was not certified by peer review) is the author/funder, who has granted bioRxiv a license to display the preprint in perpetuity. It is made available under aCC-BY 4.0 International license.

CMPD	24h LC_{50} (µM)	Lum IC_{50} (µM)	Selectivity
62	>50	>10	ND
63	>50	>10	ND
64	>50	>10	ND
65	4.0	2.3	1.7
66	>50	>10	ND
67	>50	>10	ND
68	34.7	>10	<3.5
69	12.4	5.5	2.3
70	>50	>10	ND
71	8.4	>10	<0.8
72	>50	>10	ND
73	4.8	2.7	1.8
74	6.5	2.8	2.3
75	48.8	>10	<4.9
76	33.2	>10	<3.3
77	>50	>10	ND
78	>50	>10	ND
79	6.3	7.1	0.9
80	8.2	3.0	2.7
81	>50	>10	ND
82	>50	>10	ND
83	6.2	1.8	3.5
84	>50	6.5	>7.7
85	>50	>10	ND
86	21.9	1.4	16.0
87	>50	6.7	>7.4
88	>50	>10	ND
89	8.0	4.0	2.0
90	>50	>10	ND
91	>50	1.4	>36.1
92	14.7	>10	<1.5
93	40.0	>10	<4.0
94	29.0	>10	<2.9
95	>50	>10	ND
96	37.5	>10	<3.8
97	>50	>10	ND
98	19.5	>10	<1.9
99	29.2	>10	<2.9
100	>50	>10	ND
101	0.5	1.1	0.4

CMPD	24h LC_{50} (µM)	Lum IC_{50} (µM)	Selectivity
102	-	-	-
103	>50	>10	ND
104	>50	>10	ND
105	>50	>10	ND
106	6.0	8.1	0.7
107	>50	>10	ND
108	>50	>10	ND
109	9.4	3.3	2.8
110	8.5	4.0	2.1
111	5.2	2.9	1.8
112	6.3	>10	<0.6
113	19.0	>10	<1.9
114	13.6	>10	<1.4
115	13.2	7.4	1.8
116	>50	9.9	>5.0
117	>50	6.1	>8.2
118	6.2	2.6	2.4
119	37.2	>10	<3.7
120	>50	>10	ND
126	>50	>10	ND
127	>50	>10	ND
128	>50	>10	ND
129	6.3	0.1	46.7
130	4.2	0.2	23.9
131	37.2	>10	<3.7
132	>50	>10	ND
133	29.0	0.9	32.5
134	22.6	0.5	42.1
135	>50	>10	ND
136	>50	>10	ND
137	7.3	4.4	1.7
138	>50	>10	ND
139	>50	>10	ND
140	>50	>10	ND
141	7.7	3.6	2.1
142	>50	>10	ND
143	>50	>10	ND
144	5.4	>10	<0.5
145	>50	>10	ND
146	>50	4.4	>11.4

bioRxiv preprint doi: https://doi.org/10.1101/2025.11.04.686469; this version posted November 4, 2025. The copyright holder for this preprint (which was not certified by peer review) is the author/funder, who has granted bioRxiv a license to display the preprint in perpetuity. It is made available under aCC-BY 4.0 International license.

CMPD	24h LC_{50} (µM)	Lum IC_{50} (µM)	Selectivity
147	>50	>10	ND
148	>50	>10	ND
149	28.8	9.1	3.2
150	>50	>10	ND
151	31.8	>10	<3.2
152	43.6	>10	<4.4
153	>50	>10	ND
154	25.5	2.9	8.9
155	6.1	4.0	1.5
156	20.8	5.8	3.6
157	27.6	>10	<2.8
158	16.0	0.2	81.0
168	>50	10.0	>5.0
172	>50	>10	ND
174	>50	>10	ND
175	>50	>10	ND
176	>50	>10	ND
177	>50	>10	ND
178	>50	>10	ND
179	45.0	>10	<4.5
180	>50	>10	ND
181	28.7	>10	<2.9
182	18.0	5.9	3.0
183	>50	>10	ND
184	>50	5.0	>10
185	>50	>10	ND
186	>50	>10	ND
187	>50	>10	ND
188	9.0	3.1	2.9
189	>50	>10	ND
190	>50	>10	ND
191	12.0	>10	<1.2
192	>50	>10	ND
193	>50	>10	ND
194	>50	>10	ND
195	38.6	>10	<3.9
196	>50	>10	ND
197	>50	2.4	>20.6
198	>50	>10	ND
199	9.4	1.3	7.2

CMPD	24h LC_{50} (µM)	Lum IC_{50} (µM)	Selectivity
201	>50	>10	ND
202	>50	8.5	>5.9
203	11.8	2.9	4.1
204	37.2	>10	<3.7
205	>50	>10	ND
206	>50	>10	ND
207	>50	>10	ND
208	31.0	5.7	5.4
209	10.2	3.0	3.4
210	10.5	2.5	4.3
211	13.2	2.1	6.3
212	>50	>10	ND
213	>50	>10	ND
214	>50	>10	ND
215	>50	>10	ND
216	>50	>10	ND
217	>50	>10	ND
218	>50	>10	ND
219	>50	>10	ND
220	15.4	1.3	11.8
221	>50	>10	ND
222	>50	>10	ND
229	5.0	3.4	1.5
230	9.5	>10	<1.0
231	10.8	1.8	6.2
232	14.4	2.6	5.6
243	>50	>10	ND
244	>50	>10	ND
245	>50	>10	ND
246	5.5	3.8	1.4
247	7.6	3.3	2.3
248	14.4	2.6	5.6
249	50.0	>10	<5.0
250	>50	>10	ND
251	>50	>10	ND
252	14.4	2.3	6.2
253	>50	>10	ND
254	50.0	>10	<5.0
255	>50	>10	ND
256	6.2	2.5	2.5

bioRxiv preprint doi: https://doi.org/10.1101/2025.11.04.686469; this version posted November 4, 2025. The copyright holder for this preprint (which was not certified by peer review) is the author/funder, who has granted bioRxiv a license to display the preprint in perpetuity. It is made available under aCC-BY 4.0 International license.

CMPD	24h LC_{50} (µM)	Lum IC_{50} (µM)	Selectivity
257	5.0	2.5	2.0
259	23.6	>10	<2.4
260	13.9	4.3	3.2
261	22.4	3.1	7.3
266	>50	>10	ND
267	>50	>10	ND
268	>50	>10	ND
269	>50	>10	ND
272	17.2	5.6	3.1
273	15.6	4.9	3.2
274	>50	>10	ND
275	>50	>10	ND
276	32.2	>10	<0.6
277	>50	>10	ND
278	>50	>10	ND
279	>50	>10	ND
280	>50	>10	ND
281	14.3	2.0	7.2
282	>50	>10	ND
283	>50	>10	ND
284	>50	>10	ND
285	>50	>10	ND
286	>50	>10	ND
291	>50	>10	ND
292	>50	>10	ND
293	>50	>10	ND
294	28.2	>10	<2.8
295	>50	>10	ND
296	12.9	3.0	4.3
297	>50	>10	ND
298	>50	>10	ND
299	31.6	>10	<3.2
312	25.5	4.8	5.3
313	>50	>10	ND
314	21.9	2.7	8.0
315	25.1	3.3	7.6
316	>50	>10	ND
317	>50	>10	ND
318	27.0	1.8	14.8
319	6.4	0.8	8.1

CMPD	24h LC_{50} (µM)	Lum IC_{50} (µM)	Selectivity
320	>50	>10	ND
321	23.9	5.1	4.7
322	>50	>10	ND
323	28.3	8.9	3.2
324	27.5	1.9	14.2
327	>50	>10	ND
328	11.1	2.6	4.3
329	>50	>10	ND
330	>50	6.2	>8.1
331	47.0	9.9	4.7
332	31.2	8.0	3.9
333	>50	>10	ND
334	24.5	2.5	9.7
335	>50	>10	ND
336	15.9	2.4	6.6
337	32.1	3.5	9.3
338	>50	>10	ND
339	18.2	1.7	10.4
340	>50	>10	ND
341	26.5	4.0	6.6
352	29.3	>10	<2.9
353	17.5	4.2	4.2
354	22.0	0.5	44.9
355	21.2	2.0	10.5
356	15.5	2.0	7.6
357	13.1	1.2	10.7
358	13.2	>10	<1.3
362	20.0	2.2	9.0
363	>50	>10	ND
364	31.1	6.8	4.5
365	32.9	>10	<3.1
366	30.2	>10	<3.3
367	>50	>10	ND
368	12.1	1.6	7.6
370	19.5	2.6	7.5
371	>50	8.9	>5.6
372	>50	>10	ND
373	>50	7.0	>7.2
374	8.2	3.1	2.6
375	12.3	6.3	1.9

bioRxiv preprint doi: https://doi.org/10.1101/2025.11.04.686469; this version posted November 4, 2025. The copyright holder for this preprint (which was not certified by peer review) is the author/funder, who has granted bioRxiv a license to display the preprint in perpetuity. It is made available under aCC-BY 4.0 International license.

CMPD	24h LC_{50} (µM)	Lum IC_{50} (µM)	Selectivity
376	>50	>10	ND
377	14.6	8.3	1.8
378	>50	>10	ND
381	12.4	6.2	2.0
389	>50	>10	ND
392	25.9	4.7	5.5
394	30.9	5.5	5.6
395	37.4	7.6	4.9
396	23.3	6.1	3.8
397	27.6	8.1	3.4
402	26.9	>10	<3.0
403	>50	>10	ND
404	>50	>10	ND
405	>50	>10	ND
406	>50	>10	ND
407	>50	>10	ND
408	25.4	1.5	17.0
409	35.2	8.8	4.0
411	>50	>10	ND
412	>50	>10	ND
413	>50	>10	ND
414	>50	>10	ND

CMPD	24h LC_{50} (µM)	Lum IC_{50} (µM)	Selectivity
415	>50	>10	ND
416	18.2	1.3	14.2
417	24.4	3.0	8.1
418	12.6	3.0	4.2
419	21.6	2.7	7.9
420	7.0	1.0	6.7
421	19.0	1.5	12.5
422	6.1	2.5	2.4
423	3.4	6.0	0.6
424	6.5	2.4	2.7
425	5.5	2.2	2.5
426	31.9	4.4	7.3
427	29.5	6.1	4.8
428	11.4	8.9	1.3
429	12.1	2.4	5.1
430	16.5	>10	<1.6
431	22.4	4.8	4.7
432	5.6	2.9	2.0
433	27.0	3.8	7.1
434	24.6	>10	<2.5
435	26.9	>10	<2.7

bioRxiv preprint doi: https://doi.org/10.1101/2025.11.04.686469; this version posted November 4, 2025. The copyright holder for this preprint (which was not certified by peer review) is the author/funder, who has granted bioRxiv a license to display the preprint in perpetuity. It is made available under aCC-BY 4.0 International license.

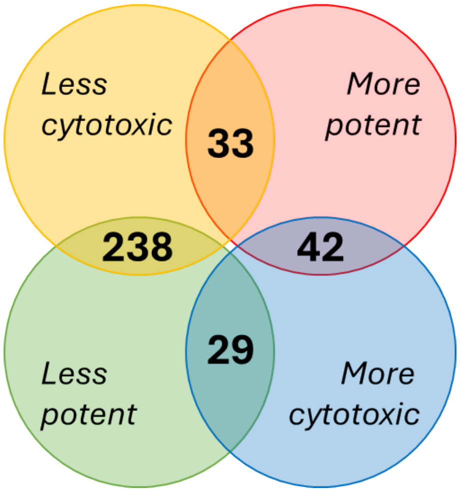

Supplemental Figure 2. Venn diagram demonstrating compound potency against *intracellular L. donovani* (Lum IC_{50}) and cytotoxicity against THP-1 host cell (24 hr LC_{50}) relative to parental compound AR-12.

bioRxiv preprint doi: https://doi.org/10.1101/2025.11.04.686469; this version posted November 4, 2025. The copyright holder for this preprint (which was not certified by peer review) is the author/funder, who has granted bioRxiv a license to display the preprint in perpetuity. It is made available under aCC-BY 4.0 International license.

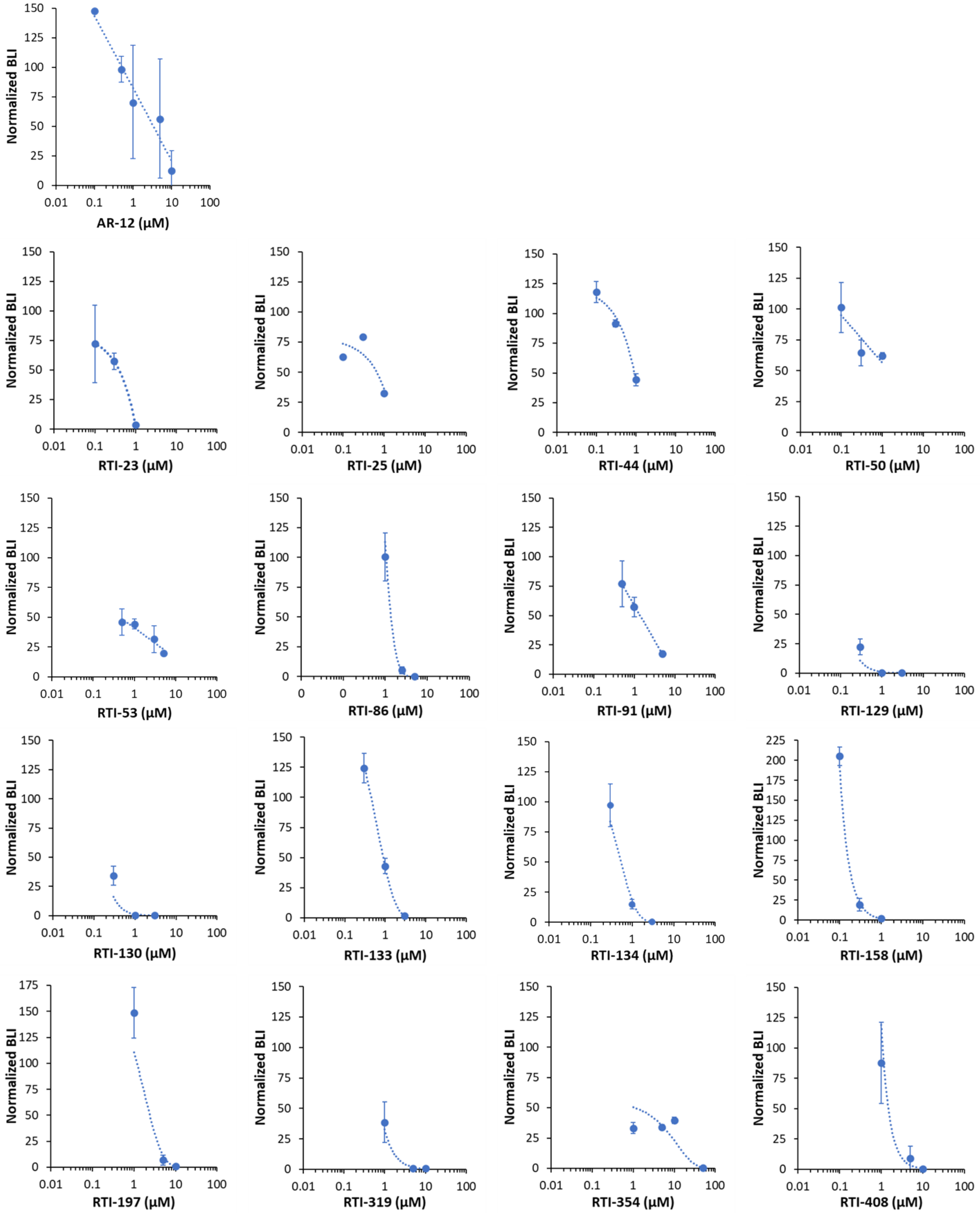

Supplemental Figure 3. Luminescent activity of intracellular *L. donovani* infected THP1 macrophage cell after 72-hour incubation with compounds.

bioRxiv preprint doi: https://doi.org/10.1101/2025.11.04.686469; this version posted November 4, 2025. The copyright holder for this preprint (which was not certified by peer review) is the author/funder, who has granted bioRxiv a license to display the preprint in perpetuity. It is made available under aCC-BY 4.0 International license.

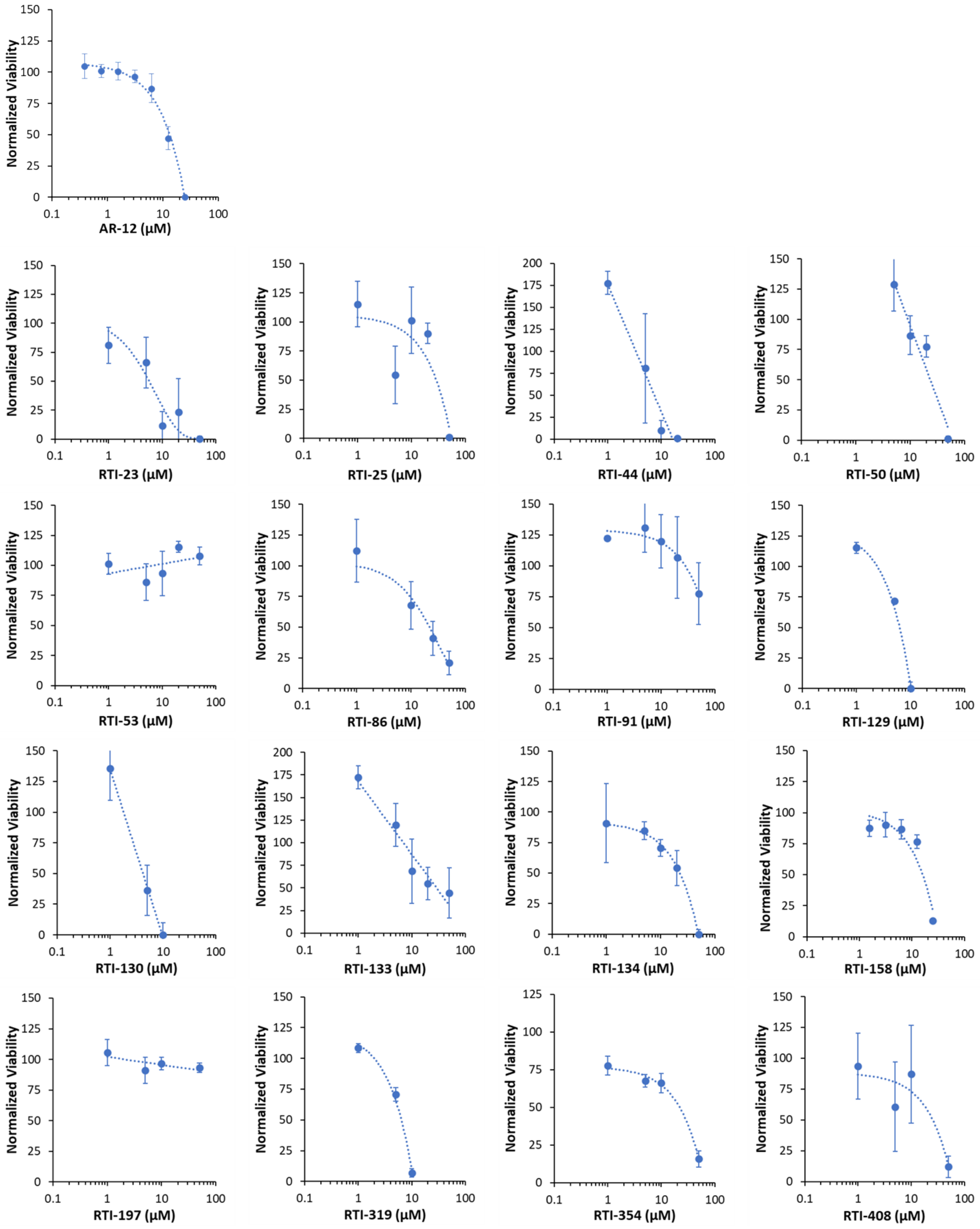

Supplemental Figure 4. Graphs of THP1 macrophage cell viability after 24-hour incubation with compounds as determined by MTT assay.

bioRxiv preprint doi: https://doi.org/10.1101/2025.11.04.686469; this version posted November 4, 2025. The copyright holder for this preprint (which was not certified by peer review) is the author/funder, who has granted bioRxiv a license to display the preprint in perpetuity. It is made available under aCC-BY 4.0 International license.

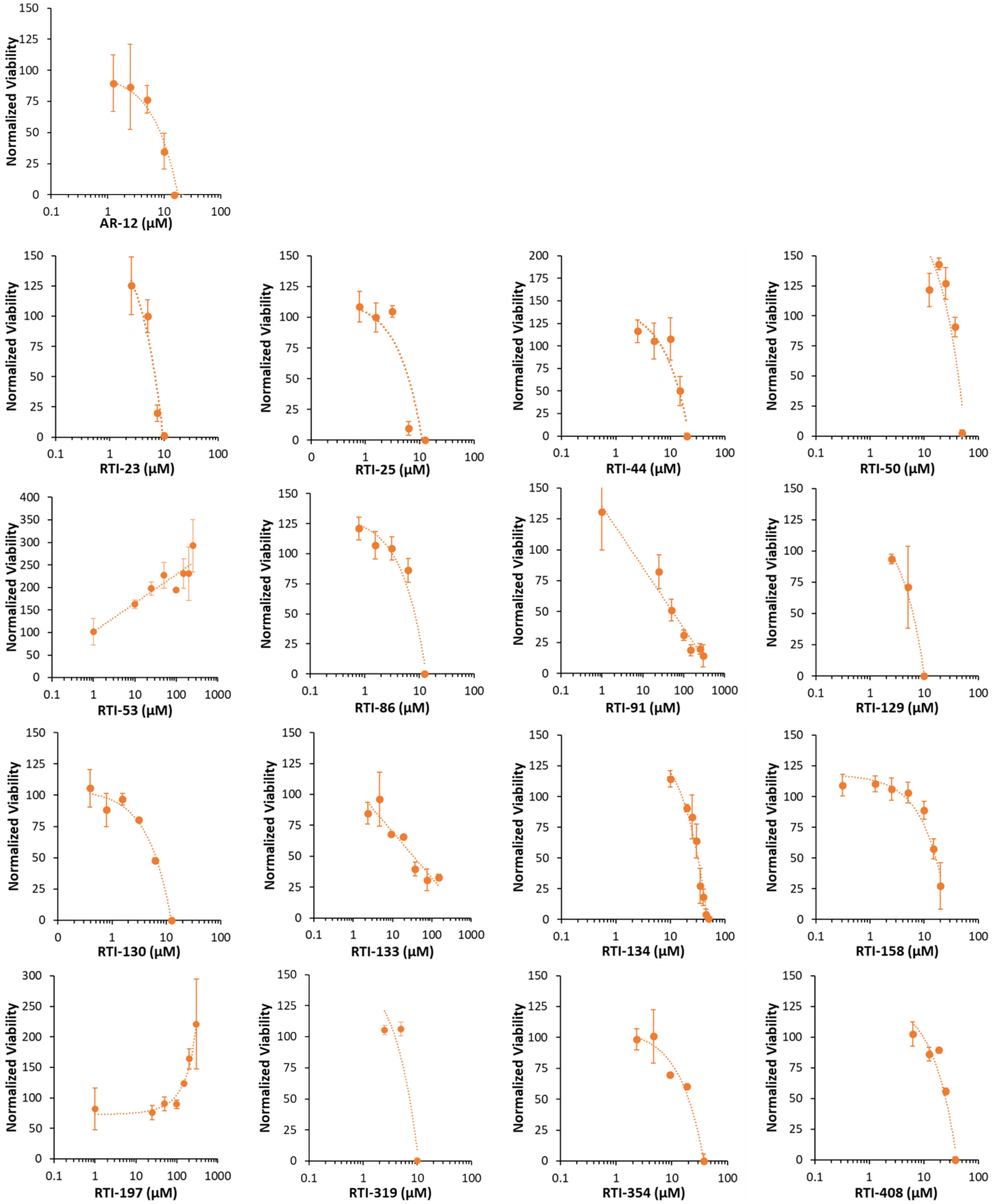

Supplemental Figure 5. Graphs of THP1 macrophage cell viability after 72-hour incubation with compounds as determined by MTT assay.

bioRxiv preprint doi: https://doi.org/10.1101/2025.11.04.686469; this version posted November 4, 2025. The copyright holder for this preprint (which was not certified by peer review) is the author/funder, who has granted bioRxiv a license to display the preprint in perpetuity. It is made available under aCC-BY 4.0 International license.

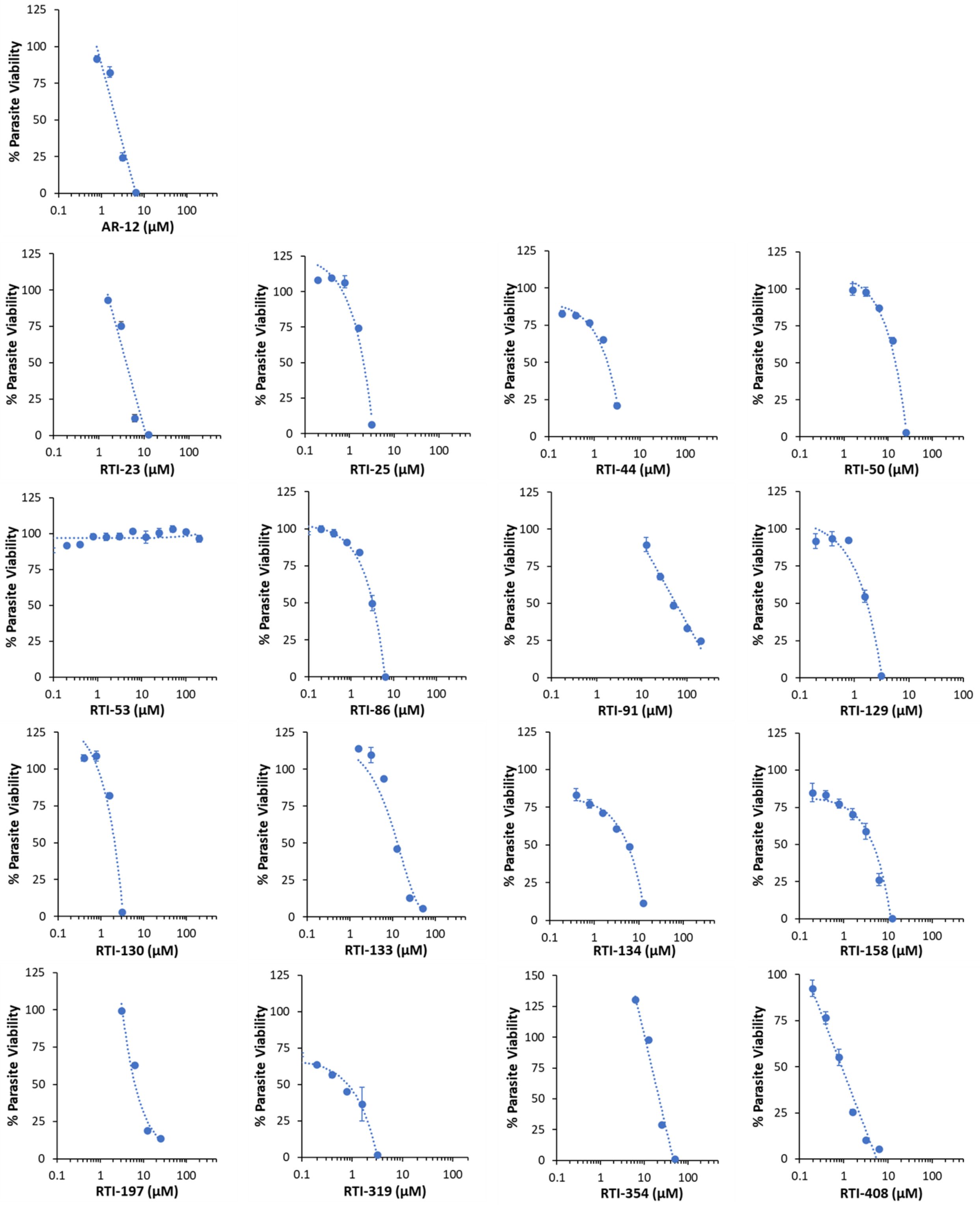

Supplemental Figure 6. Dose response of extracellular *Leishmania* promastigote viability after 72-hour incubation with compound as measured by resazurin assay.

bioRxiv preprint doi: https://doi.org/10.1101/2025.11.04.686469; this version posted November 4, 2025. The copyright holder for this preprint (which was not certified by peer review) is the author/funder, who has granted bioRxiv a license to display the preprint in perpetuity. It is made available under aCC-BY 4.0 International license.

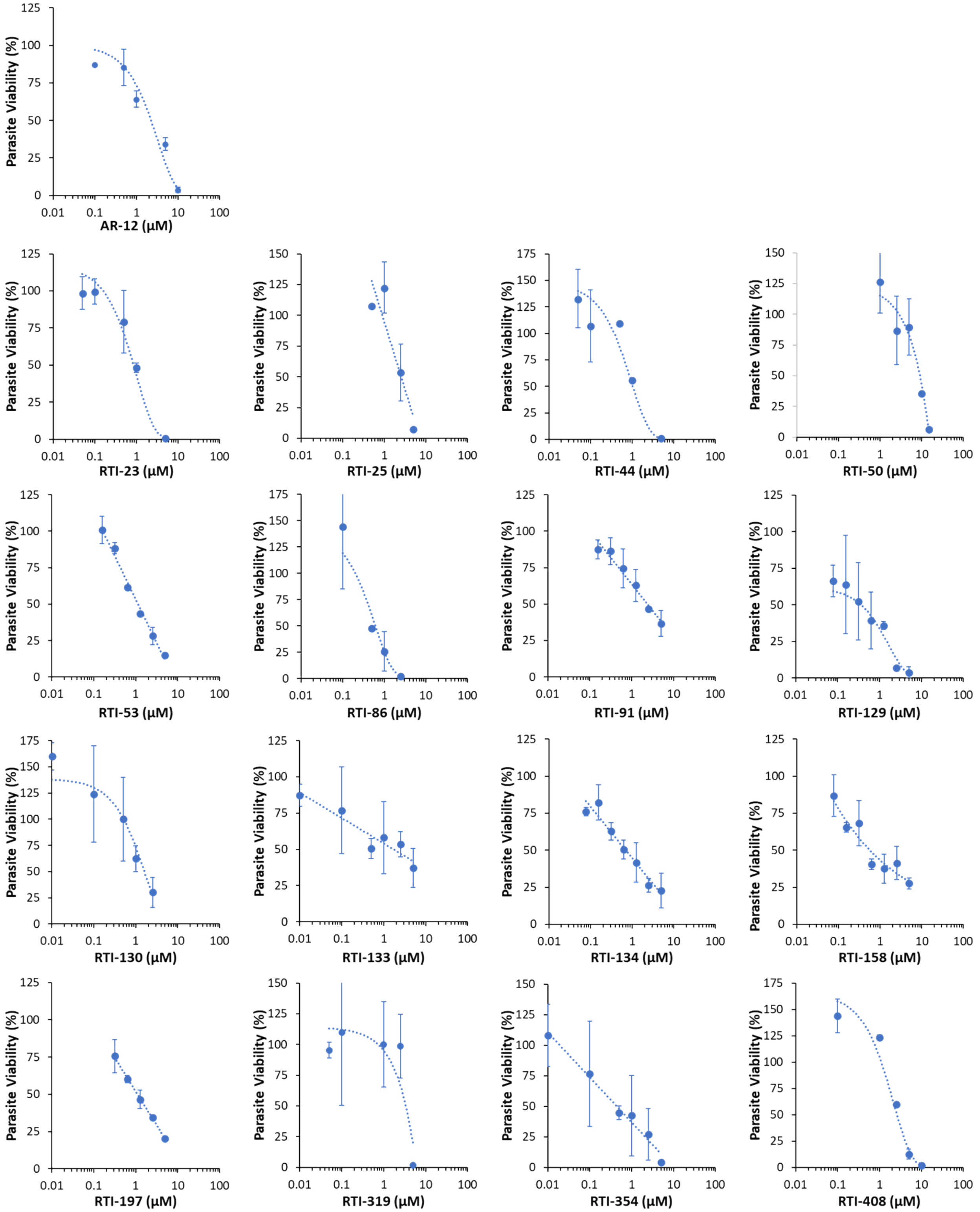

Supplemental Figure 7. Dose response of intracellular *Leishmania donovani* burden in bone marrow derived macrophages after 72-hour incubation with compound as identified image-based giemsa staining.

bioRxiv preprint doi: https://doi.org/10.1101/2025.11.04.686469; this version posted November 4, 2025. The copyright holder for this preprint (which was not certified by peer review) is the author/funder, who has granted bioRxiv a license to display the preprint in perpetuity. It is made available under aCC-BY 4.0 International license.

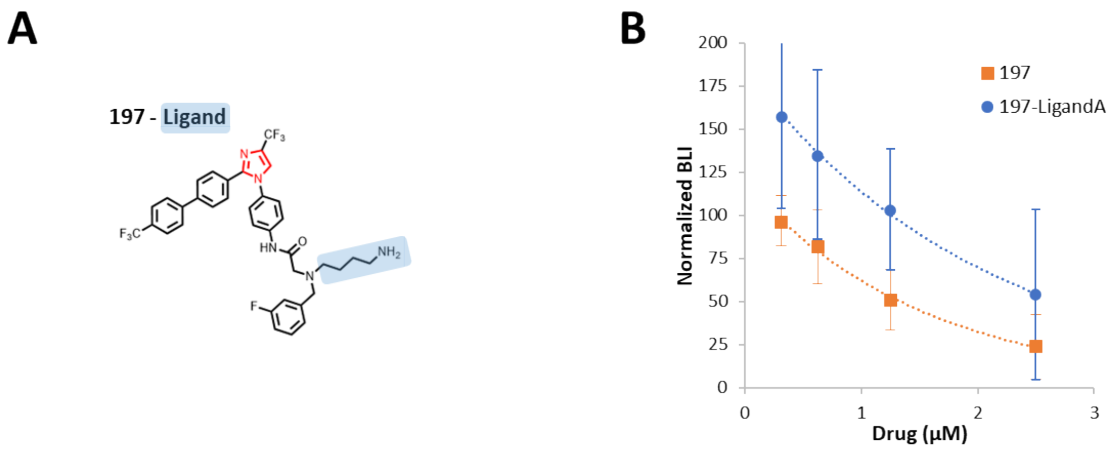

Supplemental Figure 8. A) Chemical structure of 197 chemically modified for conjugation to agarose bead for affinity capture proteomic analysis. **B)** Luminescent activity of intracellular *L. donovani* infected THP1 macrophage cell after 72-hour incubation with 197 compared to chemically modified 197.

bioRxiv preprint doi: https://doi.org/10.1101/2025.11.04.686469; this version posted November 4, 2025. The copyright holder for this preprint (which was not certified by peer review) is the author/funder, who has granted bioRxiv a license to display the preprint in perpetuity. It is made available under aCC-BY 4.0 International license.

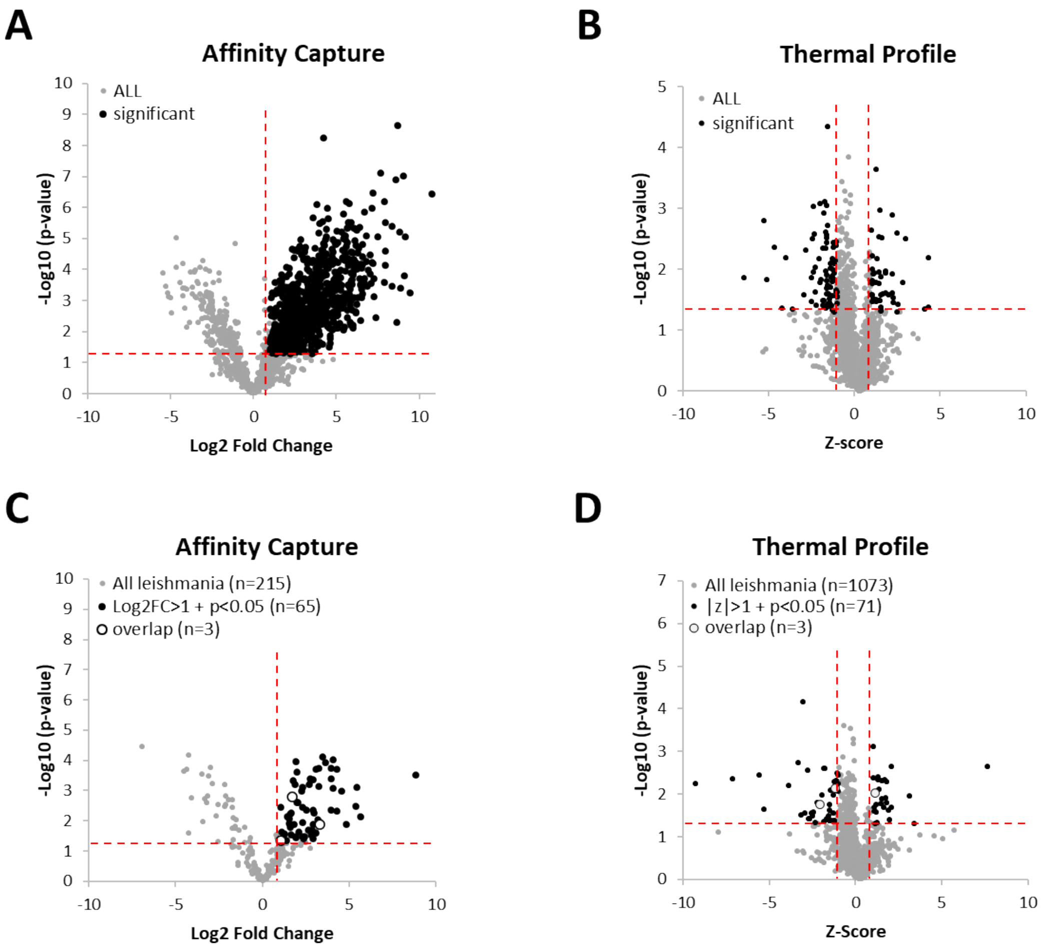

E

Accession	Description	Affinity Log2FC	Affinity p-value	Thermal Profile z-score	Thermal Profile p-value
E9AJC9	40S ribosomal protein S7	1.03	0.045	1.15	0.009
E9AJF2	Mitochondrial processing peptide beta subunit,putative	1.71	0.002	-2.03	0.018
E9B128	Reticulon-like protein	3.28	0.014	-1.16	0.008

Supplemental Figure 9. A) All human proteins (gray circle) identified by affinity capture using 197 functionalized bead plotted Log2(fold-change) and -Log10(p-value) over control bead. Significant proteins Log2FC > 1 and p< 0.05 are shown with black circles. **B)** All human proteins (gray circle) identified by thermal profile analysis with 197 plotted z-score and -Log10(p-value). Significant proteins with a |z-score| > 1 and p< 0.05 are shown with black circles. **C)** All *Leishmania* proteins (gray circle) identified by affinity capture using 197 functionalized bead plotted Log2(fold-change) and -Log10(p-value) over control bead. Significant proteins Log2FC > 1 and p< 0.05 are shown with black circles. Proteins with a (|z-score| > 1 and p< 0.05 overlapping with significant proteins identified by affinity capture (Log2FC > 2 and p< 0.05) are shown with open circles. **D)** All *Leishmania* proteins (gray circle) identified by thermal profile analysis with 197 plotted z-score and -Log10(p-value). Significant proteins with a |z-score| > 1 and p< 0.05 are shown with black circles. Significant proteins Log2FC > 1 and p< 0.05 are shown with black circles. Proteins with a (|z-score| > 1 and p< 0.05 overlapping with significant proteins identified by affinity capture (Log2FC > 2 and p< 0.05) are shown with open circles. **E)** Values for three *Leishmania* proteins overlapping between two proteomic approaches.

bioRxiv preprint doi: https://doi.org/10.1101/2025.11.04.686469; this version posted November 4, 2025. The copyright holder for this preprint (which was not certified by peer review) is the author/funder, who has granted bioRxiv a license to display the preprint in perpetuity. It is made available under aCC-BY 4.0 International license.

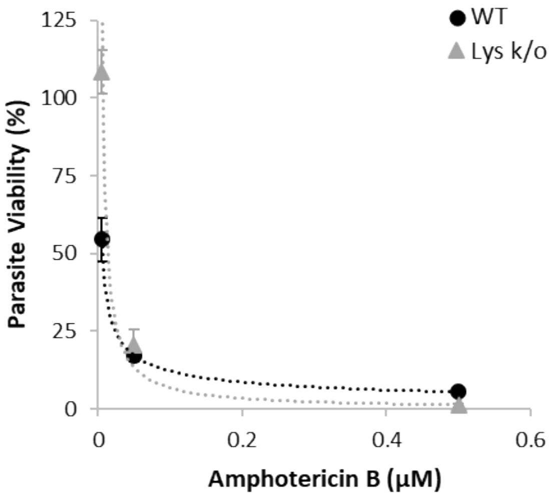

Supplemental Figure 10. Dose response of amphotericin B on intracellular *Leishmania* burden in bone marrow derived macrophages derived from wildtype C57BL/6 (WT, black circle) or lysozyme knockout mice (Lys K/O, gray triangle) as identified image-based Giemsa staining. Data is presented as mean ± standard deviation of biological triplicates.

bioRxiv preprint doi: https://doi.org/10.1101/2025.11.04.686469; this version posted November 4, 2025. The copyright holder for this preprint (which was not certified by peer review) is the author/funder, who has granted bioRxiv a license to display the preprint in perpetuity. It is made available under aCC-BY 4.0 International license.

Synthesis and Characterization of Selected Analogs:

Seven compounds were commercially available and purchased for this project. **RTI-121** (PRE-084 hydrochloride), **RTI-122** (SA4503 dihydrochloride), **RTI-123** (NE 100 hydrochloride), and **RTI-124** (BD 1047 dihydrobromide) were purchased from Tocris Bioscience, Inc. **RTI-125** (haloperidol) was acquired from Alfa Aesar. **RTI-278** ((S)-2-(3-fluorophenyl)pyrrolidine d-Tartrate) was sourced from AstaTech Inc. Lastly, **RTI-435** (notoginsenoside R1) was purchased from Sigma-Aldrich.

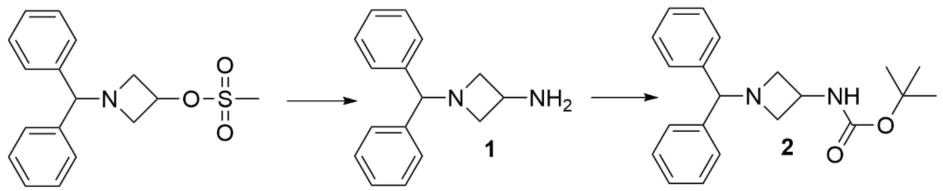

<u>RTI-11</u>: In a glass pressure reactor was combined 1-Benzhydrylazetidin-3-yl methanesulfonate (5.07 g, 15.9 mmol) and NH_4OH (19.5 mL, 288.6 mmol) in 2-propanol (30 mL). The mixture was sealed with a Teflon cap and heated to 70° C for 3 hours. The reaction mixture was quenched with saturated aqueous $NaHCO_3$ and extracted with ethyl acetate. The organic layer was washed with brine, dried (Na_2SO_4), filtered and concentrated to yield 3.76 g (100%) of **1** as an off-white gel, which required no further purification. ^{1}H-NMR ($CDCl_3$) δ 7.40-7.27 (m, 4 H), 7.24-7.15 (m, 6 H), 4.27 (s, 1 H), 3.67-3.50 (m, 3 H), 2.73-2.64 (m, 2 H).

A mixture containing **1** (3.76 g, 15.8 mmol) in tetrahydrofuran (75 mL) and 5% aqueous Na_2CO_3 (90 mL) was cooled to 0° C. A solution of di-tert-butyl decarbonate (4.48 g, 20.5 mmol) in tetrahydrofuran (15 mL) was added slowly and the reaction stirred at room temperature for 18 hours. Upon completion, the solvents were removed *in vacuo* and the residue was extracted with ethyl acetate. The organic layer was washed with brine, dried ($MgSO_4$), filtered and concentrated to yield 6.03 g of a white solid. The crude material was stirred in hexanes and the resulting solid was filtered, washed with hexanes and dried to obtain 3.91 g (73%) of **2** (**RTI-11**) as a white solid. ^{1}H-NMR ($CDCl_3$) δ 7.39 (m, 4 H), 7.29-7.15 (m, 6 H), 4.86 (br s, 1 H), 4.32-4.27 (m, 2 H), 3.52 (t, 2 H, J = 9.0 Hz), 2.87-2.79 (m, 2 H), 1.42 (s, 9 H).

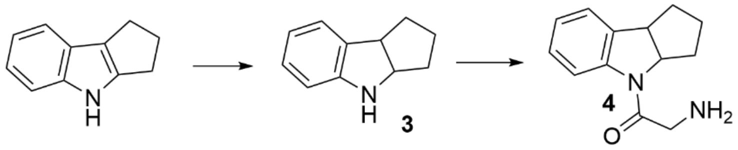

<u>RTI-320:</u> 1,2,3,4-Tetrahydrocyclopent[b]indole (4.0 g, 25.4 mmol) and 10% Palladium on Carbon (500 mg) were suspended in ethanol (50 mL) and concentrated hydrochloride acid (2.7 mL), charged with hydrogen gas and agitated on a Parr Apparatus at room temperature for 18 hours. The mixture was filtered through Celite and the pad was washed with methanol. The filtrate was concentrated, diluted with 1 N hydrochloric acid and extracted with diethyl ether. The aqueous layer was neutralized to pH of 8 with 2 N aqueous NaOH and extracted with dichloromethane. The organic layer was washed with brine, dried ($MgSO_4$), filtered and concentrated to yield 3.05 g (75%) of **3** as a brown oil, which required no further purification. ^{1}H-NMR ($CDCl_3$) δ 7.02 (d, 1 H, J = 6.0 Hz), 6.98 (d, 1 H, J = 6.0 Hz), 6.67 (dd, 1 H, J = 3.0 Hz, 6.0 Hz), 6.52 (d, 1 H, J = 9.0 Hz), 4.36 (dd, 1 H, J = 3.0 Hz, 6.0 Hz), 3.77 (t, 2 H, J = 9.0 Hz), 2.02-1.51 (m, 6 H).

A mixture containing **3** (3.05 g, 19.15 mmol), 2-chloroacetamide (3.71 g, 39.6 mmol) and diisopropylethylamine (10.3 mL, 59.1 mmol) in N, N-dimethylformamide (8 mL), in a glass pressure reactor, was sealed with a Teflon cap and heated to 100° C for 18 hours. The mixture was diluted with water and extracted with ethyl acetate. The organic layer was washed with brine, dried ($MgSO_4$), filtered and concentrated. The crude material was adsorbed onto Celite and purified over silica gel using 0-50% hexane/ethyl acetate yielding 3.74 g (86%) of **4** (**RTI-320**)

bioRxiv preprint doi: https://doi.org/10.1101/2025.11.04.686469; this version posted November 4, 2025. The copyright holder for this preprint (which was not certified by peer review) is the author/funder, who has granted bioRxiv a license to display the preprint in perpetuity. It is made available under aCC-BY 4.0 International license.

as a yellow solid. ^{1}H-NMR ($CDCl_3$) δ 7.07 (dd, 2 H, J = 6.0 Hz), 6.74 (dd, 1 H, J = 6.0 Hz, 9.0 Hz), 6.50 (br s, 1 H), 6.34 (d, 1 H, J = 9.0 Hz), 5.49 (br s, 1 H), 4.20-4.05 (m, 2 H), 3.78 (dd, 2 H, J = 9.0 Hz), 2.04-1.49 (m, 6 H). LC-MS, calculated for $C_{13}H_{16}N_2O$ $(MH)^+$ 217.3; observed 217.0. Anal. Calculated for $C_{13}H_{16}N_2O$: C, 72.19; H, 7.45; N, 12.95. Found: C, 71.93; H, 7.43; N, 12.90.

Novel Pyrazole Scaffolds (Type A: Standard Structure with N-substituted Anilines):

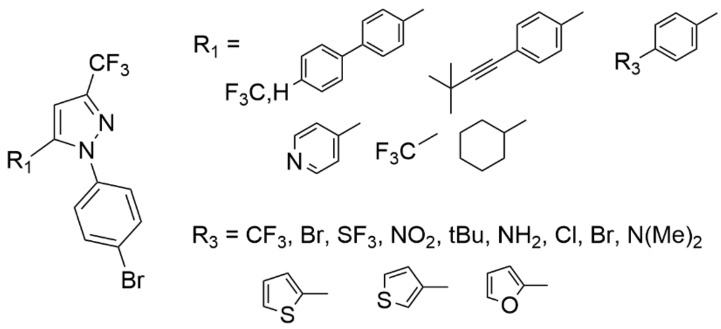

Examples:

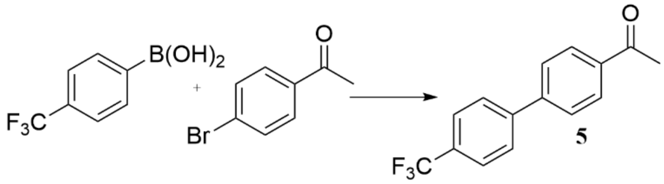

5

<u>**5**</u>: A mixture of 4-Trifluoromethylphenylboronic acid (7.84 g, 41.3 mmol), 4-Bromoacetophenone (8.25 g, 41.4 mmol), Palladium acetate (186 mg, 0.82 mmol), K_2CO_3 (17.1 g, 123.8 mmol) and Tetrabutylammonium bromide (17.3 g, 53.6 mmol) in THF (40 mL) and nitrogen gas was bubbled into the mixture for two minutes. Water (410 mL) was added and the reaction was heated to 60 C for 2 hours. Upon cooling to room temperature, the mixture was extracted with ethyl acetate. The organic material was washed with water and brine, dried ($MgSO_4$), filtered and concentrated to yield a quantitative yield (11.2 g) of a copper-colored solid (**5**), which was pure enough for further synthesis. ^{1}H-NMR ($CDCl_3$) δ 8.05 (d, 2 H, J = 6 Hz), 7.79-7.69 (m, 6 H), 2.68 (s, 3 H).

5

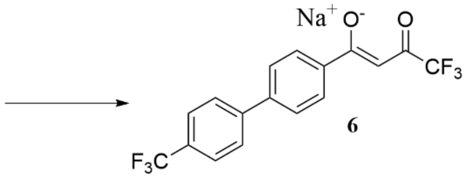

6

<u>**6**</u>: Into an oven dried flask was introduced sodium hydride (60% wt./mineral oil, 6.54 g, 272.4 mmol) and stirred in anhydrous THF (50 mL) for 5 minutes at room temperature. Ethyl trifluoroacetate (21.7 mL, 182.4 mmol) was added dropwise and this mixture stirred at room temperature for 10 minutes. A solution of **5** (24 g, 90.8 mmol) in anhydrous THF (85 mL) was added dropwise and the reaction mixture was refluxed for 3 hours. The reaction was concentrated and the residue was partitioned between ethyl acetate and water. The aqueous layer was extracted with ethyl acetate and the combined organics were washed with brine, dried (Na_2SO_4) and concentrated to yield a quantitative yield of a yellow solid (**6**), which was pure enough for the next step. ^{1}H-NMR ($CDCl_3$) δ 7.70 (d, 2 H, J = 6 Hz), 7.63 (d, 2 H, J = 9 Hz), 7.50 (d, 2 H, J = 9 Hz), 7.36 (d, 2 H, J = 9 Hz), 6.08 (s, 1 H).

6

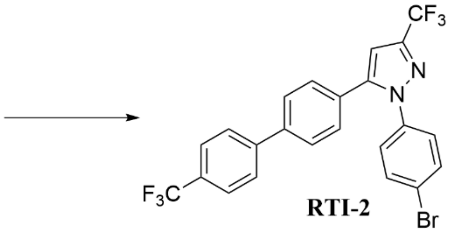

RTI-2

bioRxiv preprint doi: https://doi.org/10.1101/2025.11.04.686469; this version posted November 4, 2025. The copyright holder for this preprint (which was not certified by peer review) is the author/funder, who has granted bioRxiv a license to display the preprint in perpetuity. It is made available under aCC-BY 4.0 International license.

RTI-2: A mixture containing **6** (20 g, 52.3 mmol) and 4-bromohydrazine hydrochloride (16.4 g, 73.4 mmol) in ethanol (650 mL) was refluxed for 18 hours. The solvent was concentrated and the residue was partitioned between ethyl acetate and saturated aqueous $NaHCO_3$. The aqueous layer was extracted with ethyl acetate and the combined organics were washed with brine, dried ($MgSO_4$), filtered and concentrated. The crude material was purified over silica gel using 1-5% ethyl acetate from hexanes to yield 18.7 g of a yellow solid that contained both pyrazole isomers. The solid was crystallized from DCM/hexanes to yield 6.9 g (26%) of pure **RTI-2** as a yellow solid. ^{1}H-NMR ($CDCl_3$) δ 7.71 (dd, 4 H, J = 9 Hz), 7.60 (d, 2 H, J = 6 Hz), 7.53 (d, 2 H, J = 9 Hz), 7.31 (d, 2 H, J = 6 Hz), 7.25 (d, 2 H, J = 9 Hz), 6.81 (s, 1 H).

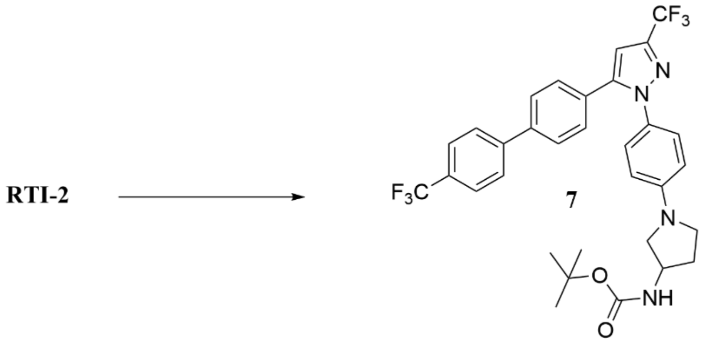

7: The following were combined in a heavy-duty glass reactor: **RTI-2** (4.65 g, 9.10 mmol), 3-N-Boc-aminopyrrolidine (2.72 g, 14.5 mmol), BINAP (1.7 g, 2.73 mmol), $Pd_2(dba)_3$ (1.08 g, 1.18 mmol) and Cs_2CO_3 (4.75 g, 14.5 mmol) in anhydrous toluene (95 mL) and nitrogen gas was bubbled into the mixture for two minutes. The reactor was then sealed with a Teflon cap and heated to 110° C for 18 hours. Upon cooling, the mixture was filtered through Celite and the filter pad was rinsed with ethyl acetate. The filtrate was washed with water and brine, dried (Na_2SO_4), filtered and concentrated. The crude material was purified over silica gel using 0-10% ethyl acetate from hexanes to yield 4.6 g (80%) of a yellow solid (**7**). ^{1}H-NMR ($CDCl_3$) δ 7.71 (dd, 4 H, J = 9 Hz), 7.56 (d, 2 H, J = 6 Hz), 7.35 (d, 2 H, J = 9 Hz), 7.18 (d, 2 H, J = 9 Hz), 6.78 (s, 1 H), 6.49 (d, 2 H, J = 9 Hz), 4.78-4.68 (m, 1 H), 4.42-4.33 (m, 1 H), 3.59 (dd, 1 H, J = 3 Hz, 6 Hz), 3.47-3.32 (m, 2 H), 3.17 (dd, 1 H, J = 3 Hz, 6 Hz), 2.33-2.26 (m, 1 H), 2.05-1.94 (m, 1 H), 1.46 (s, 9 H).

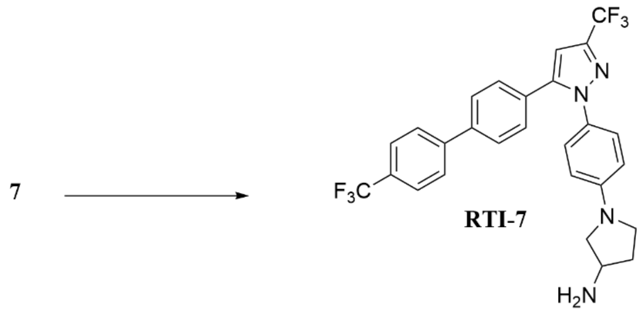

RTI-7: A solution of **7** (1.68 g, 2.72 mmol) in dichloromethane (30 mL) was cooled to 0° C and treated with trifluoroacetic acid (2.0 mL, 26.9 mmol). The reaction warmed to room temperature and stirred for 18 hours. Upon completion, the mixture was concentrated and the residue was partitioned between ethyl acetate and 2 N aqueous NaOH. The aqueous layer was extracted with ethyl acetate and the combined organic layers were washed with brine, dried (Na_2SO_4), filtered and concentrated to yield a tan solid (**RTI-7**, 1.34 g, 94%) that required no further purification. ^{1}H-NMR ($CDCl_3$) δ 7.66 (s, 4 H), 7.56 (d, 2 H, J = 9 Hz), 7.35 (d, 2 H, J = 9 Hz), 7.17 (d, 2 H, J = 9 Hz), 6.78 (s, 1 H), 6.49 (d, 2 H, J = 9 Hz), 3.75 (dd, 1 H, J = 6 Hz), 3.55-3.45 (m, 2 H), 3.35 (dd, 1 H, J = 6 Hz), 3.04 (dd, 1 H, J = 3 Hz, 6 Hz), 2.29-2.18 (m, 1 H), 1.88-1.78 (m, 1 H). ESI-MS, calculated for $C_{27}H_{22}F_6N_4$ $(MH)^+$ 517.4; observed 517.6; Anal. Calculated for $C_{27}H_{22}F_6N_4$; C, 62.79; H, 4.29; N, 10.84. Found: C, 62.72; H, 4.29; N, 10.59.

RTI-129 (5b: R-amino orientation): The product was isolated as a brown gel in 59 % yield (160 mg). ^{1}H-NMR ($CDCl_3$) δ 7.69 (s, 4 H), 7.56 (d, 2 H, J = 9 Hz), 7.35 (d, 2 H, J = 9 Hz), 7.17 (d, 2 H, J = 9 Hz), 6.78 (s, 1 H), 6.49 (d, 2 H, J = 9 Hz), 3.76 (dd, 1 H, J = 3 Hz, 6 Hz), 3.55-3.45 (m, 2 H), 3.38-3.30 (m, 1 H), 3.04 (dd, 1 H, J = 3 Hz, 6 Hz), 2.29-2.18 (m, 1 H), 1.88-1.78 (m, 1 H). ESI-MS, calculated for $C_{27}H_{22}F_6N_4$ $(MH)^+$ 517.4; observed 517.6;

bioRxiv preprint doi: https://doi.org/10.1101/2025.11.04.686469; this version posted November 4, 2025. The copyright holder for this preprint (which was not certified by peer review) is the author/funder, who has granted bioRxiv a license to display the preprint in perpetuity. It is made available under aCC-BY 4.0 International license.

Anal. Calculated for $C_{27}H_{22}F_6N_4$; C, 62.79; H, 4.29; N, 10.84. Found: C, 62.69; H, 4.37; N, 10.55; [α] = + 4.28 (c = 0.70/$CHCl_3$).

RTI-130 (5c: S-amino orientation): The product was isolated as a tan solid in 82 % yield (335 mg). ^{1}H-NMR ($CDCl_3$) δ 7.66 (s, 4 H), 7.56 (d, 2 H, J = 9 Hz), 7.35 (d, 2 H, J = 9 Hz), 7.17 (d, 2 H, J = 9 Hz), 6.78 (s, 1 H), 6.49 (d, 2 H, J = 9 Hz), 3.75 (dd, 1 H, J = 6 Hz), 3.55-3.45 (m, 2 H), 3.38-3.30 (dd, 1 H, J = 6 Hz, 9 Hz), 3.04 (dd, 1 H, J = 3 Hz, 6 Hz), 2.29-2.18 (m, 1 H), 1.88-1.78 (m, 1 H). ESI-MS, calculated for $C_{27}H_{22}F_6N_4$ $(MH)^+$ 517.4; observed 517.6; Anal. Calculated (with 0.2 mol water) for $C_{27}H_{22}F_6N_4$; C, 62.35; H, 4.34; N, 10.77. Found: C, 62.16; H, 4.37; N, 10.65; [α] = - 2.50 (c = 0.80/$CHCl_3$).

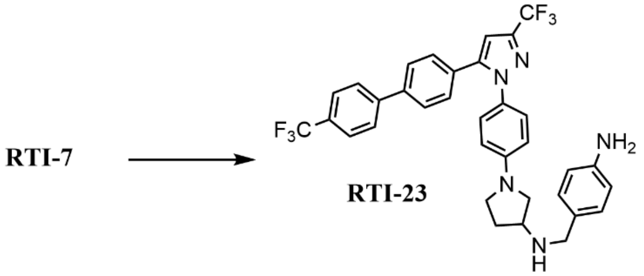

RTI-23: A solution of **RTI-7** (150 mg, 0.29 mmol), 4-nitrobenzaldehyde (44 mg, 0.29 mmol) and 4A molecular sieves (150 mg) in anhydrous methanol (3 mL) and anhydrous tetrahydrofuran (1.5 mL) was stirred at room temperature for 18 hours. The reaction was cooled to 0° C and treated with sodium borohydride (22 mg, 0.58 mmol) was added and the reaction stirred for 4 hours at room temperature. The reaction was concentrated and the residue partitioned between saturated aqueous sodium bicarbonate solution and ethyl acetate. The combined organic layers were washed with brine, dried (Na_2SO_4), filtered and concentrated to yield 203.6 mg of a brown gel. The crude material was purified over silica gel using 0-10 % methanol from dichloromethane to yield 65 mg (36%) of **RTI-23** as an off-white solid. ^{1}H-NMR ($CDCl_3$) δ 7.69 (s, 4 H), 7.56 (d, 2 H, J = 9 Hz), 7.34 (d, 2 H, J = 6 Hz), 7.14 (dd, 4 H, J = 3 Hz, 9 Hz), 6.77 (s, 1 H), 6.66 (d, 2 H, J = 6 Hz), 6.47 (d, 2 H, J = 9 Hz), 3.76 (s, 2 H), 3.63-3.41 (m, 6 H), 3.39-3.29 (m, 1 H), 3.19-3.08 (m, 1 H), 2.31-2.19 (m, 1 H), 1.99-1.88 (m, 1 H). ESI-MS, calculated for $C_{34}H_{27}F_6N_5O_2$ $(MH)^+$ 622.6; observed 622.3.

Other Selected Analogs:

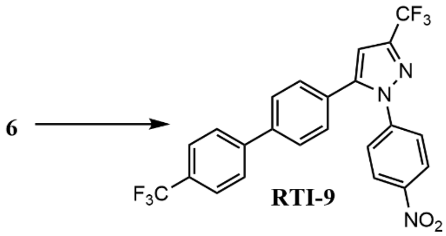

RTI-9: **A** mixture containing **6** (19.16 g, 50.1 mmol) and 4-nitrohydrazine hydrochloride (12.35 g, 65.1 mmol) in ethanol (600 mL) was refluxed for 18 hours. The solvent was concentrated and the residue was partitioned between ethyl acetate and saturated aqueous $NaHCO_3$. The aqueous layer was extracted with ethyl acetate and the combined organics were washed with brine, dried ($MgSO_4$), filtered and concentrated. The crude material was purified over silica gel using 1-5% ethyl acetate from hexanes to yield 10.58 g (44%) of pure **RTI-9** as a yellow solid. ^{1}H-NMR ($CDCl_3$) δ 8.27 (d, 2 H, J = 6 Hz), 7.75-7.69 (m, 4 H), 7.64 (d, 2 H, J = 9 Hz), 7.57 (d, 2 H, J = 6 Hz), 7.36 (d, 2 H, J = 9 Hz), 6.85 (s, 1 H). ESI-MS, calculated for $C_{23}H_{13}F_6N_3O_2$ $(MH)^+$ 477.4; observed 477.8; Anal. Calculated for $C_{23}H_{13}F_6N_3O_2$; C, 57.87; H, 2.74; N, 8.80. Found: C, 57.61; H, 2.92; N, 8.71.

bioRxiv preprint doi: https://doi.org/10.1101/2025.11.04.686469; this version posted November 4, 2025. The copyright holder for this preprint (which was not certified by peer review) is the author/funder, who has granted bioRxiv a license to display the preprint in perpetuity. It is made available under aCC-BY 4.0 International license.

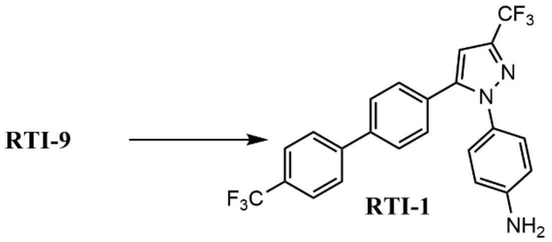

<u>RTI-1:</u> A mixture containing **RTI-9** (8.93 g, 18.7 mmol), Tin (II) chloride (12.4 g, 65.5 mmol) and concentrated hydrochloric acid (28 mL, 336 mmol) in ethanol (125 mL) was heated to 50 °C for 2.5 hours. The solvent was concentrated and the residue was partitioned between ethyl acetate and 2 N NaOH. The aqueous layer was extracted with ethyl acetate and the combined organics were washed with water and brine, dried ($MgSO_4$), filtered and concentrated to yield 8.15 g (97 %) of **RTI-1** as a tan solid, which was used without any further purification. ^{1}H-NMR ($CDCl_3$) δ 7.68 (dd, 4 H, J = 9 Hz), 7.56 (d, 2 H, J = 9 Hz), 7.35 (d, 2 H, J = 9 Hz), 7.13 (d, 2 H, J = 9 Hz), 6.78 (s, 1 H), 6.66 (d, 2 H, J = 9 Hz), 3.82 (br s, 2 H).

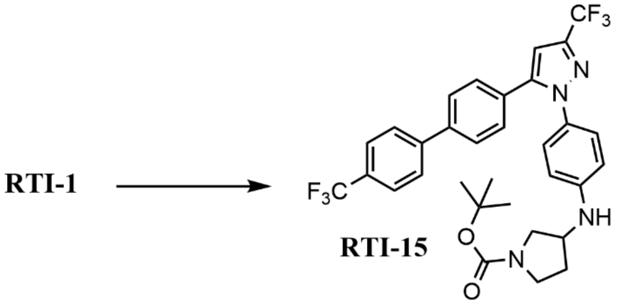

<u>RTI-15:</u> A mixture of **RTI-1** (6 g, 13.4 mmol) and N-Boc-3-pyrrolidinone (3.23 g, 17.4 mmol) in glacial acetic acid (110 mL) was treated with anhydrous sodium sulfate (11.43 g, 80.4 mmol); the mixture was then cooled to 0° C. Sodium triacetoxyborohydride (5.97 g, 28.2 mmol) was added and the reaction was stirred at room temperature for 18 hours. Upon completion, the reaction was concentrated and the residue was partitioned between ethyl acetate and saturated aqueous sodium bicarbonate. The aqueous layer was extracted with ethyl acetate. The organic layer was washed with brine, dried (Na_2SO_4), filtered and concentrated to yield 10.58 g of a black gel. The crude material was purified over silica gel using 0-20 % ethyl acetate from hexanes to yield 5.18 g (63 %) of **RTI-15** as a tan solid. ^{1}H-NMR ($CDCl_3$) δ 7.69 (s, 4 H), 7.60 (d, 2 H, J = 9 Hz), 7.36 (d, 2 H, J = 9 Hz), 7.16 (d, 2 H, J = 9 Hz), 6.78 (s, 1 H), 6.57 (d, 2 H, J = 9 Hz), 4.10-4.00 (m, 1 H), 3.98-3.90 (m, 1 H), 3.78-3.65 (m, 1 H), 3.56-3.45 (m, 2 H), 3.37-3.19 (m 1 H), 2.26-2.18 (m,1 H), 1.97-1.86 (m, 1 H), 1.46 (s, 9 H). ESI-MS, calculated for $C_{32}H_{30}F_6N_4O_2$ $(M+Na)^+$ 639.6; observed 639.5.

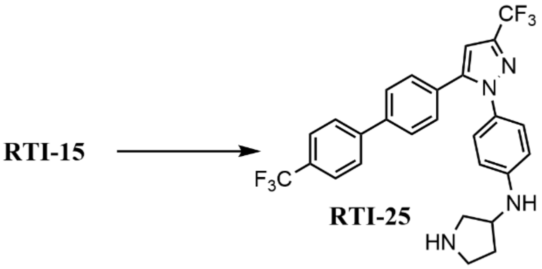

<u>RTI-25:</u> A solution of **RTI-15** (4.90 g, 7.96 mmol) in dichloromethane (55 mL) was cooled to 0° C and treated with trifluoroacetic acid (5.9 mL, 79.4 mmol). The reaction warmed to room temperature and stirred for 18 hours. Upon completion, the mixture was concentrated and the residue was partitioned between ethyl acetate and 2 N aqueous NaOH. The aqueous layer was extracted with ethyl acetate and the combined organic layers were washed with brine, dried (Na_2SO_4), filtered and concentrated to yield a brown solid (**RTI-25**, 4.0 g, 97%) that required no further purification. ^{1}H-NMR ($CDCl_3$) δ 7.68 (dd, 4 H, J = 9 Hz), 7.56 (d, 2 H, J = 9 Hz), 7.36 (d, 2 H, J = 9 Hz), 7.14 (d, 2 H, J = 9 Hz), 6.78 (s, 1 H), 6.56 (d, 2 H, J = 9 Hz), 4.05-3.91 (m, 2 H), 3.21-3.08 (m, 2 H), 3.01-2.93 (m, 1 H), 2.91-2.85 (m, 1 H), 2.27-2.15 (m, 1 H). Anal. Calculated (with 0.8 mol of water) for $C_{27}H_{22}F_6N_4$; C, 61.08; H, 4.48; N, 10.55. Found: C, 61.23; H, 4.30; N, 10.43.

bioRxiv preprint doi: https://doi.org/10.1101/2025.11.04.686469; this version posted November 4, 2025. The copyright holder for this preprint (which was not certified by peer review) is the author/funder, who has granted bioRxiv a license to display the preprint in perpetuity. It is made available under aCC-BY 4.0 International license.

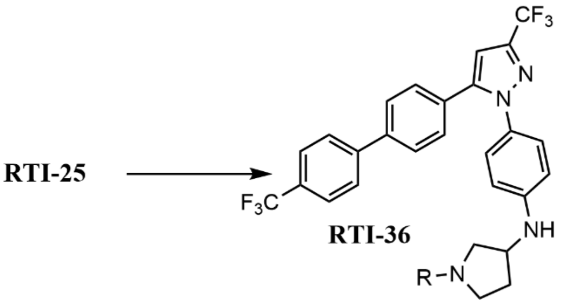

RTI-36 [R = CH_2-(4-biphenyl)]: A solution of **RTI-25** (150 mg, 0.29 mmol), biphenyl 4-carboxaldehyde (58 mg, 0.32 mmol) and 4A molecular sieves (150 mg) in anhydrous 1,2-dichloroethane (4 mL) was stirred at room temperature for 5 hours. Sodium triacetoxyborohydride (136 mg, 0.64 mmol) was added and the reaction stirred for 18 hours at room temperature. The reaction was quenched with saturated aqueous sodium bicarbonate solution and extracted with ethyl acetate and the combined organic layers were washed with brine, dried (Na_2SO_4), filtered and concentrated to yield 176 mg of a brown solid. The crude material was purified over silica gel using 0-40 % ethyl acetate from hexanes to yield 85.7 mg (43%) of **RTI-36** as a white solid. ^{1}H-NMR ($CDCl_3$) δ 7.68 (dd, 4 H, J = 9 Hz), 7.60-7.53 (m, 6 H), 7.46-7.33 (m, 7 H), 7.12 (d, 2 H, J = 6 Hz), 6.77 (s, 1 H), 6.54 (d, 2 H, J = 9 Hz), 4.11 (dd, 1 H, J = 6 Hz), 4.01 (br s, 1 H), 3.68 (s, 2 H), 2.86-2.75 (m, 2 H), 2.62 (d, 1 H, J = 3 Hz, 6 Hz), 2.48 (dd, 1 H, J = 6 Hz, 9 Hz), 2.34 (dd, 1 H, J = 6 Hz, 9 Hz), 1.77-1.65 (m, 1 H). ESI-MS, calculated for $C_{40}H_{32}F_6N_4$ $(MH)^+$ 683.7; observed 683.7.

RTI-53 [R = CH_2-(3-cyanophenyl)]: Using 3-cyanobenzaldehyde, the product was isolated as a white solid in 58% yield (142 mg). ^{1}H-NMR ($CDCl_3$) δ 7.65 (dd, 5 H, J = 12 Hz), 7.55 (d, 2 H, J = 9 Hz), 7.43 (m, d, 1 H, J = 6 Hz), 7.35 (dd, 2 H, J = 9 Hz), 7.14 (dd, 2 H, J = 9 Hz), 6.77 (s, 1 H), 6.55 (d, 2 H, J = 9 Hz), 4.16-4.03 (m, 2 H), 3.65 (s, 2 H), 2.84-2.71 (m, 2 H), 2.59 (dd, 1 H, J = 9 Hz), 2.46-2.28 (m, 2 H), 1.76-1.65 (m, 1 H). ESI-MS, calculated for $C_{35}H_{27}F_6N_5$ $(MH)^+$ 632.6; observed 632.6.

RTI-91 [R = CH_2-(2-hydroxyphenyl)]: Using 2-hydroxybenzaldehyde, the product was isolated as an off-white solid in 48% yield (138.6 mg). ^{1}H-NMR ($CDCl_3$) δ 7.68 (dd, 4 H, J = 9 Hz), 7.56 (d, 2 H, J = 9 Hz), 7.35 (dd, 2 H, J = 9 Hz), 7.16 (dd, 3 H, J = 6 Hz, 9 Hz), 6.99 (d, 1 H, J = 9 Hz), 6.84-6.76 (m, 3 H), 6.53 (d, 2 H, J = 9 Hz), 4.06-3.97 (m, 2 H), 3.84 (s, 2 H), 2.97-2.85 (m, 2 H), 2.70 (dd, 1 H, J = 3 Hz, 6 Hz), 2.56 (dd, 1 H, J = 9 Hz), 2.42 (dd, 1 H, J = 6 Hz, 9 Hz), 1.78-1.72 (m, 1 H). ESI-MS, calculated for $C_{34}H_{28}F_6N_4O$ $(MH)^+$ 623.6; observed 623.8.

Preparation of Novel Pyrazole Scaffolds (Type B: Left-Ring Substitution with N-substituted Anilines):

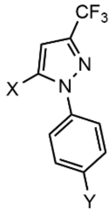

Following procedures described above, a few examples include:

RTI-247 (X = Cyclohexyl, Y = 3-Aminopyrrolidinyl): Using 1-Cyclohexylethanone in the procedure for **RTI-7**, **RTI-247** was isolated as a tan oil in 65% yield (125 mg). ^{1}H-NMR ($CDCl_3$) δ 7.26 (d, 2 H, J = 9 Hz), 6.54 (d, 2 H, J = 9 Hz), 6.53 (s, 1 H), 3.81-3.71 (m, 1 H), 3.61-3.49 (m, 2 H), 3.05 (dd, 1 H, J = 3 Hz, 6 Hz), 2.79-2.67 (m, 1 H), 2.32-2.21 (m, 1 H), 2.08-2.00 (m, 2 H), 1.91-1.71 (m, 4 H), 1.60-1.22 (m, 5 H). ESI-MS, calculated for $C_{20}H_{25}F_3N_4$ $(MH)^+$ 379.4; observed 379.4; Anal. Calculated for $C_{20}H_{25}F_3N_4$; C, 63.47; H, 6.65; N, 14.80. Found: C, 63.31; H, 6.50; N, 14.57.

bioRxiv preprint doi: https://doi.org/10.1101/2025.11.04.686469; this version posted November 4, 2025. The copyright holder for this preprint (which was not certified by peer review) is the author/funder, who has granted bioRxiv a license to display the preprint in perpetuity. It is made available under aCC-BY 4.0 International license.

<u>**RTI-272 (X = Cyclopropyl, Y = 3-Aminopyrrolidinyl)**</u>: **RTI-272** was isolated as a tan solid (139.6 mg, 24%, over 2 steps). ^{1}H-NMR ($CDCl_3$) δ 7.34 (d, 2 H, J = 6 Hz), 6.64 (d, 2 H, J = 6 Hz), 6.14 (s, 1 H), 4.07-3.94 (m, 2 H), 3.19-3.07 (m, 2 H), 3.00-2.85 (m, 2 H), 2.28-2.14 (m, 1 H), 1.79-1.68 (m, 3 H), 1.02-0.95 (m, 2 H), 0.78-0.73 (m, 2 H). NMR ($CDCl_3$, 75 MHz) δ 147.6, 129.2, 126.6, 112.9, 99.2, 54.0, 53.9, 45.8, 33.7, 8.8, 7.4. ESI-MS, calculated for $C_{17}H_{19}F_3N_4$ $(MH)^+$ 337.4; observed 337.4. Anal. Calculated for $C_{17}H_{19}F_3N_4$; C, 60.70; H, 5.69; N, 16.65. Found: C, 60.49; H, 5.80; N, 16.50.

<u>**RTI-273 (X = 2-Furanyl, Y = 3-Aminopyrrolidinyl)**</u>: **RTI-273** was isolated as a tan solid (382 mg, 37%, over 2 steps). ^{1}H-NMR ($CDCl_3$) δ 7.42 (d, 2 H, J = 3 Hz), 7.20 (d, 2 H, J = 6 Hz), 6.87 (s, 1 H), 6.63 (d, 2 H, J = 6 Hz), 6.33 (d, 1 H, J = 3 Hz), 5.92 (d, 1 H, J = 3 Hz), 4.14-3.95 (m, 2 H), 3.22-3.08 (m, 2 H), 3.01-2.86 (m, 2 H), 2.29-2.19 (m, 1 H), 1.76-1.67 (m, 1 H). NMR ($CDCl_3$, 75 MHz) δ 148.3, 143.6, 142.8, 136.4, 129.1, 127.4, 119.4, 112.9, 111.3, 109.3, 102.8, 53.9, 53.8, 45.8, 33.6. ESI-MS, calculated for $C_{18}H_{17}F_3N_4O$ $(MH)^+$ 363.3; observed 363.4. Anal. Calculated for $C_{18}H_{17}F_3N_4O$; C, 59.75; H, 4.73; N, 15.48. Found: C, 59.36; H, 4.84; N, 15.52.

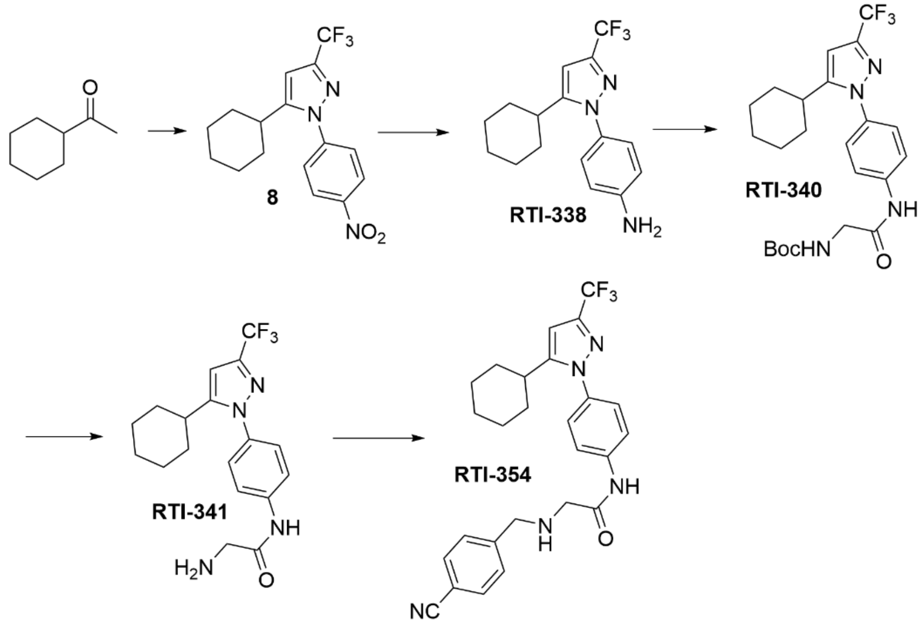

<u>**8:**</u> Into an oven dried flask was introduced sodium hydride (60% wt./mineral oil, 9.52 g, 237.6 mmol) and stirred in anhydrous THF (140 mL) for 5 minutes at room temperature. Ethyl trifluoroacetate (18.9 mL, 158.8 mmol) was added dropwise and this mixture stirred at room temperature for 10 minutes. A solution of cyclohexyl methyl ketone (10 g, 79.2 mmol) in anhydrous THF (40 mL) was added dropwise and the reaction mixture was refluxed for 3 hours. The reaction was concentrated and the residue was partitioned between ethyl acetate and water. The aqueous layer was extracted with ethyl acetate and the combined organics were washed with brine, dried (Na_2SO_4) and concentrated to yield a quantitative yield of a yellow oil, which was used without further purification. ^{1}H-NMR (CD_3OD) δ 5.24 (s, 1 H), 2.94-2.84 (m, 1 H), 1.82-1.66 (m, 4 H), 1.40-1.19 (m, 6 H). Using this material, the procedure for **RTI-2** was followed, substituting 4-nitrohydrazine hydrochloride. During pyrazole formation, two different pyrazoles may form. The product (**8**, the less polar isomer, with approximate R_f = 0.7) was isolated as a yellow oil in 59% yield (15.9 g). ^{1}H-NMR ($CDCl_3$) δ 8.36 (d, 2 H, J = 9 Hz), 7.73 (d, 2 H, J = 9 Hz), 6.72 (s, 1 H), 2.77-2.69 (m, 1 H), 1.86-1.73 (m, 4 H), 1.59-1.22 (m, 6 H).

<u>**RTI-338:**</u> **A** mixture containing **8** (21.8 mmol), stannous chloride (14.5 g, 76.3 mmol) and concentrated HCl (33 mL, 396 mmol) in ethanol (145 mL) was heated to 50° C for 2.5 hours. Upon cooling to room temperature, the solvent was concentrated and the residue was diluted with ethyl acetate and 2 N NaOH (300 mL) was stirred in the mixture stirred at room temperature for 1h. The aqueous layer was extracted with ethyl acetate and the combined organics were washed with water and brine, dried ($MgSO_4$), filtered and concentrated. The crude material was adsorbed onto silica gel and purified via ISCO using 1-20% ethyl acetate from hexanes to yield 3.54 g (52%) of a white solid (**RTI-338**). ^{1}H-NMR ($CDCl_3$) δ 7.22 (d, 2 H, J = 9 Hz), 6.70 (d, 2 H, J = 9 Hz), 6.54 (s, 1 H), 3.83 (br s, 2 H), 2.74-2.65 (m, 1 H), 2.05-1.96 (m, 2 H), 1.84-1.71 (m, 2 H), 1.51-1.31 (m, 6 H). ^{13}C NMR

bioRxiv preprint doi: https://doi.org/10.1101/2025.11.04.686469; this version posted November 4, 2025. The copyright holder for this preprint (which was not certified by peer review) is the author/funder, who has granted bioRxiv a license to display the preprint in perpetuity. It is made available under aCC-BY 4.0 International license.

($CDCl_3$, 75 MHz) δ 147.1, 127.0, 114.7, 105.2, 60.3, 37.3, 33.0, 26.2, 26.0, 14.1; ESI-MS, calculated for $C_{16}H_{18}F_3N_3$ $(MH)^+$ 310.3; observed 310.0. Anal. Calculated for $C_{16}H_{18}F_3N_3$; C, 62.12; H, 5.86; N, 13.58. Found: C, 62.06; H, 5.98; N, 13.44.

RTI-340: A mixture containing **RTI-338** (3.03 g, 9.79 mmol), Boc-glycine (3.77 g, 21.5 mmol), diisopropylethylamine (6.0 mL, 34.4 mmol), 4-N,N-dimethylaminopyridine (180 mg, 1.47 mmol) and N-(3-dimethylaminopropyl)-n-ethylcarbodiimide hydrochloride (4.13 g, 21.5 mmol) in tetrahydrofuran (90 mL) was stirred at room temperature for 18 hours. The solvent was concentrated and the residue was partitioned between ethyl acetate and 1 N HCl. The aqueous layer was extracted with ethyl acetate and the combined organics were washed with saturated aqueous $NaHCO_3$, water and brine, dried (Na_2SO_4), filtered and concentrated. The crude material was adsorbed onto silica gel and purified via ISCO using 3-20% ethyl acetate from hexanes to yield 3.60 g (72%) of a white solid (**RTI-340**). ^{1}H-NMR ($CDCl_3$) δ 8.35 (br s, 1 H), 7.64 (d, 2 H, J = 9 Hz), 7.43 (d, 2 H, J = 9 Hz), 6.59 (s, 1 H), 5.22 (br s, 1 H), 3.94 (d, 2 H, J = 6 Hz), 2.71 (dd, 1 H, J = 3 Hz, 9 Hz), 2.07-2.00 (m, 2 H), 1.84-1.62 (m, 3 H), 1.49 (s, 9 H), 1.44-1.24 (m, 5 H). ^{13}C NMR ($CDCl_3$, 75 MHz) δ 126.3, 119.8, 37.3, 33.0, 28.2, 26.2, 26.0; ESI-MS, calculated for $C_{23}H_{29}F_3N_4O_3$ $(MH)^+$ 467.5; observed 467.0. Anal. Calculated for $C_{23}H_{29}F_3N_4O_3$; C, 59.21; H, 6.26; N, 12.01. Found: C, 59.12; H, 6.26; N, 12.16.

RTI-341: A solution of **RTI-340** (3.36 g, 7.84 mmol) in dichloromethane (80 mL) was cooled to 0° C and treated with trifluoroacetic acid (5.9 mL, 79.4 mmol). The reaction warmed to room temperature and stirred for 18 hours. Upon completion, the mixture was concentrated and the residue was partitioned between ethyl acetate and 2 N aqueous NaOH. The aqueous layer was extracted with ethyl acetate and the combined organic layers were washed with brine, dried (Na_2SO_4), filtered and concentrated to yield a tan solid. The crude material was purified over silica gel using 0-5% methanol from dichloromethane to yield 2.60 g (90%) of a white solid. Some of the crude material (400 mg) was adsorbed onto silica gel and purified via ISCO using 0-20% methanol from dichloromethane to yield 372 mg (93%) of a white solid (**RTI-341**). ^{1}H-NMR ($CDCl_3$) δ 9.59 (br s, 1 H), 7.73 (d, 2 H, J = 9 Hz), 7.44 (d, 2 H, J = 9 Hz), 6.59 (s, 1 H), 3.50 (s, 2 H), 2.72 (dd, 1 H, J = 3 Hz, 9 Hz), 2.08-1.96 (m, 2 H), 1.88-1.71 (m, 3 H), 1.52-1.33 (m, 5 H). ^{13}C NMR ($CDCl_3$, 75 MHz) δ 170.7, 158.6, 138.2, 136.5, 126.3, 119.3, 105.9, 45.1, 37.3, 33.0, 28.2, 26.2, 26.0; ESI-MS, calculated for $C_{18}H_{21}F_3N_4O$ $(MH)^+$ 367.3; observed 367.0. Anal. Calculated for $C_{18}H_{21}F_3N_4O$; C, 59.00; H, 5.77; N, 15.29. Found: C, 58.97; H, 5.81; N, 15.17.

RTI-354: A mixture containing **RTI-341** (300 mg, 0.82 mmol), 4-cyanobenzyl bromide (164 mg, 0.835 mmol) and triethylamine (0.29 mL, 2.08 mmol) in dimethylformamide (7 mL) was stirred for 18 hours at room temperature. The reaction was poured into a saturated aqueous LiCl solution and extracted with ethyl ether. The organic layer was washed with brine, dried (Na_2SO_4), filtered and concentrated. The crude material was purified over silica gel using 1-5% methanol from dichloromethane to yield 212 mg (54%) of a white solid (**RTI-354**). ^{1}H-NMR ($CDCl_3$) δ 9.19 (s, 1 H), 7.68 (d, 2 H, J = 9 Hz), 7.45 (dd, 2 H, J = 6 Hz, 9 Hz), 6.60 (s, 1 H), 3.95 (s, 2 H), 3.46 (s, 2 H), 2.72 (dd, 1 H, J 3 Hz, 6 Hz), 2.07-1.99 (m, 2 H), 1.85-1.72 (m, 5 H), 1.52-1.25 (m, 6 H). ^{13}C NMR ($CDCl_3$, 75 MHz) δ 169.0, 158.4, 144.2, 137.9, 135.2, 132.6, 128.6, 126.4, 119.4, 111.6, 106.0, 53.5, 52.4, 37.3, 33.0, 26.2, 25.9; ESI-MS, calculated for $C_{26}H_{26}F_3N_5O$ $(MH)^+$ 482.5; observed 482.0. Anal. Calculated for $C_{26}H_{26}F_3N_5O$; C, 64.85; H, 5.44; N, 14.54. Found: C, 64.56; H, 5.51; N, 14.48.

Examples of Imidazole Structure Synthesis:

bioRxiv preprint doi: https://doi.org/10.1101/2025.11.04.686469; this version posted November 4, 2025. The copyright holder for this preprint (which was not certified by peer review) is the author/funder, who has granted bioRxiv a license to display the preprint in perpetuity. It is made available under aCC-BY 4.0 International license.

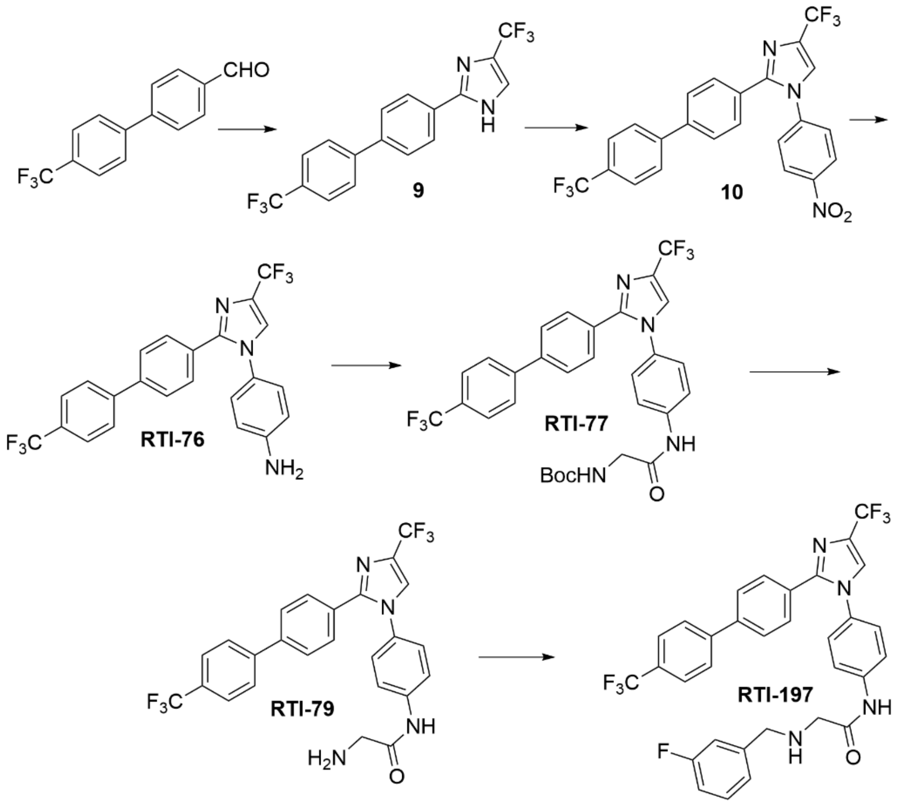

9: A mixture containing 1,1-Dibromo-3,3,3-trifluoroacetone (3.3 g, 12.0 mmol) and sodium acetate (1.32 g, 16.0 mmol) in water (42 mL) was heated to 95° C for 1 hour, then cooled to room temperature. 4'-Trifluoromethylbiphenyl-4-carboxaldehyde (2.0 g, 8.0 mmol) in methanol (102 mL) was cooled to 0° C and treated with dropwise addition of ammonium hydroxide (49 mL, 725.2 mmol). This mixture stirred for 10 minutes at 0° C and then the first mixture was added dropwise; the combined reaction slowly warmed to room temperature and stirred for 18 hours. The resulting solids were filtered, washed with water and dried and the filtrate was concentrated. The dried solids and filtrate residue were partitioned between dichloromethane and water. The organic layer was washed with brine, dried (Na_2SO_4) and concentrated. The crude material was adsorbed onto silica gel and purified via ISCO using 0-30% ethyl acetate from hexanes to yield 727 mg (25%) of a yellow solid (**9**). ^{1}H-NMR ($CDCl_3$) δ 8.00-7.95 (m, 1 H), 7.75-7.68 (m, 7 H), 7.64-7.57 (m, 2 H); LC-MS, calculated for $C_{17}H_{10}F_6N_2$ $(MH)^+$ 357.2; observed 357.0.

10: A mixture containing **9** (727 mg, 2.04 mmol), 4-fluoronitrobenzene (864 mg, 6.12 mmol) and K_2CO_3 (1.0 g, 7.23 mmol) in N, N-dimethylformamide (21 mL) was heated to 90° C for 21 hours. Upon cooling to room temperature, the reaction was poured into saturated aqueous LiCl solution and extracted with ethyl acetate. The combined organics were washed with water (5 x) and brine, dried ($MgSO_4$), filtered and concentrated. The crude material was adsorbed onto silica gel and purified via ISCO using 1-20% ethyl acetate from hexanes to yield 465 mg (48%) of an off-white solid (**10**). ^{1}H-NMR ($CDCl_3$) δ 8.36 (d, 2 H, J = 9 Hz), 7.70 (dd, 4 H, J = 6 Hz, 9 Hz), 7.57 (d, 3 H, J = 6 Hz), 7.49 (d, 4 H, J = 9 Hz); ESI-MS, calculated for $C_{23}H_{13}F_6N_3O_2$ $(MH)^+$ 478.3; observed 478.0.

RTI-76: A mixture containing **10** (465 mg, 0.974 mmol), stannous chloride dihydrate (770 mg, 3.41 mmol) and concentrated HCl (1.5 mL, 18.0 mmol) in ethanol (10 mL) was heated to 50° C for 3.5 hours. Upon cooling to room temperature, the solvent was concentrated and the residue was diluted with ethyl acetate and 2 N NaOH. The aqueous layer was extracted with ethyl acetate and the combined organics were washed with water and brine, dried (Na_2SO_4), filtered and concentrated to yield 537 mg (> 100%) of a yellow gel (**RTI-76**), which required no further purification. ^{1}H-NMR ($CDCl_3$) δ 7.69 (dd, 4 H, J = 6 Hz, 9 Hz), 7.52 (dd, 4 H, J = 6 Hz, 9 Hz), 7.44 (s, 1 H), 7.07 (dd, 2 H, J = 9 Hz), 6.71 (d, 2 H, J = 9 Hz), 3.91 (br s, 2 H).

bioRxiv preprint doi: https://doi.org/10.1101/2025.11.04.686469; this version posted November 4, 2025. The copyright holder for this preprint (which was not certified by peer review) is the author/funder, who has granted bioRxiv a license to display the preprint in perpetuity. It is made available under aCC-BY 4.0 International license.

RTI-77: A mixture containing **RTI-76** (0.974 mmol), Boc-glycine (260 mg, 1.46 mmol), diisopropylethylamine (0.6 mL, 3.44 mmol) and Propylphosphonic anhydride solution, 50 wt. % in ethyl acetate (1.8 mL, 3.02 mmol) in anhydrous tetrahydrofuran (35 mL) was sealed tightly and stirred at room temperature for 68 hours. The solvent was concentrated to 20% volume and the residue was partitioned between ethyl acetate and saturated aqueous $NaHCO_3$. The organic layer was washed with brine, dried (Na_2SO_4), filtered and concentrated. The crude material was adsorbed onto silica gel and purified via ISCO using 1-50% ethyl acetate from hexanes to yield 542 mg (92%) of an off-white solid (**RTI-77**). ^{1}H-NMR ($CDCl_3$) δ 8.52 (br s, 1 H), 7.71-7.63 (m, 7 H), 7.53-7.47 (m, 5 H), 7.28-7.22 (m, 1 H), 5.24 (br s, 1 H), 3.94 (d, 2 H, J = 6 Hz), 1.49 (s, 9 H).

RTI-79: A solution of **RTI-77** (540 mg, 0.893 mmol) in dichloromethane (26 mL) was cooled to 0° C and treated with trifluoroacetic acid (1.4 mL, 18.8 mmol). The reaction warmed to room temperature and stirred for 18 hours. Upon completion, the mixture was concentrated and the residue was partitioned between ethyl acetate and 2 N aqueous NaOH. The aqueous layer was extracted with ethyl acetate and the combined organic layers were washed with brine, dried (Na_2SO_4), filtered and concentrated to yield 405 mg (90%) of a yellow solid (**RTI-79**), which required no further purification. ^{1}H-NMR ($CDCl_3$) δ 9.68 (br s, 1 H), 7.72 (d, 2 H, J = 9 Hz), 7.64 (dd, 4 H, J = 9 Hz), 7.55-7.49 (m, 5 H), 7.25 (d, 2 H, J = 6 Hz), 3.52 (s, 2 H).

RTI-197: A mixture containing **RTI-79** (405 mg, 0.805 mmol), 3-fluorobenzyl bromide (0.11 mL, 0.897 mmol) and triethylamine (0.28 mL, 2.01 mmol) in dimethylformamide (7 mL) was stirred for 25 hours at room temperature. The reaction was poured into a saturated aqueous LiCl solution and extracted with ethyl ether. The organic layer was washed with brine, dried (Na_2SO_4), filtered and concentrated to obtain 523 mg of a yellow gel. The crude material was purified over silica gel using 0-5% methanol from dichloromethane to yield 212 mg (43%) of an off-white solid (**RTI-197**). ^{1}H-NMR ($CDCl_3$) δ 9.39 (s, 1 H), 7.71-7.64 (m, 6 H), 7.54-7.47 (m, 6 H), 7.37-7.23 (m, 3 H), 7.11-6.96 (m, 3 H), 3.88 (s, 2 H), 3.47 (s, 2 H). LC-MS, calculated for $C_{32}H_{23}F_7N_4O$ $(MH)^+$ 613.5; observed 613.2. Anal. Calculated for $C_{32}H_{23}F_7N_4O$; C, 62.74; H, 3.78; N, 9.14. Found: C, 62.46; H, 3.86; N, 9.09.

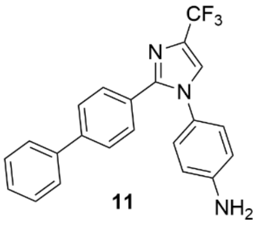

11

11: Using (1,1'-biphenyl)-4-carboxaldehyde as the starting material through the procedures to prepare **RTI-76**, **11** was isolated as a yellow solid (3.81 g). ^{1}H-NMR ($CDCl_3$) δ 7.61 (dd, 2 H, J = 6 Hz), 7.50 (dd, 4 H, J = 3 Hz, 6 Hz), 7.45-7.32 (m, 4 H), 7.05 (d, 2 H, J = 6 Hz), 6.71 (d, 2 H, J = 6 Hz), 3.89 (br s, 2 H).

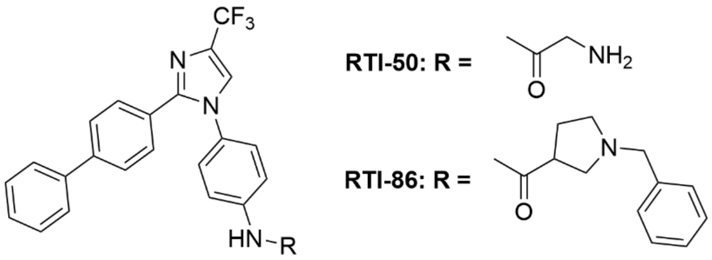

RTI-50: Following the procedures for preparing **RTI-77** and **RTI-79**, **RTI-50** was isolated as an off-white solid (264 mg, 79% for the final step). ^{1}H-NMR ($CDCl_3$) δ 9.66 (br s, 1 H), 7.74 (d, 2 H, J = 9 Hz), 7.57 (d, 2 H, J = 9 Hz), 7.50 (dd, 4 H, J = 9 Hz), 7.43 (dd, 2 H, J = 6 Hz, 9 Hz), 7.36 (d, 1 H, J = 9 Hz), 7.32-7.24 (m, 3 H), 3.52 (s, 2 H). ESI-MS, calculated for $C_{24}H_{19}F_3N_4O$ $(MH)^+$ 437.4; observed 437.0. Anal. Calculated (with 0.2 mol water) for $C_{24}H_{19}F_3N_4O$; C, 65.50; H, 4.44; N, 12.73. Found: C, 66.04; H, 4.38; N, 12.83.

bioRxiv preprint doi: https://doi.org/10.1101/2025.11.04.686469; this version posted November 4, 2025. The copyright holder for this preprint (which was not certified by peer review) is the author/funder, who has granted bioRxiv a license to display the preprint in perpetuity. It is made available under aCC-BY 4.0 International license.

RTI-86: A mixture containing **11** (400 mg, 1.05 mmol), 1-(Phenylmethyl)-3-pyrrolidinecarboxylic acid (325 mg, 1.58 mmol), diisopropylethylamine (0.65 mL, 3.73 mmol) and Propylphosphonic anhydride solution, 50 wt. % in ethyl acetate (1.9 mL, 3.19 mmol) in anhydrous tetrahydrofuran (38 mL) was sealed tightly and stirred at room temperature for 18 hours. The solvent was concentrated to 20% volume and the residue was partitioned between ethyl acetate and saturated aqueous $NaHCO_3$. The organic layer was washed with brine, dried (Na_2SO_4), filtered and concentrated. The crude material was adsorbed onto silica gel and purified via ISCO using 0-10% methanol from dichloromethane to yield 513 mg (86%) of an off-white solid (**RTI-86**). ^{1}H-NMR ($CDCl_3$) δ 9.77 (s, 1 H), 7.57 (dd, 4 H, J = 6 Hz, 9 Hz), 7.48 (dd, 6 H, J = 6 Hz), 7.42 (d, 1 H, J = 6 Hz), 7.37-7.32 (m, 5 H), 7.29-7.20 (m, 4 H), 3.73 (dd, 2 H, J = 6 Hz, 12 Hz), 3.16 (dd, 2 H, J = 6 Hz, 9 Hz), 2.95 (dd, 1 H, J = 6 Hz, 9 Hz), 2.42-2.33 (m, 3 H), 2.12-2.05 (m, 1 H). LC-MS, calculated for $C_{34}H_{29}F_3N_4O$ $(MH)^+$ 567.6; observed 567.2. Anal. Calculated for $C_{34}H_{29}F_3N_4O$; C, 72.07; H, 5.15; N, 9.88. Found: C, 71.97; H, 5.25; N, 9.82.

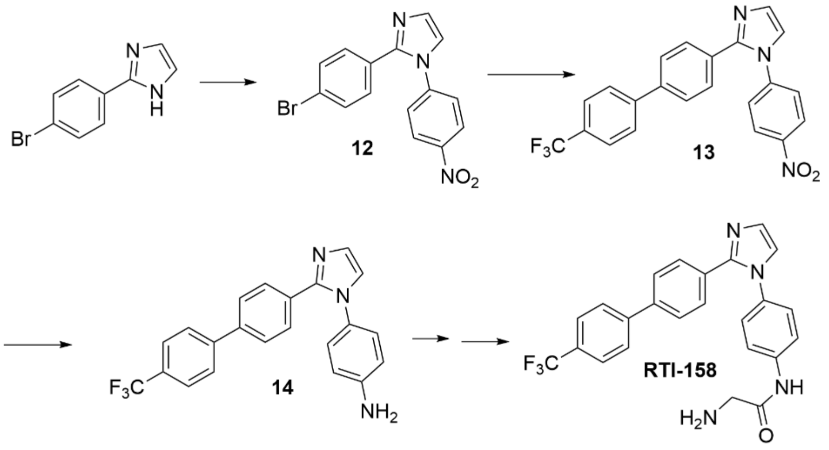

12: A mixture containing 2-(4-Bromophenyl)-1H-imidazole (500 mg, 2.25 mmol), 4-fluoronitrobenzene (950 mg, 6.74 mmol) and K_2CO_3 (930 mg, 6.74 mmol) in N, N-dimethylformamide (21 mL) was heated to 90° C for 21 hours. Upon cooling to room temperature, the poured into saturated aqueous LiCl solution and extracted with ethyl acetate. The combined organics were washed with water (5 x) and brine, dried ($MgSO_4$), filtered and concentrated. The crude material was adsorbed onto silica gel and purified via ISCO using 0-5% methanol from dichloromethane to yield 700 mg (90%) of an off-white solid (**12**).

13: A mixture containing **12** (500 mg, 1.46 mmol), 4-trifluoromethylphenylboronic acid (330 mg, 1.75 mmol), palladium tetrakis(triphenylphosphine) (170 mg, 0.146 mmol) and 1.0 M aqueous Na_2CO_3 (4.37 mL, 4.37 mmol) in ethylene glycol dimethyl ether (15 mL) was heated to 100° C for 21 hours. Upon cooling to room temperature, the reaction was diluted with water and poured extracted with ethyl acetate. The combined organics were washed with brine, dried ($MgSO_4$), filtered and concentrated. The crude material was adsorbed onto silica gel and purified via ISCO using 10-75% ethyl acetate from hexanes to yield 500 mg (84%) of an off-white solid (**13**).

14: A mixture containing **13** (500 mg, 1.22 mmol), stannous chloride dihydrate (1.85 g, 9.78 mmol) and 1.0 M HCl (24 mL, 24 mmol) in ethanol (40 mL) was heated to 50° C for 3.5 hours. Upon cooling to room temperature, the solvent was concentrated and the residue was diluted with ethyl acetate and 2 N NaOH. The aqueous layer was extracted with ethyl acetate and the combined organics were washed with water and brine, dried (Na_2SO_4), filtered and concentrated. The crude material was adsorbed onto silica gel and purified via ISCO using 0-5% methanol from dichloromethane to yield 470 mg (>100%) of an off-white solid (**14**).

RTI-158: A mixture containing **14** (50 mg, 0.132 mmol), Boc-glycine (69 mg, 0.394 mmol), diisopropylethylamine (0.15 mL, 0.792 mmol) and Propylphosphonic anhydride solution, 50 wt. % in ethyl acetate (0.25 mL, 0.394 mmol) in anhydrous tetrahydrofuran (20 mL) was sealed tightly and stirred at room temperature for 18 hours. The solvent was diluted with ethyl acetate (20 mL) and saturated aqueous $NaHCO_3$

bioRxiv preprint doi: https://doi.org/10.1101/2025.11.04.686469; this version posted November 4, 2025. The copyright holder for this preprint (which was not certified by peer review) is the author/funder, who has granted bioRxiv a license to display the preprint in perpetuity. It is made available under aCC-BY 4.0 International license.

(25 mL). The organic layer was washed with brine, dried (Na_2SO_4), filtered and concentrated. The crude material was adsorbed onto silica gel and purified via ISCO using 10-50% ethyl acetate from hexanes to yield 50 mg (0.0933 mmol, 71%) of an off-white solid, which was dissolved in methanol (5 mL), cooled to 0° C and treated with 4.0 N hydrochloric acid in dioxane (1.2 mL, 4.8 mmol). The reaction warmed to room temperature and stirred for 18 hours. Upon completion, the mixture was concentrated and the residue was partitioned between dichloromethane and saturated aqueous $NaHCO_3$. The aqueous layer was extracted with dichloromethane and the combined organic layers were washed with brine, dried (Na_2SO_4), filtered and concentrated. The crude material was adsorbed onto silica gel and purified via ISCO using 0-10% methanol from dichloromethane to yield 25 mg (63%) of an off-white solid (**RTI-158**). ^{1}H-NMR ($CDCl_3$) δ 9.60 (br s, 1 H), 7.68 (dd, 4 H, J = 8.4 Hz, 9.6 Hz), 7.49 (dd, 4 H, J = 2.0 Hz), 7.29-7.21 (m, 6 H), 3.50 (s, 2 H).

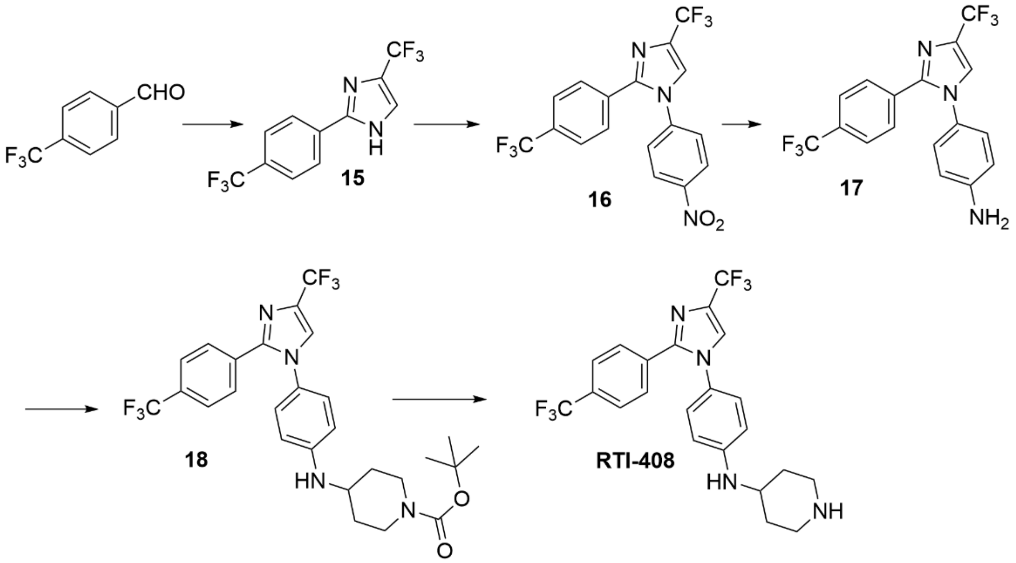

<u>15</u>: A mixture containing 1,1-Dibromo-3,3,3-trifluoroacetone (4.64 g, 17.2 mmol) and sodium acetate (1.88 g, 23.0 mmol) in water (42 mL) was heated to 95° C for 1 hour, then cooled to room temperature. 4'-Trifluoromethylbenzaldehyde (2.0 g, 11.4 mmol) in methanol (102 mL) was cooled to 0° C and treated with dropwise addition of ammonium hydroxide (50 mL). This mixture stirred for 10 minutes at 0° C and then the first mixture was added dropwise; the combined reaction slowly warmed to room temperature and stirred for 18 hours. The resulting solids were filtered, washed with water and dried and the filtrate was concentrated. The dried solids and filtrate residue were partitioned between dichloromethane and water. The organic layer was washed with brine, dried (Na_2SO_4) and concentrated. The crude material was adsorbed onto silica gel and purified via ISCO using 0-30% ethyl acetate from hexanes to yield 1.0 g (33%) of a yellow solid (**15**).

<u>16</u>: A mixture containing **15** (1.0 g, 3.57 mmol), 4-fluoronitrobenzene (1.52 g, 10.7 mmol) and K_2CO_3 (1.48 g, 10.7 mmol) in N, N-dimethylformamide (40 mL) was heated to 90° C for 21 hours. Upon cooling to room temperature, the poured into saturated aqueous LiCl solution and extracted with ethyl acetate. The combined organics were washed with water (5 x) and brine, dried ($MgSO_4$), filtered and concentrated. The crude material was adsorbed onto silica gel and purified via ISCO using 0-5% methanol from dichloromethane to yield 1.0 g (70%) of an off-white solid (**16**).

<u>17</u>: A mixture containing **16** (1.0 g, 2.49 mmol), stannous chloride dihydrate (3.77 g, 19.9 mmol) and concentrated HCl (4.15 mL, 49.9 mmol) in ethanol (40 mL) was heated to 50° C for 3.5 hours. Upon cooling to room temperature, the solvent was concentrated and the residue was diluted with ethyl acetate and 2 N NaOH. The aqueous layer was extracted with ethyl acetate and the combined organics were washed with water and brine, dried (Na_2SO_4), filtered and concentrated. The crude material was adsorbed onto silica gel and purified via ISCO using 0-5% methanol from dichloromethane to yield 275 mg (70%) of an off-white solid (**17**).

bioRxiv preprint doi: https://doi.org/10.1101/2025.11.04.686469; this version posted November 4, 2025. The copyright holder for this preprint (which was not certified by peer review) is the author/funder, who has granted bioRxiv a license to display the preprint in perpetuity. It is made available under aCC-BY 4.0 International license.

RTI-408: A mixture containing **17** (100 mg, 0.269 mmol), tert-Butyl 4-oxopiperidine-1-carboxylate (160 mg, 0.809 mmol) and anhydrous sodium sulfate (catalytic amount) in acetic acid (5 mL) was stirred at room temperature for 2 hours. Sodium triacetoxyborohydride (360 mg, 1.61 mmol) was added and the reaction stirred at room temperature for 18 hours. The reaction was concentrated and the residue was partitioned between ethyl acetate and saturated aqueous $NaHCO_3$. The crude material was adsorbed onto silica gel and purified via ISCO using 10-75% ethyl acetate from hexanes to yield 100 mg (0.181 mmol, 67%) of an off-white solid, which was dissolved in anhydrous dioxane (5 mL), cooled to 0° C and treated with 4.0 N hydrochloric acid in dioxane (2.25 mL, 9.03 mmol). The reaction warmed to room temperature and stirred for 18 hours. Upon completion, the mixture was concentrated and the residue was partitioned between dichloromethane and saturated aqueous $NaHCO_3$. The aqueous layer was extracted with dichloromethane and the combined organic layers were washed with brine, dried (Na_2SO_4), filtered and concentrated to obtain 25 mg (30%) of an off-white solid (**RTI-408**). ^{1}H-NMR ($CDCl_3$) δ 7.56 (d, 2 H, J = 8.0 Hz), 7.51 (d, 2 H, J = 8.4 Hz), 7.41 (s, 1 H), 7.00 (dd, 2 H, J = 6.8 Hz, 8.8 Hz), 6.62 (dd, 2 H, J = 8.8 Hz, 9.6 Hz), 3.78-3.61 (m, 4 H), 3.27-3.17 (m, 2 H), 3.12-3.01 (m, 1 H), 2.36-2.23 (m, 2 H).

Triazole Scaffold:

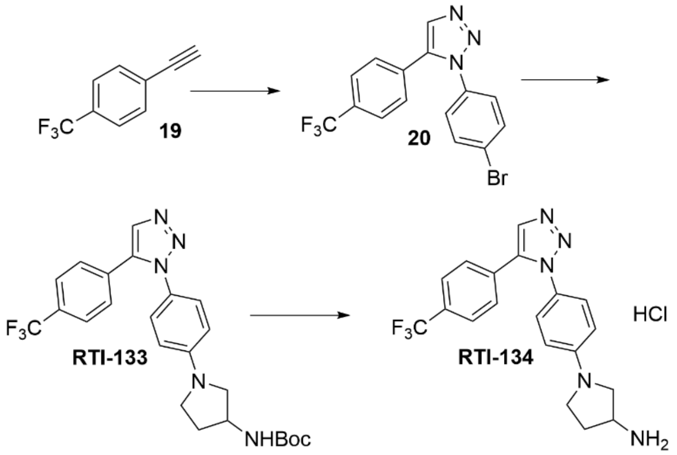

20: A mixture containing **19** (198 mg, 1.0 mmol) and 4-Trifluoromethylstyrene (179 mg, 1.05 mmol) in dimethylsulfoxide (3.3 mL) was treated with tetramethylammonium hydroxide (25% solution - 9.1 mg/37uL of water) and stirred at room temperature for 20 hours. The reaction was diluted with water (15 mL) and extracted with ethyl acetate. The organic layer was washed with water and brine, dried (Na_2SO_4), filtered and concentrated. The crude material was adsorbed onto silica gel and purified via ISCO using 5-30% ethyl acetate from hexanes to yield 350 mg (95%) of a yellow-brown solid (**20**).

RTI-133: The following were combined in a heavy-duty glass reactor: **20** (110 mg, 0.3 mmol), 3-N-Boc-aminopyrrolidine (72.4 mg, 0.39 mmol), BINAP (56 mg, 0.09 mmol), $Pd_2(dba)_3$ (27.5 mg, 0.03 mmol) and Cs_2CO_3 (127 mg, 0.39 mmol) in anhydrous toluene (3 mL) and nitrogen gas was bubbled into the mixture for two minutes. The reactor was then sealed with a Teflon cap and heated to 110° C for 15 hours. Upon cooling, the mixture was filtered through Celite and the filter pad was rinsed with ethyl acetate. The filtrate was washed with water and brine, dried (Na_2SO_4), filtered and concentrated. The crude material was purified over silica gel using 0-100% ethyl acetate from hexanes to yield 158.5 mg (>100%) of a yellow solid (**RTI-133**).

RTI-134: A solution of **RTI-133** (158.5 mg, 0.334 mmol) in dichloromethane (20 mL) was cooled to 0° C and treated with trifluoroacetic acid (0.5 mL, 6.73 mmol). The reaction warmed to room temperature and stirred for 15 hours. Upon completion, the mixture was concentrated and the residue was partitioned between ethyl acetate and 2 N aqueous NaOH. The aqueous layer was extracted with ethyl acetate and the combined organic layers were washed with brine, dried (Na_2SO_4), filtered and concentrated to yield 112.5 mg (90%) of a yellow solid,

bioRxiv preprint doi: https://doi.org/10.1101/2025.11.04.686469; this version posted November 4, 2025. The copyright holder for this preprint (which was not certified by peer review) is the author/funder, who has granted bioRxiv a license to display the preprint in perpetuity. It is made available under aCC-BY 4.0 International license.

which required no further purification. This material was dissolved in diethyl ether and treated with 2 N HCl/diethyl ether, stirred at room temperature for 18 hours, filtered, washed with diethyl ether and dried to yield 104 mg (76%) of **RTI-134** as a white solid. ^{1}H-NMR (CD_3OD) δ 8.08 (s, 1 H), 7.63 (d, 2 H, J = 8.0 Hz), 7.48 (d, 2 H, J = 8.4 Hz), 7.21 (d, 2 H, J = 8.8 Hz), 6.71 (d, 2 H, J = 8.8 Hz), 4.09-4.01 (m, 1 H), 3.72-3.61 (m, 2 H), 3.49-3.42 (m, 2 H), 2.54-2.44 (m, 2 H). LC-MS, calculated for $C_{19}H_{18}F_3N_5$ $(MH)^+$ 374.4; observed 374.2. Anal. Calculated (with 0.4 mol diethyl ether) for $C_{19}H_{19}ClF_3N_5$; C, 54.62; H, 5.45; N, 15.46. Found: C, 54.64; H, 5.05; N, 15.05.

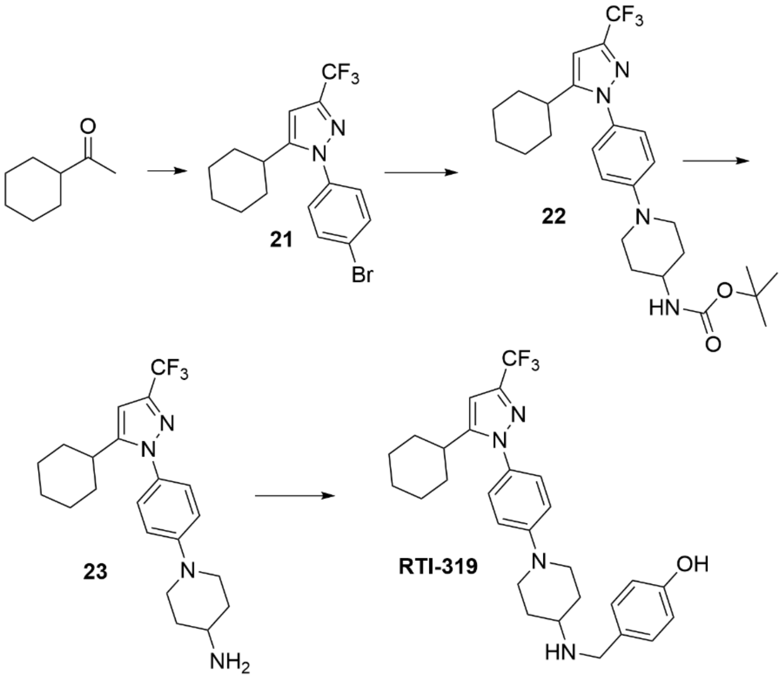

21: Into an oven dried flask was introduced sodium hydride (60% wt./mineral oil, 9.52 g, 237.6 mmol) and stirred in anhydrous THF (140 mL) for 5 minutes at room temperature. Ethyl trifluoroacetate (18.9 mL, 158.8 mmol) was added dropwise and this mixture stirred at room temperature for 10 minutes. A solution of cyclohexyl methyl ketone (10 g, 79.2 mmol) in anhydrous THF (40 mL) was added dropwise and the reaction mixture was refluxed for 3 hours. The reaction was concentrated and the residue was partitioned between ethyl acetate and water. The aqueous layer was extracted with ethyl acetate and the combined organics were washed with brine, dried (Na_2SO_4) and concentrated to yield a quantitative yield of a yellow oil, which was used without further purification. ^{1}H-NMR (CD_3OD) δ 5.24 (s, 1 H), 2.94-2.84 (m, 1 H), 1.82-1.66 (m, 4 H), 1.40-1.19 (m, 6 H).

A mixture containing the previous product (79.2 mmol) and 4-bromohydrazine hydrochloride (24 g, 103 mmol) in ethanol (1 L) was refluxed for 18 hours. The solvent was concentrated and the residue was partitioned between ethyl acetate and saturated aqueous $NaHCO_3$. The aqueous layer was extracted with ethyl acetate and the combined organics were washed with brine, dried ($MgSO_4$), filtered and concentrated. The crude material was purified over silica gel via a flush column using 3-5% ethyl acetate from hexanes to remove baseline impurities from the isomeric product mixture. Upon sitting at room temperature, a yellow solid (undesired isomer) precipitated from the mixture. The liquid portion of the mixture (rich in **21**) was decanted and the yellow solid was triturated in hexanes to remove all of **21** (with some of the undesired isomer) and filtered. This combined filtrate and decantate was concentrated, adsorbed onto silica gel and purified via ISCO using 2-15% dichloromethane from hexanes to yield 4.58 g (15%) of an orange oil (**21**). ^{1}H-NMR ($CDCl_3$) δ 7.59 (d, 2 H, J = 9 Hz), 7.37 (d, 2 H, J = 9 Hz), 6.62 (s, 1 H), 2.75-2.66 (m, 1 H), 2.09-1.99 (m, 2 H), 1.90-1.70 (m, 2 H), 1.53-1.21 (m, 6 H). Anal. Calculated for $C_{16}H_{16}BrF_3N_2$; C, 51.48; H, 4.32; N, 7.50. Found: C, 51.73; H, 4.51; N, 7.51.

bioRxiv preprint doi: https://doi.org/10.1101/2025.11.04.686469; this version posted November 4, 2025. The copyright holder for this preprint (which was not certified by peer review) is the author/funder, who has granted bioRxiv a license to display the preprint in perpetuity. It is made available under aCC-BY 4.0 International license.

22: The following were combined in a heavy-duty glass reactor: **21** (1.1 g, 2.95 mmol), 4-(tert-butoxycarbonylamino)piperidine (1.18 g, 5.89 mmol), BINAP (551 mg, 0.884 mmol), $Pd_2(dba)_3$ (540 mg, 0.589 mmol) and Cs_2CO_3 (1.92 g, 5.89 mmol) in anhydrous toluene (40 mL) and nitrogen gas was bubbled into the mixture for two minutes. The reactor was then sealed with a Teflon cap and heated to 110° C for 15 hours. Upon cooling, the mixture was filtered through Celite and the filter pad was rinsed with ethyl acetate. The filtrate was washed with water and brine, dried (Na_2SO_4), filtered and concentrated to obtain 3.51 g of a rust-colored gel. The crude material was purified over silica gel using 0-100% ethyl acetate from hexanes to yield 426 mg (29%) of a yellow solid (**22**). ^{1}H-NMR ($CDCl_3$) δ 7.31 (d, 2 H, J = 9 Hz), 6.93 (d, 2 H, J = 9 Hz), 6.55 (s, 1 H), 4.48 (br s, 1 H), 3.73-3.64 (m, 3 H), 2.91 (dd, 2 H, J = 3 Hz, 9 Hz), 2.74-2.67 (m, 1 H), 2.07-2.00 (m, 4 H), 1.84-1.67 (m, 7 H), 1.62-1.25 (m, 7 H), 1.46 (s, 9 H).

23: A solution of **22** (426 mg, 0.865 mmol) in dichloromethane (17 mL) was cooled to 0° C and treated with trifluoroacetic acid (0.65 mL, 8.75 mmol). The reaction warmed to room temperature and stirred for 15 hours. Upon completion, the mixture was concentrated and the residue was partitioned between ethyl acetate and 2 N aqueous NaOH. The aqueous layer was extracted with ethyl acetate and the combined organic layers were washed with brine, dried (Na_2SO_4), filtered and concentrated to yield 281 mg of **23** (>100%) of a yellow gel, which required no further purification. ^{1}H-NMR ($CDCl_3$) δ 7.30 (d, 2 H, J = 9 Hz), 6.94 (d, 2 H, J = 9 Hz), 6.55 (s, 1 H), 3.75-3.69 (m, 2 H), 2.89-2.80 (m, 2 H), 2.73-2.66 (m, 1 H), 2.10-1.71 (m, 7 H), 1.53-1.27 (m, 8 H). LC-MS, calculated for $C_{21}H_{27}F_3N_4$ $(MH)^+$ 393.4; observed 393.0.

RTI-319: A solution of **23** (262 mg, 0.667 mmol), 4-hydroxybenzaldehyde (83 mg, 0.68 mmol) and 4A molecular sieves (300 mg) in anhydrous methanol (7.5 mL) and anhydrous tetrahydrofuran (3.5 mL), was stirred at room temperature for 18 hours. The reaction was cooled to 0° C and treated with sodium borohydride (51 mg, 1.33 mmol); the reaction stirred for 4 hours at room temperature. The reaction was concentrated and the residue partitioned between saturated aqueous sodium bicarbonate solution and ethyl acetate. The combined organic layers were washed with brine, dried (Na_2SO_4), filtered and concentrated to yield 390 mg of a yellow gel. The crude material was purified over silica gel using 0.5-5 % methanol from dichloromethane to yield 146 mg (44%) of **RTI-319** as a white solid. ^{1}H-NMR ($CDCl_3$) δ 7.30 (d, 2 H, J = 9 Hz), 7.17 (d, 2 H, J = 9 Hz), 6.93 (d, 2 H, J = 9 Hz), 6.72 (d, 2 H, J = 9 Hz), 6.55 (s, 1 H), 3.77 (s, 2 H), 3.72 (dd, 2 H, J = 12 Hz), 2.80 (dd, 2 H, J = 12 Hz), 2.74-2.65 (m, 2 H), 2.36 (br s, 1 H), 2.02 (d, 4 H, J = 12 Hz), 1.89-1.71 (m, 3 H), 1.62-1.22 (m, 7 H). ^{13}C NMR ($CDCl_3$, 75 MHz) δ 151.5, 129.4, 126.5, 115.6, 115.4, 54.0, 50.2, 47.9, 37.3, 33.0, 32.1, 26.2, 26.0; ESI-MS, calculated for $C_{28}H_{33}F_3N_4O$ $(M)^-$ 497.6; observed 497.2. Anal. Calculated for $C_{28}H_{33}F_3N_4O$; C, 67.45; H, 6.67; N, 11.23. Found: C, 67.19; H, 6.63; N, 11.11.

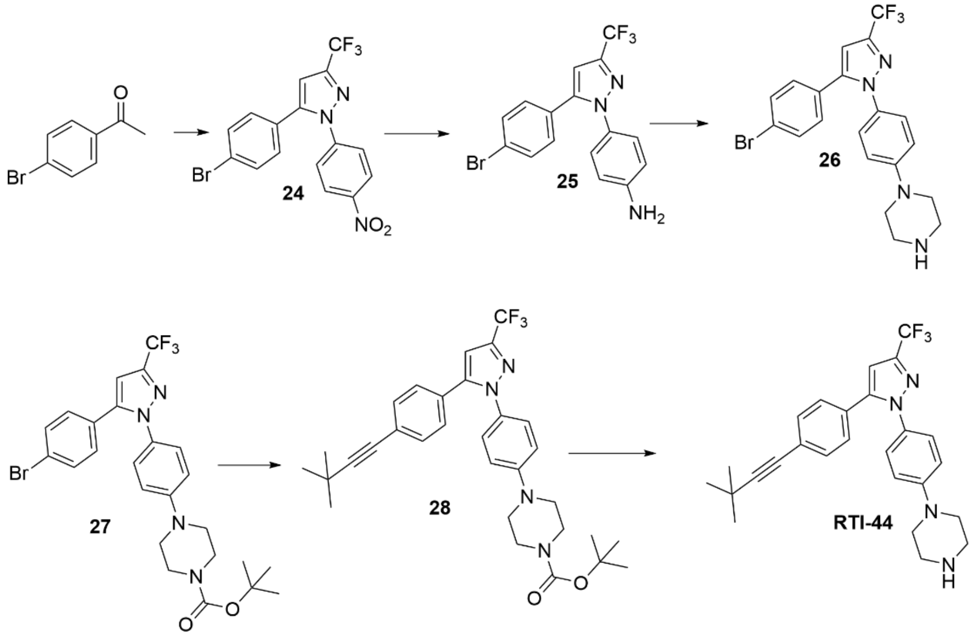

bioRxiv preprint doi: https://doi.org/10.1101/2025.11.04.686469; this version posted November 4, 2025. The copyright holder for this preprint (which was not certified by peer review) is the author/funder, who has granted bioRxiv a license to display the preprint in perpetuity. It is made available under aCC-BY 4.0 International license.

24: To a suspension of sodium hydride (60% wt./mineral oil, 1.69 g, 70.3 mmol) and stirred in anhydrous THF (50 mL) in a 3-neck flask fitted with a reflux condenser, ethyl trifluoroacetate (7.14 g, 50.2 mmol), in 6 mL of anhydrous THF, was added dropwise; this mixture stirred at room temperature for 10 minutes. A solution of p-Bromoacetophenone (5 g, 25.1 mmol) in anhydrous THF (25 mL) was added dropwise, over 30 minutes, and the reaction mixture was refluxed for 3 hours. The reaction was quenched acetic acid/water (5 mL, 1/1 ratio) and concentrated. The residue was partitioned between ethyl acetate and acetic acid/water and the combined organics were washed with brine, dried (Na_2SO_4) and concentrated to generate 6.5 g (88%) of a yellow solid, which was used without further purification.

A mixture containing the previous product (0.733 g, 2.5 mmol) and 4-nitrohydrazine hydrochloride (0.57 g, 3.0 mmol) in ethanol (25 mL) was refluxed for 2 hours. Another 47 mg (0.25 mmol) of 4-nitrohydrazine hydrochloride was added and the reaction refluxed for 15 hours. The solvent was concentrated and the residue was partitioned between ethyl acetate and water. The aqueous layer was extracted with ethyl acetate and the combined organics were washed with brine, dried ($MgSO_4$), filtered and concentrated. The crude material was adsorbed onto silica gel and purified via ISCO using 0.5-10% ethyl acetate from hexanes to yield 0.69 g (67%) of a white solid (**24**).

25: A mixture containing **24** (82.4 mg, 0.20 mmol), stannous chloride dihydrate (133 mg, 0.70 mmol) and concentrated HCl (0.3 mL, 3.6 mmol) in ethanol (1.5 mL) was heated to 50° C for 2.5 hours. Upon cooling to room temperature, the solvent was concentrated and the residue was diluted with ethyl acetate and 2 N NaOH. The aqueous layer was extracted with ethyl acetate and the combined organics were washed with water and brine, dried (Na_2SO_4), filtered and concentrated to yield 75 mg (98%) of an off-white solid (**25**), which required no further purification.

26: A mixture containing **25** (38.2 mg, 0.10 mmol), Bis(2-chloroethyl)amine hydrochloride (35.6 mg, 0.20 mmol) and K_2CO_3 (27.6 mg, 0.20 mmol) in 1-methoxyethanol (1.0 mL) was heated to 175° C for 2 hours via microwaves. The reaction was heated for another 1 hour to enhance completion. Upon cooling to room temperature, the reaction was diluted with 2 N NaOH. The mixture was extracted with ethyl acetate and the combined organics were washed with water and brine, dried (Na_2SO_4), filtered and concentrated. The crude material was adsorbed onto silica gel and purified via ISCO using 0-15% (1% NH_4OH/methanol) from dichloromethane to yield 27 mg (60%) of a colorless oil (**26**).

27: A mixture containing **26** (44 mg, 0.097 mmol), di-tert-butyl dicarbonate (23.4 mg, 0.107 mmol) in ethanol (1.0 mL) was heated to 30° C for 30 minutes. The reaction was concentrated and diluted with water. The mixture was extracted with ethyl acetate and the combined organics were washed with water and brine, dried (Na_2SO_4), filtered and concentrated. The crude material was adsorbed onto silica gel and purified via ISCO using 0-20% ethyl acetate from hexanes to yield 29 mg (54%) of a colorless oil (**27**).

28: The following were combined in an I-CHEM vial with a screw cap septum: **27** (43.8 mg, 0.08 mmol), 3,3-Dimethyl-1-butyne (16 mg, 0.16 mmol), Palladium tetrakis(triphenylphosphine) (9.2 mg, 0.008 mmol), CuI (3.0 mg, 0.016 mmol) in diisopropylamine (2 mL). The vial was repeatedly evacuated and backfilled with nitrogen. The reaction was heated to 70° C for 15 hours. Upon cooling, the mixture was filtered through Celite and the filter pad was rinsed with ethyl acetate. The filtrate was concentrated and the crude material was purified over silica gel using 0-18% ethyl acetate from hexanes to yield 43 mg (94%) of a colorless oil (**28**).

RTI-44: A solution of **28** (34 mg, 0.061 mmol) in dichloromethane (2 mL) was cooled to 0° C and treated with trifluoroacetic acid (0.2 mL, 2.69 mmol). The reaction warmed to room temperature and stirred for 15 hours. Upon completion, the mixture was concentrated and the residue was partitioned between ethyl acetate and 2 N aqueous NaOH. The aqueous layer was extracted with ethyl acetate and the combined organic layers were washed with brine, dried (Na_2SO_4), filtered and concentrated to yield 281 mg of **RTI-44** (>100%) of a yellow gel, which required no further purification. ^{1}H-NMR (CD_3OD) δ 7.67-7.61 (m, 2 H), 7.58-7.52 (m, 2 H), 7.30-7.17 (m, 4 H), 3.49-3.43 (m, 4 H), 3.37-3.32 (m, 4 H), 1.27 (s, 9 H).
